# A prognostic neural epigenetic signature in high-grade glioma

**DOI:** 10.1038/s41591-024-02969-w

**Published:** 2024-05-17

**Authors:** Richard Drexler, Robin Khatri, Thomas Sauvigny, Malte Mohme, Cecile L. Maire, Alice Ryba, Yahya Zghaibeh, Lasse Dührsen, Amanda Salviano-Silva, Katrin Lamszus, Manfred Westphal, Jens Gempt, Annika K. Wefers, Julia E. Neumann, Helena Bode, Fabian Hausmann, Tobias B. Huber, Stefan Bonn, Kerstin Jütten, Daniel Delev, Katharina J. Weber, Patrick N. Harter, Julia Onken, Peter Vajkoczy, David Capper, Benedikt Wiestler, Michael Weller, Berend Snijder, Alicia Buck, Tobias Weiss, Pauline C. Göller, Felix Sahm, Joelle Aline Menstel, David Niklas Zimmer, Michael B. Keough, Lijun Ni, Michelle Monje, Dana Silverbush, Volker Hovestadt, Mario L. Suvà, Saritha Krishna, Shawn L. Hervey-Jumper, Ulrich Schüller, Dieter H. Heiland, Sonja Hänzelmann, Franz L. Ricklefs

**Affiliations:** 1 Department of Neurosurgery, University Medical Center Hamburg-Eppendorf, Hamburg, Germany; 2 Department of Neurology, Stanford University, Stanford, CA USA; 3 Institute of Medical Systems Biology, University Medical Center Hamburg-Eppendorf, Hamburg, Germany; 4 Center for Biomedical AI, University Medical Center Hamburg-Eppendorf, Hamburg, Germany; 5 Institute of Neuropathology, University Medical Center Hamburg-Eppendorf, Hamburg, Germany; 6 Mildred Scheel Cancer Career Center HaTriCS4, University Medical Center Hamburg-Eppendorf, Hamburg, Germany; 7 Center for Molecular Neurobiology Hamburg (ZMNH), University Hospital Hamburg Eppendorf, Hamburg, Germany; 8 Research Institute Children’s Cancer Center Hamburg, Hamburg, Germany; 9 III. Department of Medicine, University Medical Center Hamburg-Eppendorf, Hamburg, Germany; 10 Hamburg Center for Kidney Health (HCKH), University Medical Center Hamburg-Eppendorf, Hamburg, Germany; 11 Department of Neurosurgery, University Hospital Aachen, Aachen, Germany; 12 Department of Neurosurgery, University Clinic Erlangen, Friedrich-Alexander Universität Erlangen-Nürnberg, Erlangen, Germany; 13 Neurological Institute (Edinger Institute), University Hospital Frankfurt, Frankfurt am Main, Germany; 14 German Cancer Consortium (DKTK), Heidelberg, Germany and German Cancer Research Center (DKFZ), Heidelberg, Germany; 15 Frankfurt Cancer Institute (FCI), Frankfurt am Main, Germany; 16 University Cancer Center (UCT) Frankfurt, Frankfurt am Main, Germany; 17 Institute of Neuropathology, Faculty of Medicine, LMU Munich, Munich, Germany; 18 Department of Neurosurgery, Charité - Universitätsmedizin Berlin, Berlin, Germany; 19 Department of Neuropathology, Charité - Universitätsmedizin Berlin, Corporate Member of Freie Universität Berlin and Humboldt-Universität zu Berlin, Berlin, Germany; 20 Department of Neuroradiology, Klinikum rechts der Isar, School of Medicine, Technical University Munich, Munich, Germany; 21 Department of Neurology, Clinical Neuroscience Center, University Hospital Zurich, Zurich, Switzerland; 22 Department of Neurology, University of Zürich, Zurich, Switzerland; 23 Institute of Molecular Systems Biology, ETH Zurich, Zurich, Switzerland; 24 Hopp Children’s Cancer Center Heidelberg (KiTZ), Heidelberg, Germany; 25 Department of Neuropathology, University Hospital Heidelberg, Heidelberg, Germany; 26 Department of Neurosurgery, Medical Center University of Freiburg, Freiburg, Germany; 27 Department of Cancer Biology, Perelman School of Medicine, University of Pennsylvania, Philadelphia, PA USA; 28 Abramson Family Cancer Research Institute, Perelman School of Medicine, University of Pennsylvania, Philadelphia, PA USA; 29 Broad Institute of Harvard and MIT, Cambridge, MA USA; 30 Department of Pediatric Oncology, Dana-Farber Cancer Institute, Boston, MA USA; 31 Department of Pathology and Center for Cancer Research, Massachusetts General Hospital and Harvard Medical School, Boston, MA USA; 32 Department of Neurological Surgery, University of California, San Francisco, CA USA; 33 Department of Pediatric Hematology and Oncology, Research Institute Children’s Cancer Center Hamburg, University Medical Center Hamburg-Eppendorf, Hamburg, Germany; 34 Translational Neurosurgery, Friedrich-Alexander Universität Erlangen-Nürnberg, Erlangen, Germany; 35 Department of Neurological Surgery, Northwestern University Feinberg School of Medicine, Chicago, IL USA; 36 German Cancer Consortium (DKTK), Partner Site Freiburg, Freiburg, Germany

**Keywords:** Translational research, CNS cancer, DNA methylation

## Abstract

Neural–tumor interactions drive glioma growth as evidenced in preclinical models, but clinical validation is limited. We present an epigenetically defined neural signature of glioblastoma that independently predicts patients’ survival. We use reference signatures of neural cells to deconvolve tumor DNA and classify samples into low- or high-neural tumors. High-neural glioblastomas exhibit hypomethylated CpG sites and upregulation of genes associated with synaptic integration. Single-cell transcriptomic analysis reveals a high abundance of malignant stemcell-like cells in high-neural glioblastoma, primarily of the neural lineage. These cells are further classified as neural-progenitor-cell-like, astrocyte-like and oligodendrocyte-progenitor-like, alongside oligodendrocytes and excitatory neurons. In line with these findings, high-neural glioblastoma cells engender neuron-to-glioma synapse formation in vitro and in vivo and show an unfavorable survival after xenografting. In patients, a high-neural signature is associated with decreased overall and progression-free survival. High-neural tumors also exhibit increased functional connectivity in magnetencephalography and resting-state magnet resonance imaging and can be detected via DNA analytes and brain-derived neurotrophic factor in patients’ plasma. The prognostic importance of the neural signature was further validated in patients diagnosed with diffuse midline glioma. Our study presents an epigenetically defined malignant neural signature in high-grade gliomas that is prognostically relevant. High-neural gliomas likely require a maximized surgical resection approach for improved outcomes.

## Abstract (web summary)

A neural epigenetic signature detectable via plasma analyses is prognostic in patients with glioblastoma, resembling an oligodendrocyte-progenitor- and neuronal-progenitor-cell-like state and showing increased neuro-to-glioma synapse formation.

## Main

The importance of the nervous system as a regulator of brain tumors has been repeatedly highlighted but has not yet been translated into a therapeutically relevant setting^1–5^. Particularly in gliomas, studies have demonstrated that the activity-driven formation of malignant neuron-to-glioma networks is critical for cancer progression^4,6–8^, and that glioma cells remodel neuronal circuits by increasing neuronal hyperexcitability^4,9–12^. Further insight into molecular mechanisms identified connected and unconnected glioblastoma cells that form distinct cell states and differ in their gene signatures as well as functions within neuron-to-glioma networks^13^. Additionally, glioblastomas exhibiting high functional connectivity have been shown to be associated with poorer survival^12^. Moreover, callosal projection neurons were shown to promote glioma progression and widespread infiltration underpinning the importance of the central nervous system as a critical regulator^14^.

High-grade glioma consists of both malignant and nonmalignant cells^15,16^. Therefore, their cell-type composition can be determined through epigenetic bulk DNA analysis, which allows for the identification of molecular differences. Here, we aimed to use brain tumor-related epigenetic signatures to understand isocitrate dehydrogenase (IDH)-wild-type high-grade gliomas, suggesting that certain epigenetic subclasses may be more likely to be integrated into neuron-to-glioma networks with clinical relevance. We analyzed the epigenetic neural signature of central nervous system (CNS) tumors, categorizing glioblastoma and H3K27-altered diffuse midline glioma (DMG) into low- and high-neural subgroups, which were characterized molecularly, functionally and clinically.

## Results

### Epigenetic neural signature predicts patients outcome

**Fig. 1:**
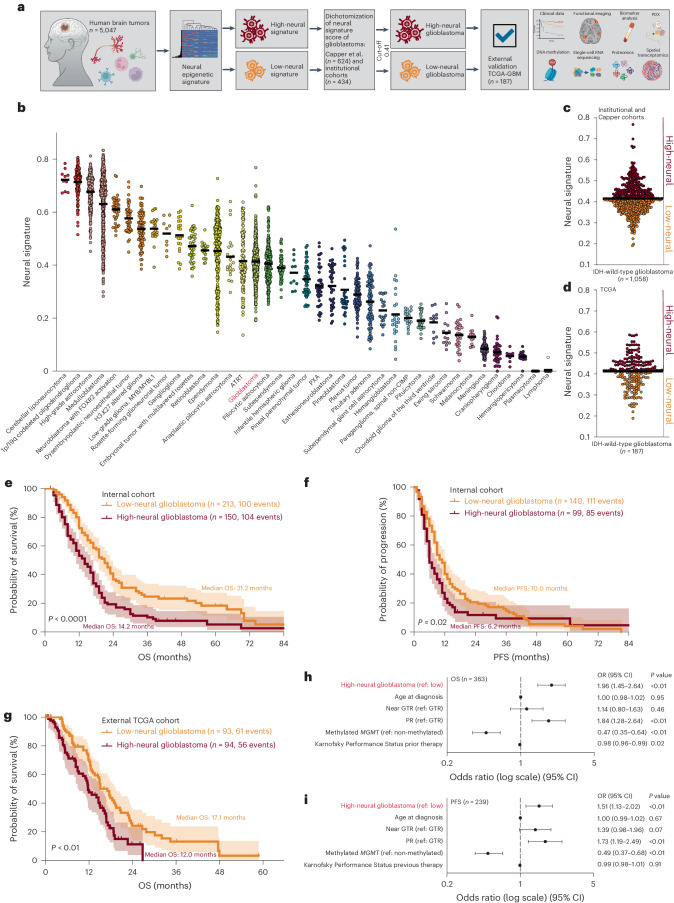
Epigenetic neural classification predicts outcome of patients with glioblastoma. **a**, Schematic of the study workflow. In humans (*n* = 5,047) diagnosed with a CNS tumor we performed deconvolution using DNA methylation arrays (850k or 450k) for determining the neural signature. IDH-wild-type glioblastomas were stratified into subgroups with a low- or high-neural signature for further analyses. **b**, Epigenetic neural signature in all CNS tumor entities (*n* = 5,047). **c**, Dichotomization of the combined dataset from Capper et al.^18^ and three institutional cohorts (Hamburg, Berlin and Frankfurt, all Germany) into low- and high-neural glioblastomas. The black line indicates a median neural score of all included patients with glioblastoma (*n* = 1,058) and represents the cutoff (0.41) for stratification into low- and high-neural glioblastoma. **d,** External validation of the cutoff value using the TCGA-GBM dataset (*n* = 187). The black line indicates the median neural score. **e**–**i**, Survival analysis of patients with low- and high-neural glioblastoma treated by radiochemotherapy after surgery. **e**, Overall survival (OS) of 363 patients with glioblastoma of the internal clinical cohort. log-rank test, *P* = 0.000005. Error bands represent 95% CI. **f**, PFS of 226 patients with glioblastoma of the internal clinical cohort. log-rank test, *P* = 0.0233. Error bands represent 95% CI. **g**, Overall survival of 187 patients with glioblastoma of the TCGA-GBM cohort. log-rank test, *P* = 0.0017. Error bands represent 95% CI. **h**,**i**, Forest plots illustrating multivariate analysis of patients with glioblastoma from the internal clinical cohort. Means are shown by closed circles and whiskers represent 95% CI. GTR, gross total resection; PR, partial resection; MGMT, *O*^6^-methylguanine-DNA-methyltransferase. Source data

**Extended Data Fig. 1:**
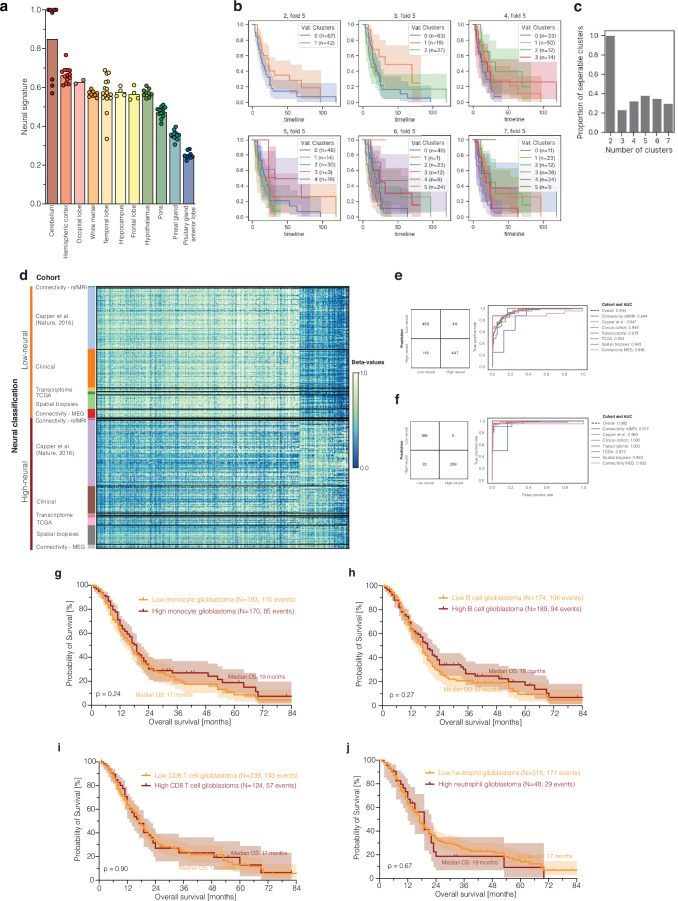
Implementation of the epigenetic neural signature and validation of low- and high-neural subclassification of glioblastoma samples. **a)**. Epigenetic neural signature in healthy brain tissues obtained from the Capper dataset^40^. **b, c)**. Analysis of different number of neural clusters that can predict differential survival outcome in the clinical cohort (n=363) by using 10-fold cross-validation with Kmeans. The figure displays Kaplan–Meier curves of the clusters in the validation set of the 5^th^ fold. The survival curves demonstrate that the best results are obtained with two clusters (low- versus high-neural). Log rank test was used for the survival difference between the clusters. *Error bands representing the 95% confidence interval*. **d)**. Validation of the cut off for the neural signature across multiple cohorts used in the manuscript. Beta-values for CpGs differentially methylated between the low-neural and high-neural groups. The selection was made using the clinical cohort (n=363). **e)**. Using the clinical cohort as the training set, a logistic regression model was trained. The logistic regression model trained on the clinical cohort on the identified signature classifies across cohorts with overall AUC of 0.944 and > 0.84 in all cohorts. **f)**. Same as in e.) but a threshold on the prediction score was set (0.9) to keep only high confidence predictions. The AUC of the classifier is > 0.91 in the external cohorts when using only high probability predictions. **g, j)**. Survival analysis of patients with glioblastoma applying brain tumor-related cell signatures of the Moss signature. *Log-rank test, g) P = 0.2415, h.) P = 0.2703, i) P = 0.9010, j) P = 0.6646. Error bands representing the 95% confidence interval. OS: overall survival*.

To address our hypotheses, we applied the epigenetic neural signature of Moss et al.^17^ to estimate cellular composition (Fig. 1a) of a combined dataset of epigenetically profiled CNS tumors of Capper et al.^18^ and our institutional cohorts (Fig. 1b) as well as healthy tissue (Extended Data Fig. 1a). Using this combined dataset, glioblastoma samples (*n* = 1,058) were dichotomized for defining a cutoff separating low- and high-neural tumors (cutoff based on median neural proportion 0.41; Fig. 1c,d). We demonstrate that more than two clusters did not show significant separability of survival among the resulting clusters (Extended Data Fig. 1b,c). The reproducibility of the cutoff (0.41) was validated across multiple cohorts (Extended Data Fig. 1d–f). The cutoff was applied to 363 patients with glioblastoma from our clinical cohort who received surgical treatment followed by standard-of-care combined chemoradiotherapy. Survival analysis revealed a significantly shorter overall survival (*P* < 0.0001, median overall survival 14.2 versus 21.2 months; Fig. 1e) and progression-free survival (PFS) (*P* = 0.02, median PFS 6.2 versus 10.0 months; Fig. 1f) for patients with a high-neural glioblastoma (Extended Data Table 1). This finding was replicated in an external cohort with 187 patients from The Cancer Genome Atlas (TCGA)-GBM database^19^ (*P* < 0.01, median overall survival 12.0 versus 17.1 months; Fig. 1g). The neural classification was identified as an independent prognostic factor for overall survival (odds ratio (OR) 1.96; 95% confidence interval (CI) 1.45–2.64, *P* < 0.01; Fig. 1h) and PFS (OR 1.51; 95% CI 1.13–2.02, *P* < 0.01; Fig. 1i). Other infiltrating brain tumor cell types of the lymphoid or myeloid lineage did not show an association with patient survival (Extended Data Fig. 1g–j).

### High-neural glioblastomas exhibit a synaptic character

**Extended Data Fig. 2:**
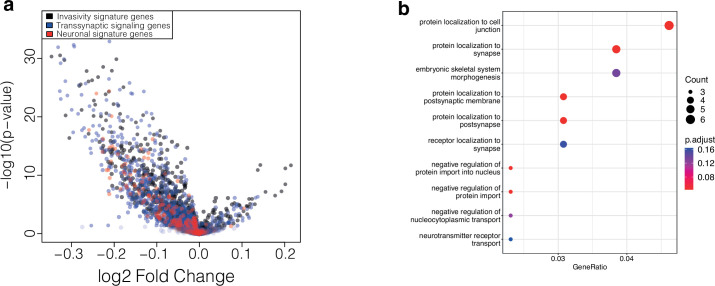
Differentially methylated CpG sites of high- and low-neural glioblastomas. **a)**. Volcano plot showing differentially methylated CpG sites of genes of the invasivity signature, neuronal signature, and trans-synaptic signaling signature in high-neural glioblastoma. **b)**. Gene set enrichment analysis of differentially methylated CpG sites in high-neural glioblastoma compared to low-neural glioblastoma samples.

To discern epigenetic differences in low- and high-neural glioblastomas, we applied the ‘invasivity signature’^13^ (172 genes linked to neural features, migration and invasion) to the DNA methylation data of our clinical cohort (Supplementary Table). High-neural tumors were hypomethylated at CpG sites within gene loci of the invasivity signature compared to low-neural tumors (Extended Data Fig. 2a). In addition, two gene sets that are either associated with neuron-to-glioma synapse formation^20^ (‘neuronal signature genes’; Supplementary Table) or trans-synaptic signaling^21^ (‘trans-synaptic signaling genes’; Supplementary Table) were hypomethylated in high-neural glioblastomas (Extended Data Fig. 2a), whereas synapse-related genes were upregulated in high-neural glioblastoma (Extended Data Fig. 2b).

**Fig. 2:**
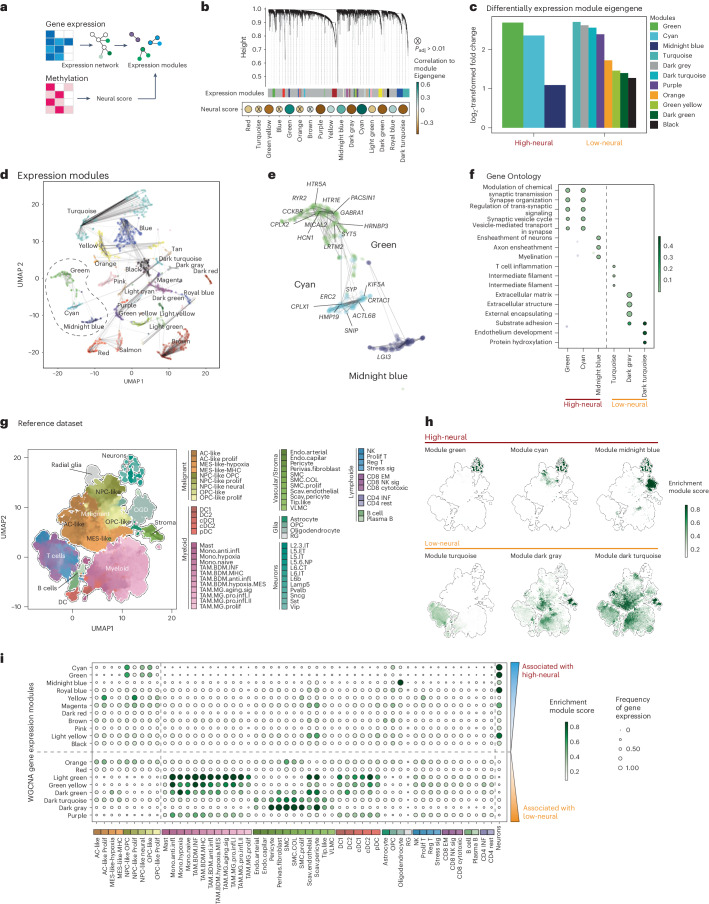
Integrated epigenetic and transcriptomic analysis reveals synaptic functions and a malignant NPC/OPC-like character in high-neural glioblastoma. **a**, Illustration of the workflow to integrate epigenetic and transcriptional data. Gene co-regulation networks are correlated to the epigenetic deconvolution signature. **b**, Hierarchical dendrogram of the gene expression modules derived from the weighted correlation network analysis. Dot-plot of the neural signature with gene expression models using Pearson correlation (bottom). Size and color indicate the correlation coefficient, nonsignificant correlation is marked. **c**, Bar-plot of the differential gene expression of module eigengenes (log_2_-transformed fold change) in low- and high-neural glioblastoma (cutoff 0.41). **d**, Dimensional reduction (UMAP) of the gene expression modules (named by colors). **e**, A detailed visualization of the modules: green, cyan and midnight blue (significantly associated with high-neural tumors). **f**, Gene Ontology analysis of gene expression modules in low- and high-neural tumors. **g**, UMAP dimensional reduction of the GBMap reference dataset. Colors indicate the different cell types. **h**, Module eigengene expression of low- and high-neural glioblastoma in the GBMap reference dataset. **i**, Gene expression enrichment of low- and high-neural-associated module eigengenes across glioblastoma cell states. AC, astrocytes; DC, dendritic cells; GBM, glioblastoma; NK, natural killer; OGD, oligodendrocytes; TAM, tumor-associated macrophages. Source data

Next, we used an integrative analysis of paired epigenetic and transcriptomic datasets of glioblastoma samples (*n* = 86). First, we computed a scale-free gene expression network (weighted correlation network analysis; WGCNA^22^) resulting in gene expression modules, which were further correlated to the neural signature through module significance measurement by quantifying the absolute correlation between the epigenetic signature and the individual module-derived gene expression profiles (Fig. 2a,b). We identified three expression modules significantly correlated with the epigenetic status of high-neural glioblastomas: module green (*R*^2^ = 0.55, *P* = 3.5 × 10^−6^), module cyan (*R*^2^ = 0.67, *P* < 2.2 × 10^−22^) and module midnight blue (*R*^2^ = 0.41, *P* = 9.3 × 10^−5^) (Fig. 2c,d). Gene Ontology analysis revealed that these modules were associated with synaptic functions (*GRIN3A*, *SYT4* and *SNAP25*), regulating the expression of genes involved in neuronal differentiation (*NEUROD2*) and calcium-dependent cell adhesion (*CDH22*, *CNTNAP5* and *CNTN3*) (Fig. 2e,f).

**Extended Data Fig. 3:**
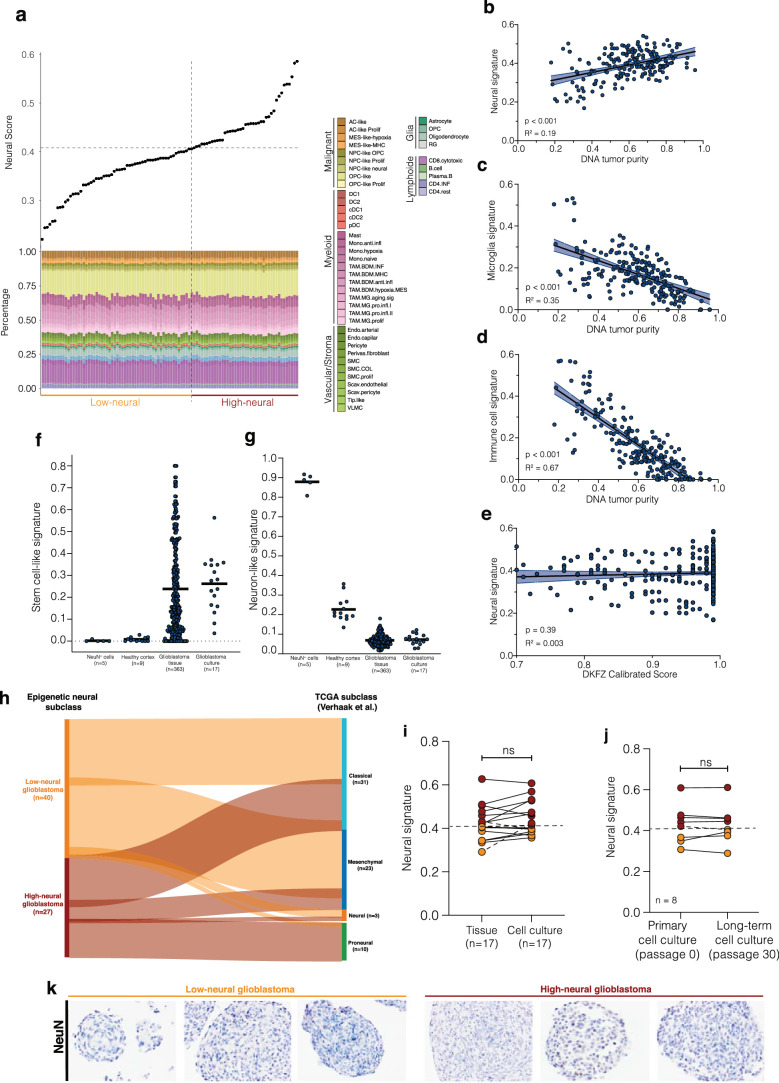
Quality measurements and reliability of the epigenetic neural signature in glioblastoma samples. **a**). Integrated analysis of the individual patients' neural scores and the corresponding cell proportions obtained from RNA sequencing deconvolution. **b)**. Correlation between the neural signature and DNA tumor purity. *Simple linear regression P = 0.000000000063765, error bands representing the 95% confidence interval*. **c)**. Correlation between the microglia signature and DNA tumor purity. *Simple linear regression P = 0.00000000041872, error bands representing the 95% confidence interval*. **d)**. Correlation between the immune cell signature and DNA tumor purity. *Simple linear regression P = 0.000000000019814, error bands representing the 95% confidence interval*. **e)**. Correlation between the DKFZ calibrated score for the diagnosis ‘IDH-wild-type glioblastoma’ and the neural signature. *Simple linear regression P = 0.2803, error bands representing the 95% confidence interval*. **f, g)**. Single-cell deconvolution of DNA methylation profiles compare **f)**. stem cell-like and **g)**. neuron-like signatures in NeuN^+^ cells, healthy cortex, glioblastoma tissue samples, and glioblastoma cell cultures. **h)**. Overlap between the epigenetic neural classification and TCGA subtypes after integrated RNA sequencing analysis. **i)**. Comparison of neural signature between patient’s tumor tissue and cell culture in 17 glioblastomas. *Two-sided t-test P = 0.2593*. **j)**. Stability of the epigenetic neural signature during long-term cell culturing. Data were obtained from a publicly available dataset (n =6, GSE181314) and in-house (n = 1). *Two-sided t-test P = 0.8471*. **k)**. Demonstration of NeuN^+^ staining in glioblastoma neurospheres. *n=15 biological replicates*.

We projected module eigengene signatures onto an integrated single-cell dataset of malignant (GBMap^23^) and healthy brain cells from the motor cortex (Allen Brain Institute). This analysis revealed a significant enrichment of the corresponding expression modules clustering to cells of the neural lineage such as healthy neurons along with malignant neural-progenitor-like cells (NPCs) and oligodendrocyte-progenitor-like cells (OPCs) (module green and cyan, *P* < 0.01), as well as nonmalignant oligodendrocytes (module midnight blue, *P* < 0.01) (Fig. 2g–i and Extended Data Fig. 3a). This correlation with the signature, dominated by typical neuronal marker genes, was anticipated. To assess whether the neural signature in our samples reflects malignant cell properties or merely the presence of neurons, we analyzed the relationship between DNA purity and the neural signature, finding a notable positive correlation (*P* < 0.001, *R*^2^ = 0.19; Extended Data Fig. 3b), whereas microglia (*P* < 0.001, *R*^2^ = 0.35; Extended Data Fig. 3c) and immune cell signatures (*P* < 0.001, *R*^2^ = 0.67; Extended Data Fig. 3d) showed a negative correlation. Our study, using only glioblastoma samples with a reliable diagnostic output from the DKFZ methylation classifier (Methods) showed that the calibrated score for ‘IDH-wild-type glioblastoma’ was unaffected by the epigenetic neural signature, nor vice versa (*P* = 0.39, *R*^2^ = 0.003; Extended Data Fig. 3e). Additionally, a non-reference-based multi-dimensional single-cell deconvolution algorithm^24^ was used to differentiate the neural signature in tumor cells from neuronal contamination. The analysis, which included glioblastoma tissue, matching tumor monocultures (*n* = 17), healthy cortex (*n* = 9) and sorted NeuN^+^ cells (*n* = 5), confirmed a higher stem-cell-like signature in glioblastoma tissue and cell cultures (Extended Data Fig. 3f) and the distinct neuronal signature in NeuN^+^ cells and healthy cortex (Extended Data Fig. 3g). Integrating RNA sequencing (RNA-seq) data, we observed 64 out of 67 samples (95.52%; Extended Data Fig. 3h) clustered into the established Verhaak transcriptomic glioblastoma subtypes (classical, mesenchymal and proneural)^25^. Ultimately, we analyzed the neural signature in cell cultures from 17 freshly resected patients with glioblastoma and observed a well-preserved neural signature (Extended Data Fig. 3i), which remained stable even in long-term cultures (Extended Data Fig. 3j) without the presence of NeuN^+^ cells (Extended Data Fig. 3k).

**Extended Data Fig. 4:**
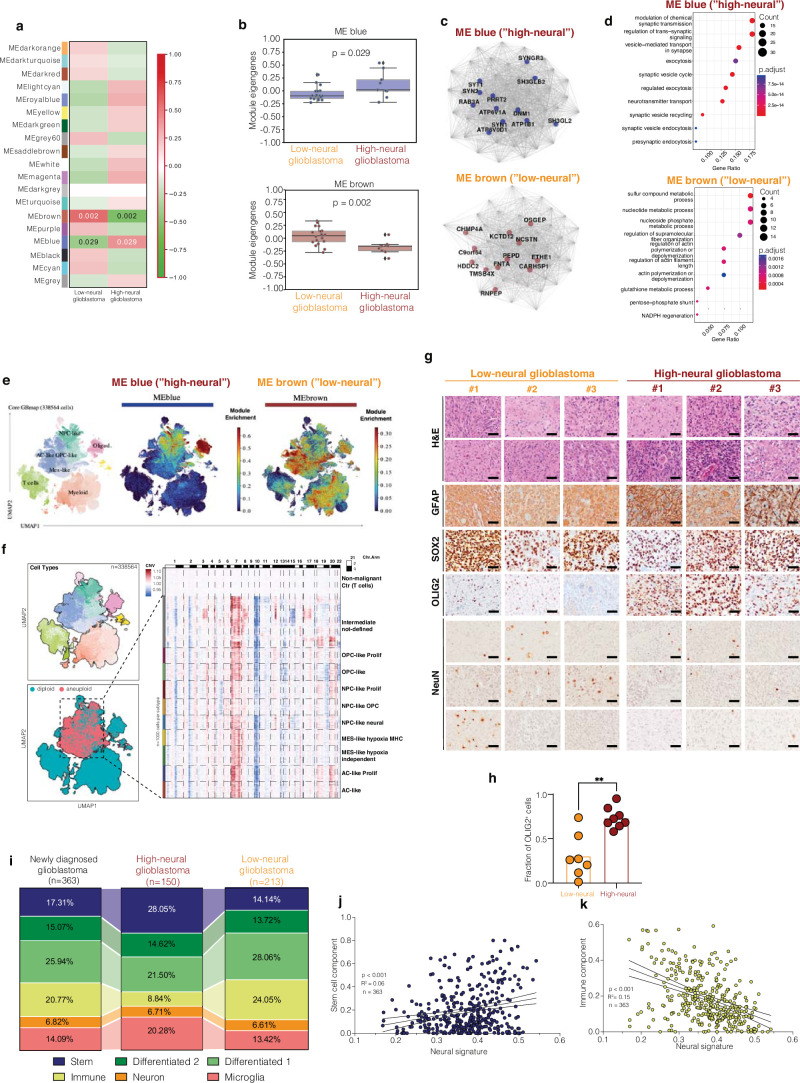
High-neural glioblastoma is linked with synapse formation and trans-synaptic signaling from proteomic profiling. **a** – **e) Proteomic profiling of low- (n=19) and high-neural (n=9) glioblastoma. a)**. WGCNA analysis showed differentially abundant proteome modules between both neural subgroups. **b)**. High-neural glioblastomas are clustered to module ‘blue’ (top figure), while low-neural glioblastomas have a higher abundance in module ‘brown’ (bottom figure). Data are mean ± s.e.m. Two-sided t-test P = 0.0.029 (top figure) and P = 0.002 (bottom figure). **c, d)**. Network analysis revealed **e)**. most expressed proteins and **f)**. associated gene ontology terms for each neural subgroup (high-neural: top, low-neural: bottom). **e)**. Integrating transcriptomic single-cell data showed an OPC-/NPC-like character in high-neural tumors (‘ME blue’). **f)**. Transcriptomic single-cell copy number variation plot analysis of glioblastomas with a high-neural signature. **g)**. Immunohistostaining of representative low- and high-neural glioblastoma samples. n=10 biological replicates. **h)**. Analysis of OLIG2^+^ cells between low- and high-neural glioblastoma samples. **P < 0.01, two-tailed Student’s t-test. **i)**. Comparison of abundance of cell states analyzed by reference-free deconvolution between newly diagnosed, high-neural, and low-neural glioblastomas. **j)**. Stem cell-like state significantly correlated with an increase of the neural signature in glioblastoma samples. Simple linear regression, P = 0.000003024480. Error bands representing the 95% confidence interval. **k)**. An anticorrelation was seen between the abundance of the immune compartment and the neural signature. Simple linear regression, P = 0.000000000005. Error bands representing the 95% confidence interval.

The synaptic character of high-neural glioblastoma was further validated in the tumor proteome (Extended Data Fig. 4a–f), showing an increase in proteins related to synaptic transmission (Extended Data Fig. 4a–d) and characteristics of malignant OPC-like, astrocyte-like and NPC-like cells (Extended Data Fig. 4e,f). Histopathological staining demonstrated a higher fraction of OLIG2-positive tumor cells in high-neural glioblastoma samples but comparable sparse infiltration of NeuN^+^ cells within the tumor samples (Extended Data Fig. 4g,h).

**Fig. 3:**
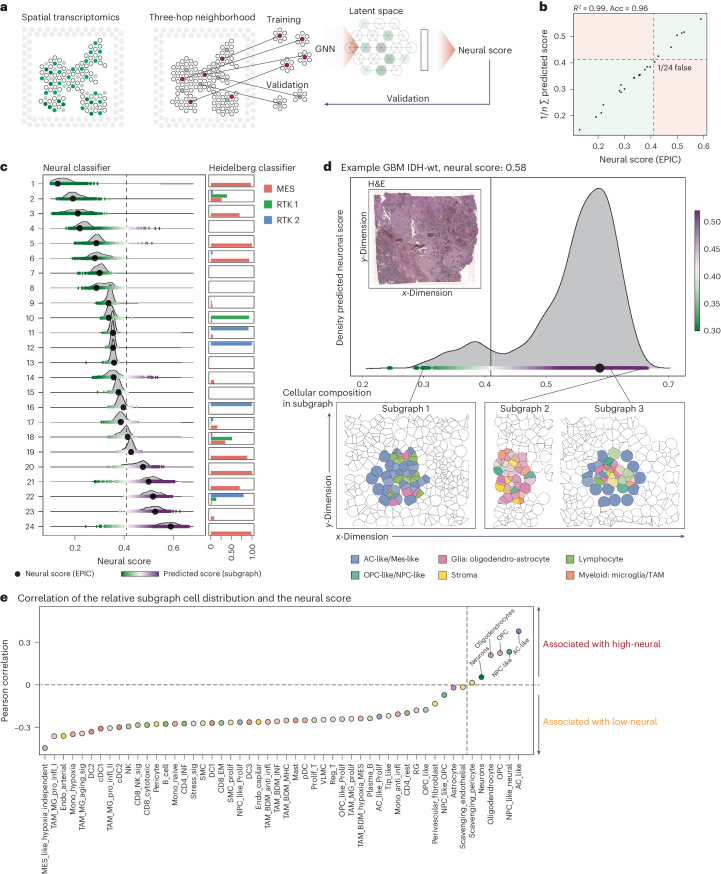
Spatially resolved architecture of low- and high-neural glioblastoma. **a**, Illustration of the workflow. Spatial transcriptomic data were used to identify neighborhoods defined as subgraphs. A GNN was trained to predict the neural score based on the spatial arrangements of transcripts. **b**, Scatter-plot of the mean sample predictions and the ground truth values. **c**, Illustration of the variance of neural score (predictions) compared to the threshold of 0.41. Bar plot indicates the Heidelberg classifier values of the glioblastoma subclasses (*n* = 24) (right). The dashed black line indicates the neural score threshold of 0.41. **d**, Example of a high-neural glioblastoma sample with a large blend of low- and high-neural predicted scores. The hematoxylin and eosin (H&E) image demonstrate the histology of the sample. Spatial neighborhoods derived from subgraphs with high- and low-neural scores are demonstrated (bottom). The single-cell maps are generated through single-cell deconvolution (Cell2Location) and CytoSpace spatial deconvolution. wt, wild type. **e**, Overview of the cell-type abundance correlated with the neural score.

Next, we leveraged spatially resolved transcriptomic data with paired methylation profiling (*n* = 24) to examine the molecular architecture and cell-type distribution in low- and high-neural glioblastoma samples (Fig. 3). We hypothesized that these tumors have distinct architectures, reflected by a unique spatial arrangement of transcripts that predict their epigenetic neural subgroup.

To this end, we trained a graph-neural network (GNN) using 1,000 randomly chosen microenvironments within the samples. Each microenvironment was centered on a 55-µm spot and extended up to 450 µm. These subgraphs were representative of the broader sample and were instrumental for the GNN training, achieving an *R*^2^ of 0.99 and an F1 score of 0.98, indicating that the neural score can be reliably predicted from the transcriptional landscape (Fig. 3a,b).

We applied our neural score threshold of 0.41 to categorize microenvironments as ‘neural high’ or ‘neural low’. Of note, 41.2% of the samples exhibited a blend of both categories, including those at the threshold and those with the most elevated neural scores (Fig. 3c). For instance, a sample with a neural score of 0.58 showed two prominent peaks at 0.38 and 0.58, suggesting a diverse microenvironmental composition (Fig. 3d); however, a pure or predominant neural type was present in all but one of the 24 samples (95.8%). Further analysis revealed that high-neural score microenvironments typically encompass NPC-like and astrocyte-like tumor cells (Fig. 3e), alongside a significant presence of oligodendrocytes and OPC-like cells, painting a picture of the tumor microenvironment’s unique architecture associated with the high-neural phenotype.

In conclusion, single-cell and spatially resolved transcriptomic analyses decipher that the neural signature in glioblastomas predominantly originates from cells of the neural lineage exhibiting an OPC/NPC/astrocyte-like phenotype and is characterized by a distinct tumor microenvironment.

### High-neural glioblastomas resemble a malignant stem cell-like state

Using a nonreference-based multi-dimensional single-cell deconvolution algorithm, we observed a higher stem/progenitor cell-like state but lower immune component in high-neural glioblastoma (28.05%) compared to all newly diagnosed glioblastoma (17.31%) and low-neural glioblastoma (14.14%) (Extended Data Fig. 4i). Both components were significantly correlated with the neural signature (Extended Data Fig. 4j,k).

**Extended Data Fig. 5:**
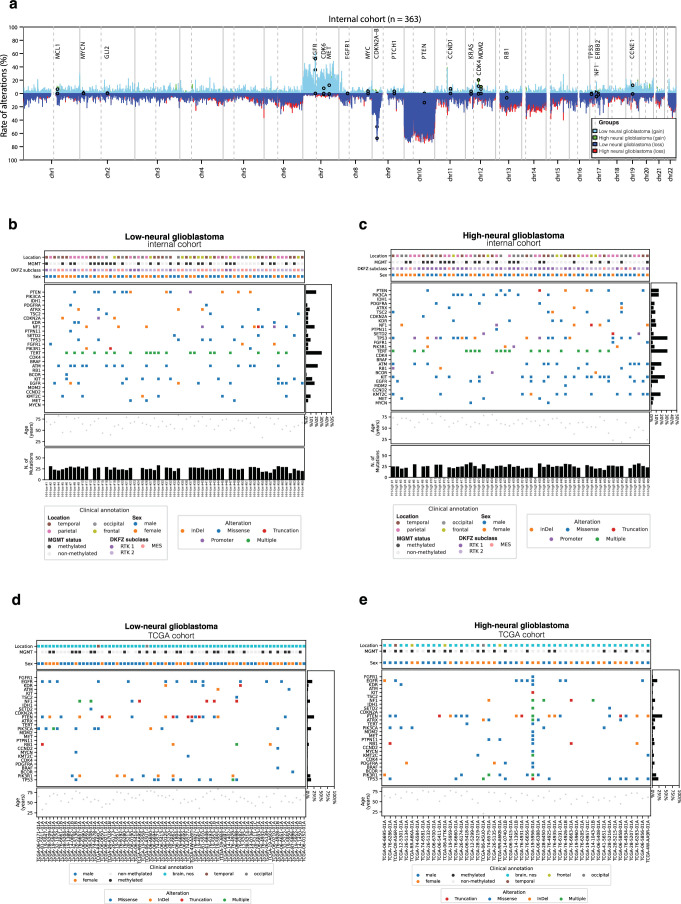
Copy number variations and next-generation sequencing of gene mutations between low- and high-neural glioblastoma samples. **a)**. Copy number variation plots for all samples stratified into low- and high-neural glioblastoma. **b, c)**. Oncoprint illustrating clinical characteristics and gene mutational status of **b)**. low-neural and **c)**. high-neural glioblastoma samples of our internal cohort. Of note, rarely detectable IDH mutations did not include the pathogenic R132H mutation. **d, e)**. Oncoprint illustrating clinical characteristics and gene mutational status of **d)**. low-neural and **e)**. high-neural glioblastoma samples of the TCGA dataset.

No significant copy-number variations were observed between low- and high-neural subgroups (conumee R package v.1.28.0)^26,27^ (Extended Data Fig. 5a). Next-generation sequencing (NGS) of 201 genes showed a higher frequency of *PIK3CA* (0 out of 65 (0.0%) versus 9 out of 60 (15.0%)) and *TP53* (6 out of 65 (9.23%) versus 19 out of 60 (31.67%)) mutations in high-neural tumors (Extended Data Fig. 5b,c). These findings were confirmed by an analysis of paired epigenetic and sequencing data of the TCGA dataset (Extended Data Fig. 5d,e).

### High-neural glioblastomas integrate into neuron-to-glioma networks

**Fig. 4:**
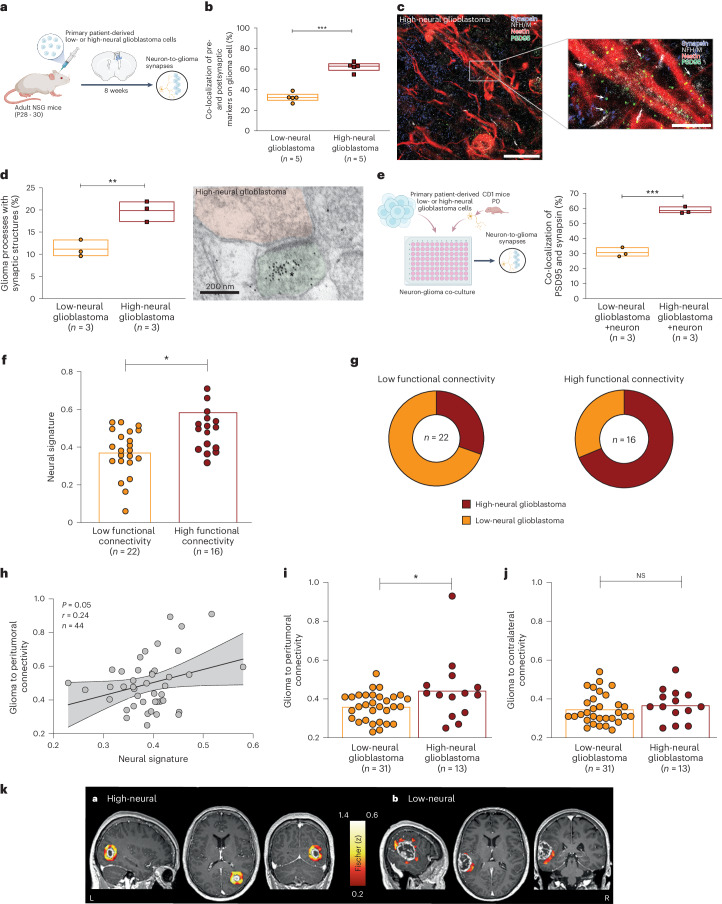
High-neural glioblastomas are integrated into neuron-to-glioma networks. **a**, Experimental workflow. **b**, Quantification of the colocalization of presynaptic and postsynaptic markers in low-neural (*n* = 22 regions, five mice) and high-neural (*n* = 21 regions, five mice) glioblastoma xenografts. *P* = 0.0008, two-tailed Student’s *t*-test. Data are mean ± s.e.m. **c**, Confocal image of infiltrated whiter matter of high-neural glioblastoma xenograft. White box and arrowheads highlight magnified view of synaptic puncta colocalization. Blue, synapsin-1 (presynaptic puncta); white, neurofilament heavy and medium (axon); red, nestin (glioma cell processes); green, PSD95 (postsynaptic puncta). Scale bars, 500 μm (top) and 250 μm (bottom). **d**, Electron microscopy of red fluorescent protein (RFP)-labeled glioblastoma cells. Quantification of neuron-to-glioma synaptic structures as a percentage of all visualized glioma cell processes (left) and image of neuron-to-glioma process in a high-neural glioblastoma xenograft (right). Asterix denotes immunogold particle labeling of RFP. Postsynaptic density in RFP^+^ tumor cell (green), synaptic cleft and vesicles in presynaptic neuron (red) identify synapses. **P < 0.01, two-tailed Student’s *t*-test. Scale bar, 200 nm. Data are mean ± s.e.m. *n* = 3 biological replicates. **e**, Colocalization of PSD95 and synapsin-1 in low- and high-neural glioblastoma cells in co-cultures with neurons. *P* = 0.0007, not significant (NS), *P* > 0.05, two-tailed Student’s *t*-test, *n* = 3 biological replicates. Data are mean ± s.e.m. **f**, Neural signature categorized into low functional connectivity (LFC) and high functional connectivity (HFC) as defined by magnetoencephalography. *P* = 0.0327, two-tailed Student’s *t-*test. **g**, Overlap between samples classified to the functional connectivity by Krishna et al.^12^ and the epigenetic-based neural classification of our study. **h**, Correlation of neural signature with degree of peritumoral connectivity as defined by resting-state functional magnetic resonance imaging (rs-fMRI). Simple linear regression *P* = 0.05, error bands representing the 95% CI. **i**, Peritumoral functional connectivity (defined by rs-fMRI) in low- and high-neural glioblastoma. *P* = 0.0416, two-sided Mann–Whitney *U*-test. **j**, Functional connectivity to the contralateral hemisphere (defined by rs-fMRI) in low- and high-neural glioblastoma groups. NS, *P* > 0.05, two-sided Mann–Whitney *U*-test. **k**, Examples showing the region of interest (ROI)-to-voxel functional connectivity of the contrast-enhancing area to its peritumoral surrounding. Peritumoral connectivity of a high-neural glioblastoma (0.457) and mean functional connectivity to its peritumoral area of 0.837 (left). By contrast, a low-neural glioblastoma (0.347) is shown with mean functional connectivity to its peritumoral area of 0.294 (right).

The transcriptional and proteomic analysis revealed an increased synaptogenic character in high-neural glioblastomas. This led us to explore their integration into neuron-to-glioma networks. After xenografting, an increased colocalization of neuron-to-glioma synapse puncta (*P* < 0.01; Fig. 4a–c) was observed in high-neural glioblastoma which was proven using electron microscopy (*P* = 0.008; Fig. 4d). An increase of colocalization of synapse puncta in high-neural glioblastoma cells after co-culturing with cortical neurons was found (*P* < 0.001; Fig. 4e).

**Extended Data Fig. 6:**
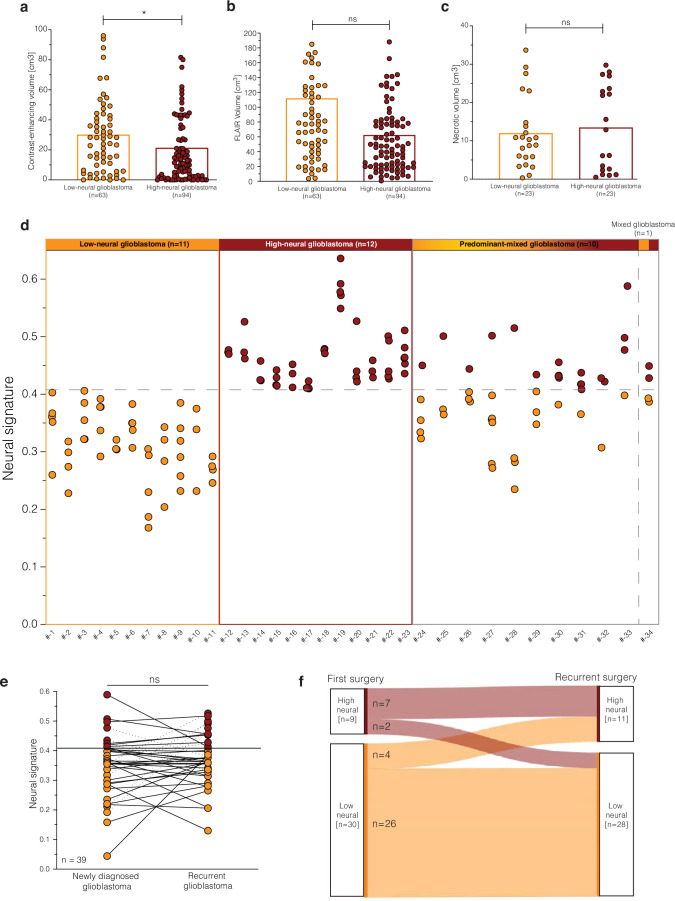
Radiographic parameters and spatiotemporal tumor sampling. **a – c)**. Association of neural glioblastoma group with volume of **a)**. contrast enhancement, **b)**. FLAIR, and **c)**. tumor necrosis measured by preoperative magnetic resonance imaging. A) *P* = 0.0374, b) *P* = 0.1767, and c) *P* = 0.6373, two-tailed Student’s t-test. **d)**. Analysis of intertumoral difference of neural signature within 34 newly diagnosed glioblastomas with spatial collection of 3 to 7 samples per tumor. 23 (67.6 %) of these tumors had a pure low- or high-neural signature in all individual biopsies with additional 10 (29.4 %) tumors being predominantly low or high. **e)**. Neural signature in 39 patients with matched tumor tissue obtained from surgery at first diagnosis and recurrence. ns: *P* > 0.05, two-tailed Student’s t-test. **f)**. Sankey plot illustrating a potential switch of the neural subgroup between first diagnosis and recurrence.

For clinical translation, we assessed functional tumor connectivity using magnetoencephalography (*n* = 38; Fig. 4f,g) and resting-state functional magnetic resonance imaging (*n* = 44; Fig. 4h–k) in patients with glioblastoma. Both modalities showed a significantly higher peritumoral connectivity within the high-neural subgroup (*P* < 0.01; Fig. 4f–i). This aligns with recent studies on cellular states in regions of HFC-glioblastoma^12^. Comparing the connectivity phenotype^12^ to our neural classification showed high concordance (Fig. 4g); however, no increased connectivity was seen between the tumor region and the contralateral hemisphere (Fig. 4j). Volumetric analysis showed significantly smaller volumes of contrast enhancement (*P* = 0.03; Extended Data Fig. 6a) in high-neural glioblastoma, but no association with fluid-attenuated inversion recovery (FLAIR) (*P* = 0.18; Extended Data Fig. 6b) and necrotic volume (*P* = 0.78; Extended Data Fig. 6c). These findings indicate that high-neural glioblastomas engender neuron-to-glioma synaptogenesis and have a distinct role within neuron-to-glioma networks exhibiting functional connectivity.

### Epigenetic neural signature is transferable to in vivo and in vitro models

**Fig. 5:**
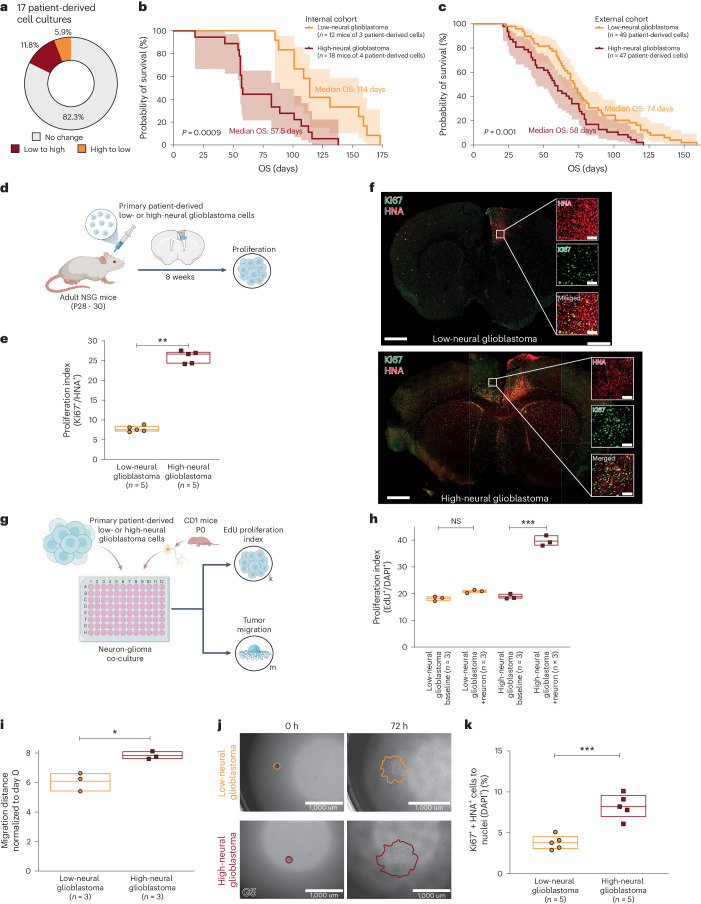
Neural classification is conserved in cell culture and correlates with survival as well as proliferation. **a**, Comparison of neural signature between patient’s tumor tissue and cell culture in 17 glioblastomas. **b**,**c**, Survival after xenografting of patient-derived low- and high-neural glioblastoma cells in our internal cohort (**b**) and two combined external cohorts (**c**). log-rank test, *P* = 0.0009 (**b**), *P* = 0.001 (**c**). Error bands represent 95% CI. **d**, Primary patient-derived low- and high-neural glioblastoma cell suspensions (*n* = 1 per group) were implanted into premotor cortex (M2) of adult NSG mice (*n* = 5 mice per group). Mice were perfused after 8 weeks of tumor growth and brains sectioned in the coronal plane for further immunofluorescence analyses. **e**, Proliferation index (measured by total number of HNA^+^ cells co-labeled with Ki67 divided by the total number of HNA^+^ tumor cells counted across all areas quantified) in low- and high-neural glioblastoma-bearing mice (*n* = 5 mice per group). *P* = 0.00819, two-tailed Student’s *t*-test. Data are mean ± s.e.m. **f**, Representative confocal images of proliferation index in low-neural (top) and high-neural glioblastoma (bottom) xenografts. Human nuclear antigen (HNA), red; Ki67, green. Scale bars, 1 μm (overview images) and 200 μm (magnified images). **g**, Experimental workflow. **h**, EdU proliferation index (measured by total number of DAPI^+^ cells co-labeled with EdU divided by the total number of DAPI^+^ tumor cells counted across all areas quantified) in low-neural (*P* = 0.418) and high-neural (*P* = 0.0000172) glioblastoma as monocultures and co-cultured with neurons. Two-tailed Student’s *t*-test, *n* = 3 biological replicates. Data are mean ± s.e.m. **i**,**j**, 3D migration assay analysis comparing distance of migration 72 h after seeding (**i**) and representative images at time 0 h (left) and 72 h (right) of low- and high-neural glioblastoma cells (**j**). *P* = 0.0115, two-tailed Student’s *t*-test, *n* = 3 biological replicates. Scale bars, 1 μm. Data are mean ± s.e.m. **k**, In vivo spread of tumor cells into corpus callosum in low- and high-neural glioblastoma. *P* < 0.0004, two-tailed Student’s *t*-test. Data are mean ± s.e.m. EdU, 5-ethynyl-2′-deoxyuridine; DAPI, 4,6-diamidino-2-phenylindole.

Most studies elucidating the biology of cancer neuroscience in high-grade glioma were performed in preclinical models. Therefore, we examined the translatability of our epigenetic neural signature in cell cultures and patient-derived xenograft (PDX) models. We observed a well-preserved neural signature in 82.3% of our cell cultures compared to the original tumor samples (Fig. 5a), confirming that our preclinical models sufficiently reflect the characteristics of the original tumor. Comparison of low- and high-neural glioblastoma in PDX models of an internal cohort (*n* = 30 mice of seven patient-derived glioblastoma cell cultures; Fig. 5b) and two publicly available cohorts^28,29^ (*n* = 96 patient-derived glioblastoma cell cultures; Fig. 5c) showed a significantly shorter survival of mice bearing high-neural tumors (internal cohort, *P* = 0.0009; external cohort, *P* = 0.001). Additionally, an increased proliferation index was seen in high-neural glioblastoma in vivo using immunodeficient mice (*P* < 0.01; Fig. 5d–f) as well as in co-cultures with cortical neurons (*P* < 0.001; Fig. 5g,h). In accordance with current literature describing neuronal activity-driven widespread infiltration of glioblastoma cells^14^, we observed a significantly wider migration of high-neural glioblastoma cells in vitro (*P* < 0.05; Fig. 5i,j) and in vivo (*P* < 0.001; Fig. 5k). These findings demonstrate the robustness of the epigenetic neural signature in vitro and in vivo and indicate higher proliferation when receiving neuronal input.

### Epigenetic neural classification remains spatiotemporally stable

As heterogeneity is a hallmark of glioblastoma, we investigated the spatiotemporal heterogeneity of the epigenetic neural signature. First, we analyzed 143 spatially collected biopsies from 34 patients (3–7 samples per patient). Among them, 23 patients (67.6%) demonstrated a pure low- or high-neural signature, while ten patients (29.4%) exhibited a predominant signature (Extended Data Fig. 6d). Temporal stability was assessed in 39 patients with matched tissue from both initial and recurrence surgery (Extended Data Fig. 6e). Here, 31 out of 39 patients (79.5%) remained in the same neural subgroup at recurrence (Extended Data Fig. 6f). Overall, the neural subgroup seemed to be spatiotemporally stable in contrast to transcriptional states that change in a larger proportion of patients^30,31^.

### Drug sensitivity analysis of neural glioblastoma cells

**Extended Data Fig. 7:**
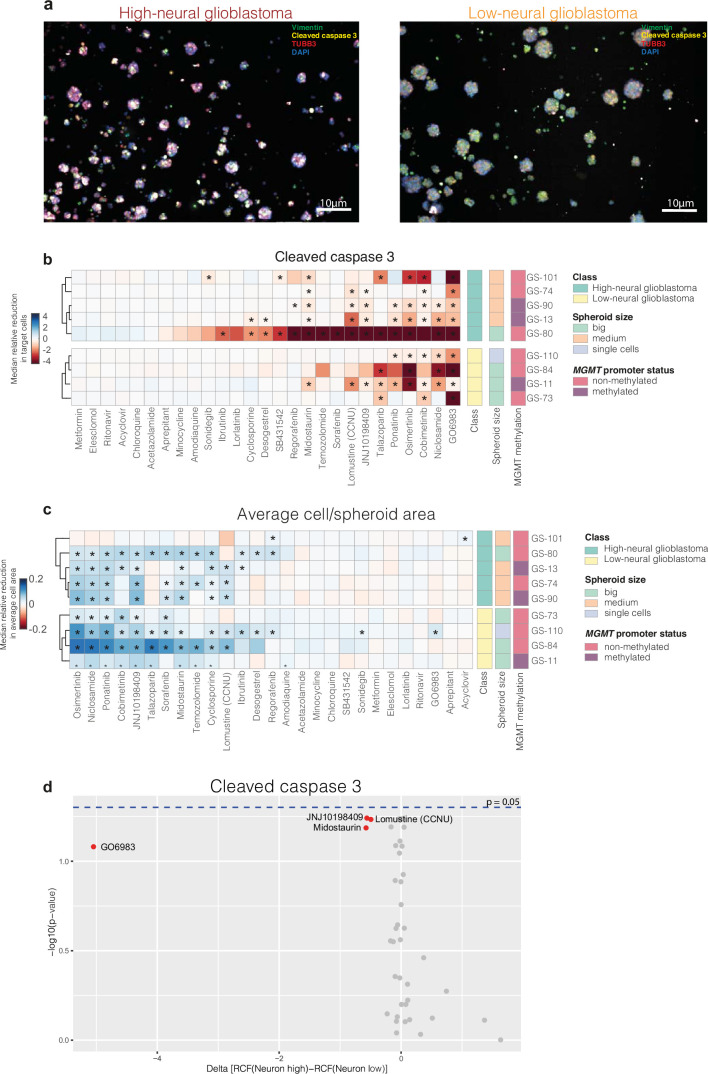
Drug sensitivity analysis of low- and high-neural glioblastoma cells. **a)**. Representative microscopic images for high- (left image) and low-neural (right image) glioblastoma cells. Green: Vimentin, yellow: cleaved caspase 3, TUBB3: red, DAPI: blue. Scale bars: 10μm. n=9 biological replicates. **b)**. Drug sensitivity of low- and high-neural glioblastoma cells measured by cleaved caspase 3. *P < 0.05, Mann–Whitney test. **c)**. Drug sensitivity of low- and high-neural glioblastoma cells measured by average cell area. *P < 0.05, Mann–Whitney test. **d)**. Statistical difference of sensitivity to various drugs between low- and high-neural glioblastoma cells. Mann–Whitney test.

Patients with glioblastoma routinely undergo combined radiochemotherapy after surgical resection^32^. We evaluated 27 different agents for their efficacy in the treatment of low- and high-neural glioblastoma cells (Extended Data Fig. 7a). We observed a trend for increased cleaved caspase 3 (Extended Data Fig. 7b) and reduced tumor cell size (Extended Data Fig. 7c) after treatment with lomustine (CCNU), JNJ10198400 and cyclosporine-treated high-neural glioblastoma cells, whereas talazoparib showed a trend for greater sensitivity in low-neural glioblastoma cells; however, none of these compounds reached statistical significance (Extended Data Fig. 7d). Therefore, we wondered about the prognostic impact of surgical resection as we previously demonstrated survival differences for other methylation-based glioblastoma subclasses^33^.

### Neural classification predicts benefit of resection

**Fig. 6:**
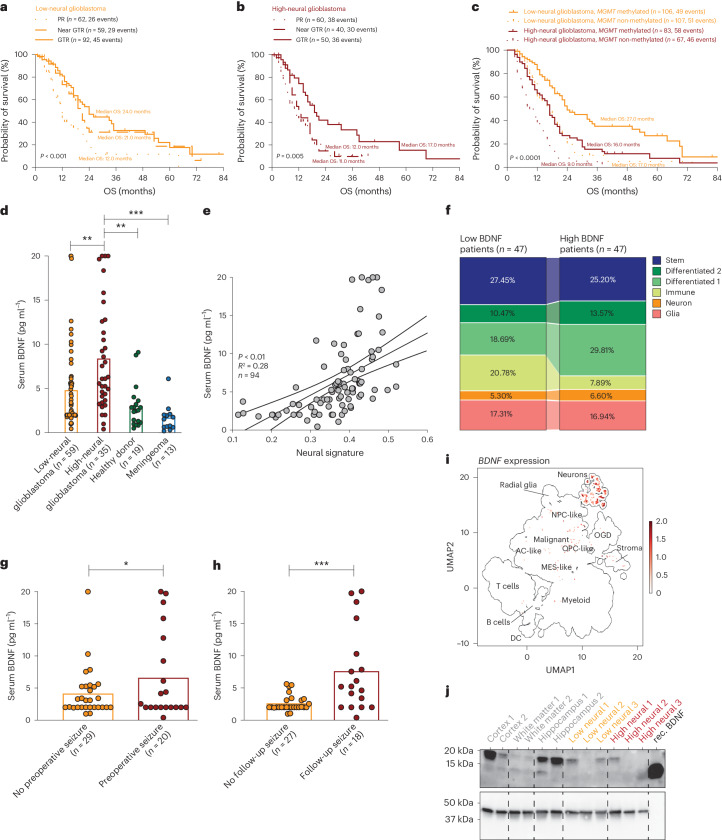
Neural classification predicts benefit of EOR and *MGMT* promoter methylation status and can be detected in serum of patients with glioblastoma. **a**,**b**, Survival outcome categorized after EOR in patients with glioblastoma treated by radiochemotherapy with a low-neural (**a**) and high-neural (**b**) tumor. log-rank test, *P* = 0.0003 (**a**), *P* = 0.005 (**b**). Error bands represent 95% CI. **c**, Survival outcome categorized by *MGMT* promoter methylation status in patients with glioblastoma treated by radiochemotherapy with a low- and high-neural tumor. log-rank test*, P* = 2.719 × 10^−11^. Error bands represent 95% CI. **d**,**e**, Immunoassay quantification of serum BDNF concentration of 94 patients with glioblastoma and healthy donors as well as patients with meningioma as control groups at the time of diagnosis. **P < 0.01, ***P < 0.001, two-tailed Student’s *t*-test; error bands represent 95% CI. **f**, Cell composition analysis in glioblastoma with low and high BDNF serum levels. **g**,**h**, Seizure outcome of patients with glioblastoma considering BDNF serum levels at the time of surgery (**g**) and during follow-up (**h**). *P < 0.05, ***P < 0.001, two-tailed Student’s *t*-test. **i**, Transcriptomic analysis of BDNF expression. **j**, Western blotting of BDNF in various healthy brain tissue samples and low- as well as high-neural glioblastoma. *n* = 3 biological replicates.

**Extended Data Fig. 8:**
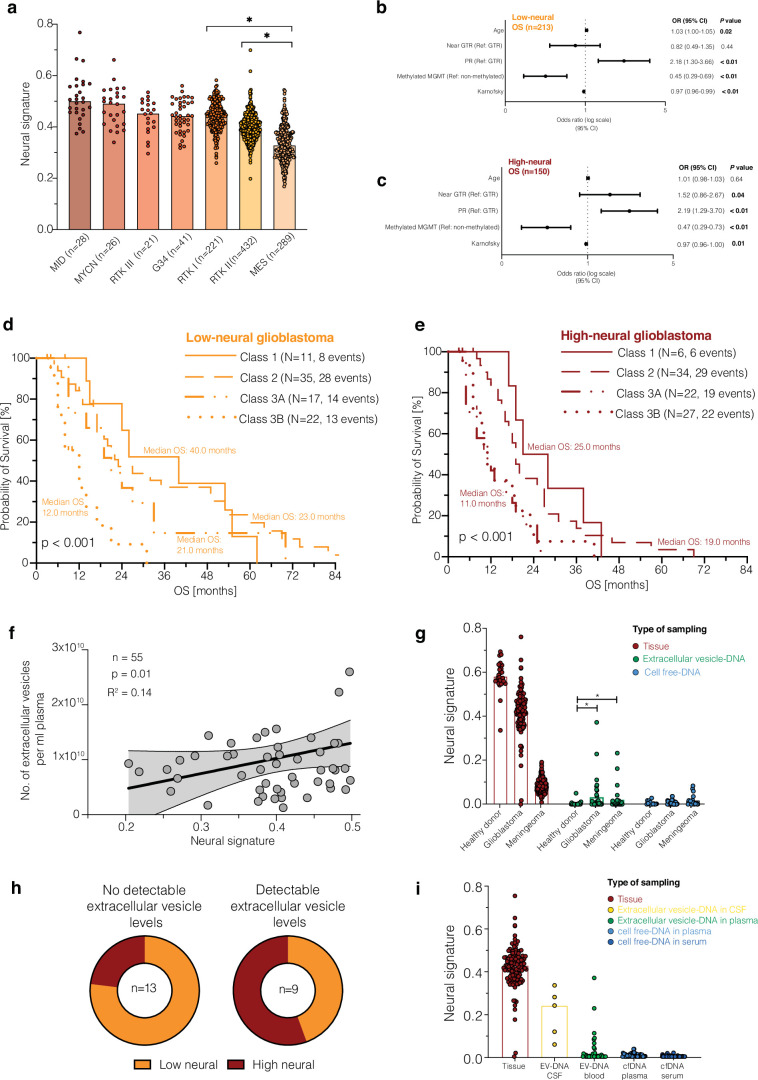
Clinical prognostic and circulating biomarkers of epigenetic neural glioblastomas. **a)**. Neural signature in DNA methylation subclasses of newly diagnosed IDH-wild-type glioblastoma. *P < 0.05, two-tailed Student’s t-test. **b)**. Forest plot illustrating the multivariate analysis of low-neural patients with glioblastoma. Means are shown by closed circles and whiskers representing the 95% confidence interval. Cox proportional hazards regression model. **c)**. Forest plot illustrating the multivariate analysis of high-neural patients with glioblastoma. Means are shown by closed circles and whiskers representing the 95% confidence interval. Cox proportional hazards regression model. **d – e)**. Survival outcome categorized after RANO categories for extent of resection in patients with glioblastoma treated by radiochemotherapy with a low- and high-neural signature. Class 1: 0 cm^3^ CE + ≤5 cm^3^ nCE tumor, Class 2: ≤1 cm^3^ CE, Class 3A: ≤5 cm^3^ CE, Class 3B: ≥5 cm^3^. Log-rank test, d) *P* = 0.0002, and e) P = 0.0011. **f.)** Correlation of neural signature and number of extracellular vesicles in patient serum at time of diagnosis. Simple linear regression P = 0.01. Error bands representing the 95% confidence interval. **g.)** Comparison of neural signature in healthy individuals, patients with glioblastoma, and meningeoma patients between matched tumor tissue, extracellular vesicle-associated DNA in serum, and cell-free DNA in serum. *P < 0.05, two-tailed Student’s *t*-test. **h.)** Comparison of patients with no detectable (left panel) and detectable (right panel) extracellular vesicle levels in serum stratified to their epigenetic neural glioblastoma type. **i.)** Illustration of the neural signature in different types of sampling in patients with glioblastoma.

Glioblastomas are epigenetically assigned to different subclasses^34^. Here, RTK I and RTK II (receptor tyrosine kinase I and II subtypes) tumors showed a comparable high-neural signature, whereas mesenchymal (MES) tumors had the lowest neural signature (Extended Data Fig. 7a). Given the different neural signatures between methylation-based subclasses, we hypothesized that the neural signature might constitute a factor for determining benefit from extent of resection (EOR). In low-neural glioblastoma, a significant survival benefit of gross total resection (GTR) (100% CE resection) and near GTR (≥90% CE resection) was observed compared to partial resection (<90% CE resection) (*P* < 0.001; Fig. 6a). By contrast, the survival benefit of a near GTR was not seen in high-neural glioblastoma (Fig. 6b). These findings held true in multivariate analyses (Extended Data Fig. 8b,c) and after applying the current criteria of the RANO (Response Assessment in Neuro-Oncology) resect group^35^ (Extended Data Fig. 8d,e). A methylated *MGMT* promoter showed a survival benefit in both neural subgroups, but a striking difference in low-neural glioblastoma with a median overall survival difference of 12.0 months depending on the *MGMT* promoter methylation status (*P* < 0.0001; Fig. 6c). Our combined survival data demonstrate that high-neural glioblastomas have an unfavorable outcome and a greater resection may be required to achieve a survival benefit in this distinct subclass.

### Serum biomarkers of neural glioblastoma

Next, we examined the feasibility of preoperatively determining the epigenetic neural subclassification in the blood of patients with glioblastoma to further reach clinical translation. By analyzing serum levels of brain-derived neurotrophic factor (BDNF) in 94 patients at diagnosis, we found higher BDNF levels in high-neural glioblastoma compared to low-neural glioblastoma, patients with meningioma (*n* = 13) and healthy individuals (*n* = 19) (Fig. 6d). The serum BDNF levels positively correlated with the epigenetic neural signature (*P* < 0.01, *R*^2^ = 0.28; Fig. 6e). Conversely, glioblastomas with higher BDNF serum levels had a decreased immune cell signature (Fig. 6f), consistent with the lower immune cell signature of high-neural tissue samples. We observed elevated BDNF levels in patients with glioma-associated seizures at the time of diagnosis (*P* = 0.02; Fig. 6g) and during follow-up (*P* < 0.001; Fig. 6h), which aligns with the known activity-regulated release of BDNF, most likely from healthy neurons (Fig. 6i,j) within high-neural glioblastoma networks.

Furthermore, we identified the neural signature in circulating extracellular vesicle-associated DNA (EV-DNA) and cell-free DNA (cfDNA) in patients’ plasma (Extended Data Fig. 8f–i). Circulating extracellular vesicles, a surrogate marker for glioblastoma^36,37^ and involved in neuronal synchronization^38^, correlated with the neural signature (Extended Data Fig. 8f). Epigenetic profiling of EV-DNA in plasma revealed a neural signature that was absent in cfDNA (Extended Data Fig. 8g). The neural signature detected in EV-DNA exhibited a significant increase in glioblastoma compared to samples from healthy donors and patients with meningioma (Extended Data Fig. 8g). Notably, high-neural tumors showed a higher incidence of a detectable neural signature in circulating EV-DNA (Extended Data Fig. 8h). While plasma-derived EV-DNA displayed markedly lower levels of neural signatures, cerebrospinal fluid EV-DNA exhibited lower but more comparable levels to tissue scores (Extended Data Fig. 8i).

Our findings suggest that BDNF could assist in stratifying patients with glioblastoma based on their neural subgroup, potentially facilitating targeted therapy in the future and that the neural signature is detectable in circulating extracellular vesicles.

### Epigenetic neural classification informs survival in diffuse midline glioma

**Extended Data Fig. 9:**
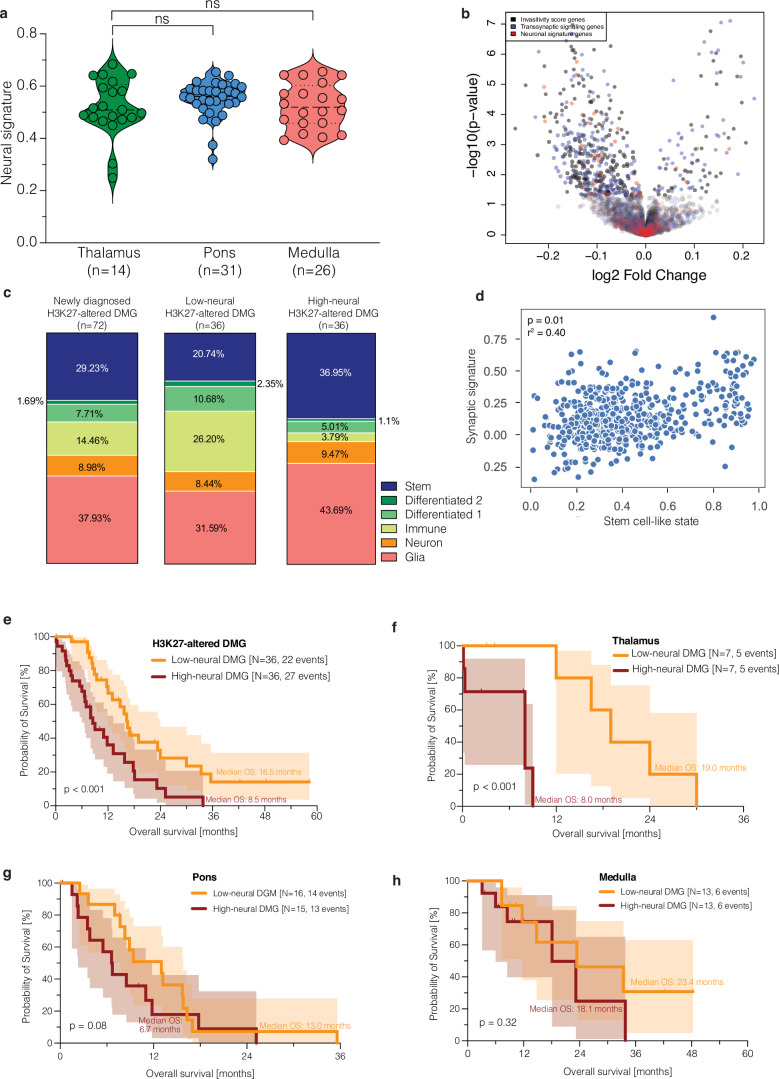
Relevance of neural classification in pediatric and adolescent patients diagnosed with *H3K27*-altered diffuse midline glioma (DMG). **a)**. Association of tumor location with neural signature. Two-tailed Student’s t-test. **b)**. Volcano plot showing differentially methylated CpG sites of genes of the invasivity signature, neuronal signature, and trans-synaptic signaling signature. **c)**. Cell state composition analysis in low- and high-neural DMG. **d)**. Synaptic gene expression (*PTPRS*, *ARHGEF2*, *GRIK2*, *DNM3*, *LRRTM2*, *GRIK5*, *NLGN4X*, *NRCAM*, *MAP2*, *INA*, *TMPRSS9*)^6^ is significantly correlated with the stem cell-like state of DMG cells calculated by an overlap of single-cell DNA methylation and single-cell RNA sequencing (599 cells from 3 study participants) measurements. Simple linear regression. **e – h)**. Kaplan–Meier survival analysis of 72 DMG patients under 18 years of age with a low- and high-neural DMG. Error bands representing the 95% confidence interval. Log-rank test, e) P = 0.0017, f) P = 0.0022, g) P = 0.0882, and h) P = 0.3236.

Besides glioblastoma, previous studies have highlighted the importance of neuronal activity-driven proliferation in DMG^6,7^. We identified the epigenetic neural signature in a cohort of H3 K27-altered DMG consisting of pediatric and adolescent patients from our institutional cohort (*n* = 21), Chen et al.^39^ (*n* = 24) and Sturm et al.^34^ (*n* = 10). The neural signature was evenly distributed among tumors in the thalamus, pons and medulla (Extended Data Fig. 9a). Similar to glioblastomas, areas in genes related to trans-synaptic signaling were mainly hypomethylated in high-neural DMGs (Extended Data Fig. 9b). A notable association with stem and glial cell states (Extended Data Fig. 9c) and increased synaptic gene expression^4^ (*P* = 0.01; Extended Data Fig. 9d) was observed in high-neural DMGs. Survival analysis of 72 patients showed an unfavorable outcome for high-neural DMG (*P* < 0.01; Extended Data Fig. 9e–g). These results confirm the relevance of the neural signature in an additional type of IDH-wild-type high-grade glioma.

## Discussion

In recent years, the bidirectional interaction between glioma cells and neural cells, with their ability to form synapses and integrate into neuronal networks, has been identified as a major factor in tumor progression^4,6,13,40^. In this study, we identified an epigenetically defined malignant neural signature as a potential marker for neural-to-glioma interactions and present the following findings: (1) A malignant neural signature is increased in glioblastoma and DMG, compared to nonmalignant brain tumors. (2) High-neural glioblastoma confers an unfavorable survival in humans and mice, and in addition, the neural signature is associated with higher functional connectivity in patients with glioblastoma. (3) High-neural glioblastoma shows an increased malignant stem cell and neural lineage character but decreased immune infiltration. (4) The neural signature remains robust in vitro and in vivo and high-neural glioblastoma-bearing mice show higher proliferation when receiving neuronal input as well as increased neuron-to-glioma synapse formation. (5) High-neural tumors benefit from a maximized resection. (6) Elevated BDNF serum levels are present in patients with high-neural glioblastoma. (7) The prognostic value can also be seen in H3K27-altered DMG.

Gliomas encompass a variety of cellular components of the tumor microenvironment and subgroups can be described according to distinct cellular states^15^. Epigenome profiling and deconvolution have been effective in characterizing these glioma subclasses^41,42^. A recent study highlighted the importance of epigenetic regulation across various cancer types and demonstrated a close epigenomic relationship between glioblastoma cells and OPCs^43^. Our determination of an epigenetic neural signature revealed an increase in glioblastoma and DMG, echoing findings of previous studies in preclinical models^4,7^. Nonetheless, it is essential to note that the neural signature was derived from a single cortical neuron reference generated from three IDAT files, and while we integrated DNA methylation data from healthy brain regions for comparison, a larger sample size might have provided clearer differentiation between low- and high-neural tumors.

High-neural glioblastoma showed gene upregulation and hypomethylation associated with invasiveness and neuro-glioma synapse formation. Glioma growth is known to involve paracrine signaling and glutamatergic synaptic input^4–8^, and recently a study subdivided glioblastoma cells into unconnected and connected cells with unique cell states, explaining brain infiltration through hijacking of neuronal mechanisms^13^. Our spatial transcriptomic analysis has unveiled the malignant stem-cell-like characteristics of high-neural glioblastoma, primarily clustering with cells of the neural lineage, such as OPC/NPC/astrocyte-like cells, alongside healthy oligodendrocytes and neurons. These findings align with the previously described unconnected glioblastoma cells that hijack neuronal mechanisms and drive brain invasion. While tumors with an OPC/NPC-like cellular state have been shown to overlap with the classical and proneural TCGA subtypes^15^, which have been assumed as having a better prognosis^25^, our identified high-neural glioblastoma demonstrated a poor patient outcome. This possible discrepancy may be explained by our integrated RNA-seq analysis, which revealed a wide heterogeneity of the transcriptomic TCGA subtypes in our epigenetic low- and high-neural tumors. In addition, this difference can largely be attributed to the noted transcriptional heterogeneity and plasticity within tumor populations^15,44^. Our study posits that the epigenetic signature offers a more stable marker than purely transcriptional profiles. Unlike the transient nature of transcriptional states, epigenetic signatures encompass not only the cells in OPC/NPC/astrocyte-like states but also reflect broader dependencies and interactions within the tumor microenvironment. Therefore, we argue that our high-neural phenotype should be interpreted as being driven by epigenetic factors that incline cells toward OPC/NPC/astrocyte-like states, rather than solely being a direct consequence of transcriptional variability.

Of note, the observed diploid oligodendrocyte transcriptomic module may represent a tumor cell population of primary near-diploid state as glioblastomas are karyotypically heterogeneous tumors^45–47^. Alternatively, it might be possible that surrounding healthy oligodendrocytes are affecting the neuronal activity-driven mechanisms on glioma cells^2^.

The clinical relevance of our findings is supported by the observation that patients suffering from high-neural glioblastoma or DMG had an unfavorable outcome. A greater EOR must be achieved to have prognostic improvement in high-neural glioblastoma, which may explain the results of our previous study examining the impact of DNA methylation subclasses^33^. Our findings are in line with a recent study by Krishna et al.^12^ demonstrating poorer survival in patients with glioblastoma exhibiting high functional connectivity. Integrating connectivity data from resting-state functional MRI and magnetoencephalography (MEG) linked an increased functional connectivity to its peritumoral surrounding with a higher neural signature in our patients. If a reliable stratification of the neural classification by MEG or MRI is predictable remains to be discussed in further studies. The synaptogenic character with increased connectivity of high-neural glioblastomas could be replicated with in vivo and in vitro experiments. Collectively, these data underscore the tremendous importance of the synaptic integration of gliomas into neuronal circuits and targeting these neuron-to-glioma networks seems to be a promising therapeutic approach^1,48^.

One factor drawing attention is BDNF, a neuronal activity-regulated neurotrophin, which has been found to promote glioma growth^6,49^ and interrupting BDNF–TrkB signaling has been shown to confer survival benefit in mice^5^. We found elevated serum BDNF levels in patients with high-neural glioblastoma and further correlation with increased seizure frequency. Potential sources of elevated BDNF include neurons in a glioma-induced state of hyperexcitability^4^, given the known activity regulation of BDNF secretion^50–52^ or possibly from glioblastoma cells^53^. In brief, neuronal activity arising from glioma-to-neuron interactions during tumor growth or seizure initiation seems to be a pivotal driver for BDNF release and identifies a potential biomarker of high-neural glioblastoma.

While the BDNF–TrkB axis may represent a therapeutic target for high-neural glioblastoma, we further identified low-neural tumors as immune-enriched based on transcriptomic and cell state composition analysis. Consequently, one could hypothesize that two opposing glioblastoma subtypes seem to be differentiated here and will need to be pursued in future studies and therapeutic avenues. The identification of an immunosuppressive state in high-neural glioblastoma is concordant with recent findings which described immunosuppressive mechanisms in thrombospondin-1-upregulated glioma samples^54^. This stratification of IDH-wild-type gliomas based on their epigenetic neural signature may provide a potential tool for predicting response to neuroscience-guided therapies.

## Conclusion

Overall, the definition of a high-neural signature in IDH-wild-type glioma revealed a malignant NPC/OPC/astrocyte-like character that affects patient survival, remains stable during therapy and is conserved in preclinical models. This knowledge supports clinicians in stratifying patients with glioma according to their prognosis and determining the surgical and neuro-oncological benefit for current standard of care. Last, the here-presented clinical translation in the field of glioma neuroscience using an epigenetic neural signature may advance the development of trials with neuroscience-guided therapies.

## Methods

### Patient cohorts

Several patient cohorts were analyzed based on the glioma subclass. A clinical cohort of 363 patients who underwent IDH-wild-type glioblastoma resection at University Medical Center Hamburg-Eppendorf, University Hospital Frankfurt or Charité University Hospital Berlin was analyzed. Informed written consent was obtained from all patients and experiments were approved by the medical ethics committee of the Hamburg chamber of physicians (PV4904). The TCGA-GBM cohort was included for external validation^19^. A clinical cohort of pediatric and adolescent patients who underwent surgery for H3K27-altered DMG at University Medical Center Hamburg-Eppendorf was established and extended with cohorts from Sturm et al.^34^ and Chen et al.^39^. The reference and diagnostic set (*n* = 3,905) from Capper et al.^18^ was utilized.

### Clinical definitions

Diagnosis for the clinical cohort followed World Health Organization (WHO) classification guidelines^55^. The EOR of contrast-enhancing parts was stratified into GTR, complete removal, near GTR, *>*90% removal and partial resection, *<*90% removal. Overall survival refers to diagnosis until death or last follow-up and PFS from diagnosis until progression according to RANO criteria based on local assessment^56^. Seizures and antiepileptic medication use were defined by the current International League Against Epilepsy guidelines^57^. T1-weighted and T2-weighted FLAIR MRI images were analyzed using the Brainlab program. The volume of contrast enhancement, FLAIR hyperintensity and necrotic volume was assessed in cm^3^ obtained via multiplanar 3D reconstruction of the tumor ROI, enabled by delineating with the tool ‘Smart Brush’ manually in every slice.

### Stereotactic biopsies for spatial sample collection

Biopsies were obtained using a cranial navigation system (Brainlab v.13.0) and intraoperative neuronavigation. To limit the influence of brain shift, biopsies were obtained before tumor removal at the beginning of surgery with minimal dural opening. Tissue samples were then transferred to 10% buffered formalin and sent to the Department of Neuropathology for further processing and histopathological evaluation.

### Measurement of functional connectivity using magnetoencephalography

Tumor tissues with HFC and LFC sampled during surgery based on preoperative MEG were obtained from patients with IDH-wild-type glioblastoma operated on in the Department of Neurosurgery, University of California, San Francisco^12^. From each formalin-fixed paraffin-embedded (FFPE) tissue block, four serial sections at a thickness of ~10 µm each were used for DNA extraction. DNA was extracted with the QIAamp DNA FFPE kit (QIAGEN). DNA was quantified using the Nanodrop Spectrophotometer (Thermo Scientific). The ratio of optical density at 260 nm to 280 nm was calculated and served as the criterion for DNA quality.

### Functional connectivity by rs-fMRI

Forty-four treatment-naive patients with glioblastoma (mean age 65 ± 9 years) underwent rs-fMRI before surgery, with tumor tissues subsequently analyzed for genome-wide DNA methylation patterns using the Illumina EPIC (850k) array. Functional data preprocessing followed a standardized protocol implemented in SPM12 (ref. ^58^) within MATLAB (v.9.5)^59,60^. In brief, functional images were realigned, unwarped and coregistered to the structural image. Segmentation, bias correction and spatial normalization were conducted, with functional images smoothed using a 5-mm FWHM Gaussian kernel. Further preprocessing steps included slice-time correction, regression of movement-related time series using ICA-AROMA^24^ and high-pass filtering (>0.01 Hz). Tumor lesions were segmented using ITK-SNAP^61^ software and utilized as regions of interest for seed-based correlation analysis to compute voxel-based tumor-to-peritumoral connectivity (Fisher z transformation). A 10-mm peritumoral distance mask was created, and mean functional connectivity between the tumor and its peritumoral surrounding was computed using a ROI-to-voxel approach.

### Immunoblotting

Frozen tissue samples were lysed using RIPA buffer, containing 50 mM Tris-HCl (pH 7.5), 150 mM NaCl, aprotinin (10 mg ml^−1^), 1 mM phenylmethylsulfonyl fluoride, leupeptin (10 mg ml^−1^), 2 mM Na_3_VO_4_, 4 mM EDTA, 10 mM NaF, 10 mM sodium pyrophosphate, 1% NP-40, 0.1% sodium deoxycholate and 1% protease inhibitor (Merck). Total protein concentration was measured by the bicinchoninic acid (BCA) assay (Pierce). Proteins were separated using Tris-glycine gels, blotted into nitrocellulose membrane and probed with antibodies anti-BDNF (1:1,000 dilution, Cell Signaling, 47808) and anti-β-actin (1:1,000 dilution, Sigma-Aldrich A2228).

### Immunohistochemistry

Tissue samples were fixed in 4% formaldehyde, dehydrated, embedded in paraffin and sectioned at 2 μm following standard laboratory protocols. Immunohistochemical staining for NeuN (Chemico, MAB377, 1:200 dilution), Sox2 (Abcam, AB79351, 1:200 dilution), OLIG2 (R&D Systems, AF2418, 1:50 dilution) and GFAP (DAKO, M0761, 1:200 dilution) was conducted using an automated staining machine (Ventana BenchMark TX, Roche Diagnostics). Detection was achieved using diamino-benzidine as a chromogen, with counter-staining performed using Mayer’s Solution (Sigma-Aldrich).

### Drug sensitivity analysis

Patient-derived glioblastoma cell lines (GS-11, GS-73, GS-84, GS-110, GS-13, GS-74, GS-80, GS-90 and GS-101) were dissociated into single cells and seeded into a 384-well plate at a density of 1,250–7,500 cells per well in neurobasal medium supplemented with B27, glutamine, pen/strep, heparin and human FGF and EGF. Cells were treated with 27 drugs and dimethylsulfoxide as a control in triplicate for 48 h at 37 °C and 5% CO_2_. After treatment, cells were fixed, blocked and stained with antibodies against vimentin, cleaved caspase 3 and TUBB3. Imaging was performed using an Opera Phenix automated confocal microscope and z-stacks were segmented based on DAPI staining using CellProfiler (v.2.2.0)^62^. Downstream analysis was conducted in MATLAB v.9.13.0, where marker-positive cells/spheroids were identified using linear thresholds. Cell counts and average cell/spheroid areas were averaged per condition and compared between drug treatment and control groups.

### Spatially resolved transcriptomics

#### Quality assessment RNA

RNA extraction from FFPE tissue sections was conducted following the ‘Purification of Total RNA from FFPE tissue sections’ protocol (July 2021 version). Two 10-µm sections per tissue block were processed and RNA was eluted using 14 µl RNase-free water. Subsequently, 2 µl of the eluted RNA was subjected to both the Qubit RNA High-Sensitivity Assay and the DNF-471 Standard Sensitivity RNA Protocol using the Fragment Analyzer, following the respective manufacturer’s instructions. RNA quality was assessed by computing the Distribution Value 200 (DV200) using Agilent’s ProSize Data Analysis Software. The DV200 represents the percentage of RNA fragments longer than 200 nucleotides within a range of 200–10,000 bp. A DV200 ≥ 50% is considered desirable according to 10x Genomics guidelines. Additionally, the software provided the RNA integrity number to supplement the quality assessment.

#### Tissue preprocessing

To prepare FFPE tissue for spatial transcriptomics, sections of 5-μm thickness were sliced using a microtome, floated in a 42 °C water bath and transferred onto glass slides. Following H&E staining, tissue examination under the EVOS microscope facilitated the selection of the area of interest. The ‘Visium Spatial Gene Expression for FFPE – Tissue Preparation Guide’ (CG000408, Rev A) guided the initial steps of tissue preprocessing. Modifications to these steps are detailed explicitly in subsequent descriptions. For hydration and trimming, without conducting a tissue adhesion test due to intact tissue adhesion on glass slides, FFPE tissue blocks underwent hydration in an ice water bath for 20 min, followed by trimming and cutting into 4-μm thick sections using the Thermo Fisher Scientific HM355 S automatic microtome. Trimming excess paraffin and tissue parts on a standard glass slide was performed, followed by floating the section in a 42 °C water bath for extension and smoothing. Sections were then fit onto Visium slides and dried using a thermocycler at 42 °C for 3 h, before being stored in a desiccator at room temperature overnight. After heating the Visium slides at 60 °C for 2 h, they underwent two 15-min immersions in xylene, followed by serial dilutions in 100%, 96%, 85% and 70% ethanol for 3 min each. The slides were finally rinsed in Milli-Q water for 20 s. The slides were stained with 1 ml hematoxylin for 3 min, washed in two successive Milli-Q water baths, treated with 1 ml bluing buffer for 1 min, washed again and then stained with 1 ml alcoholic eosin for 1 min, followed by another wash. Imaging was carried out with an EVOS M7000 microscope from Thermo Fisher Scientific at ×20 magnification in the brightfield setting, utilizing auto-focus for the first image of each capture area. Following imaging, the slide was placed into a Visium slide cassette (PN2000282) with an alignment tool (PN3000433). Pipetting was performed carefully to prevent disturbing the tissue, ensuring full coverage of the capture area and complete removal of leftover fluids. Each well of the cassette was treated twice with 100 μl 0.1 N HCl, then rinsed with 150 μl, pH 9.0 TE buffer, followed by another TE buffer application and incubation at 70 °C for 1 h on a thermal cycler. This initiated the library construction’s hybridization stage.

#### Library preparation

Fo the pre-hybridization mix application, each well received pre-hybridization mix, followed by a 30-min incubation at 37 °C. This was succeeded by an overnight incubation of probe hybridization mix at 50 °C, centrifugation, multiple washes and application of probe ligation mix for 1 h at 37 °C. Post-ligation wash buffer was applied, followed by several washes. For the RNase and permeabilization mix application, the RNase mix and permeabilization mix were each applied and incubated for 30 min and 1 h, respectively at 37 °C, followed by washing and probe extension mix application. For probe elution and PCR, 0.08 M KOH was utilized to elute the probe. After transferring the solution to an eight-tube-strip, 1 M, pH 7.0 Tris-HCl was added. Cycle numbers for PCR were determined using a qPCR mix and performed with a StepOnePlus Real-Time PCR System. Sample Index PCR followed, with cleanup using SPRIselect and transfer of 25 μl to a new tube strip. A second qPCR was performed with the NEBNext Library Quant kit for Illumina to determine library molarities, ensuring successful library construction and cDNA presence.

#### Sequencing

Sequencing of the libraries was conducted using the NextSeq 500/550 device from Illumina. Libraries were normalized to the same molarity before being combined. Denaturation and dilution of libraries were performed following the ‘NextSeq System – Denature and Dilute Libraries Guide’ protocol. The combined library was denatured with 0.2 N NaOH, neutralized and diluted to a loading concentration using High Output kits. PhiX control was denatured, diluted and mixed with the library. The final mix underwent sequencing with the NextSeq 500/550 High Output kit v.2.5 (75 cycles).

### Isolation and analysis of extracellular vesicles

Extracellular vesicles were isolated from plasma or cerebrospinal fluid of patients with glioblastoma by differential centrifugation^37,63^. After initial centrifugation steps to eliminate cells, platelets and large vesicles, extracellular vesicle pellets were obtained through ultracentrifugation. These pellets were resuspended with filtered PBS and analyzed for concentration and size using nanoparticle tracking analysis. Extracellular vesicle-enriched samples were diluted before nanoparticle tracking analysis and the analysis was conducted using appropriate parameters. Additionally, extracellular vesicles were characterized by electron microscopy for size and morphology and by imaging flow cytometry for extracellular vesicle markers (CD9, CD63 and CD81). DNA extraction from extracellular vesicles was performed using a purification kit. For comparison, bulk cfDNA was isolated from plasma using a commercial kit.

### Detection of BDNF serum levels

Plasma from patients with glioblastoma was isolated by double spin centrifugation of whole blood. Samples were aliquoted and stored at −80 °C before use. BDNF plasma levels were detected using the LEGENDplex Neuroinflammation Panel 1 (BioLegend). Data were acquired using the BD LSR Fortessa and Beckman Coulter Cytoflex LX flow cytometer and analyzed with the BioLegend LEGENDplex software.

### Proteomic processing of human glioblastoma samples

FFPE samples of tumors were obtained from tissue archives from the neuropathology unit in Hamburg. Tumor samples were fixed in 4% paraformaldehyde, dehydrated, embedded in paraffin and sectioned at 10 μm for microdissection using standard laboratory protocols. For paraffin removal, FFPE tissue sections were incubated in 0.5 ml n-heptane at room temperature for 30 min, using a ThermoMixer (ThermoMixer 5436, Eppendorf). Samples were centrifuged at 14,000*g* for 5 min and the supernatant was discarded. Samples were reconditioned with 70% ethanol and centrifuged at 14,000*g* for 5 min. The supernatant was discarded. The procedure was repeated twice. Pellets were dissolved in 150 µl 1% w/v sodium deoxycholate in 0.1 M triethylammonium bicarbonate buffer and incubated for 1 h at 95 °C for reverse formalin fixation. Samples were sonicated for 5 s at an energy of 25% to destroy interfering DNA. A BCA assay was performed (Pierce BCA Protein Assay kit, Thermo Scientific) to determine the protein concentration, following the manufacturer’s instructions. Tryptic digestion was performed for 20 μg protein, using the single-pot, solid-phase-enhanced sample preparation (SP3) protocol^64^. Eluted peptides were dried in a Savant SpeedVac Vacuum Concentrator (Thermo Fisher Scientific) and stored at −20 °C until further use. Directly before measurement, dried peptides were resolved in 0.1% formic acid to a final concentration of 1 μg μl^−1^. In total 1 μg was subjected to mass spectrometric analysis.

### Liquid chromatography–tandem mass spectrometer parameters

LC–MS/MS measurements were performed using a QExactive mass spectrometer (Thermo Fisher Scientific) coupled with a Dionex Ultimate 3000 UPLC system (Thermo Fisher Scientific). Tryptic peptides were injected via an autosampler, purified, and desalted using a reversed-phase trapping column (Acclaim PepMap 100 C18 trap) before separation on a reversed-phase column (Acclaim PepMap 100 C18). Trapping occurred for 5 min at a flow rate of 5 µl min^−1^, followed by separation using a linear gradient from 2% to 30% solvent B over 65 min at 0.3 µl min^−1^. Peptides were ionized using nano-electrospray ionization (nano-ESI) with a spray voltage of 1,800 V and analyzed in data-dependent acquisition mode. During MS1 scans, ions were accumulated for a maximum of 240 ms or until reaching a charge density of 1 × 10^6^ ions (AGC target), with mass analysis performed at a resolution of 70,000 at *m*/*z* = 200 over a mass range of 400–1,200 *m*/*z*. Peptides with charge states between 2+ and 5+ and intensities above 5,000 were isolated within a 2.0 *m*/*z* isolation window in top-speed mode for 3 s from each precursor scan and fragmented using higher energy collisional dissociation with a normalized collision energy of 25%. MS2 scanning, conducted using an orbitrap mass analyzer, had a starting mass of 100 *m*/*z* with a resolution of 17,500 at *m*/*z* = 200 and was accumulated for 50 ms or until reaching an AGC target of 1 × 10^5^. Peptides that were already fragmented were excluded for 20 s.

### NGS of low- and high-neural glioblastoma samples

Tumor mutational profiling was conducted at the Department of Neuropathology, University Hospital Heidelberg, using a custom CNS tumor-specific NGS gene panel (Agilent, SureSelect Custom Tier2, 1,235 Mb). Library preparation followed manufacturer recommendations with the SureSelect XT HS2 DNA kit (Agilent, 5191-5688). Prepared libraries were pooled and sequenced on the Illumina Novseq6000 platform (Novaseq v.1.5 200 cycles S1 Reagent kit, 20028318). The NGS panel covers the entire coding region, along with selected intronic and promoter regions of 201 genes relevant to CNS tumors. It detects single-nucleotide variants, small insertions/deletions (indels), exonic rearrangements and recurrent fusion events. Sequenced reads were mapped to GRCh38 using the nf-core/sarek (v.3.3.2) pipeline^65–67^, with single-nucleotide variant and structural variant calling performed using Strelka (v.4.4.0.0)^68^ and Manta (v.1.6.0)^23^. Variant annotation was performed using SNPeff (v.5.1d)^69^. Variants were filtered based on several criteria, including mapping to exonic regions, QUAL > 20, MQ > 30, DP > 15, high/moderate impact and a population frequency <0.001 from the 1000 Genomes project. Additionally, variants with high population frequencies in the Genome Aggregation Database (gnomAD), such as SETD2 c.5885C>T and KMT2C c.2447dupA, were filtered out.

### Mice housing

In vivo experiments were conducted following approved protocols from the Stanford University Institutional Animal Care and Use Committee and the University Medical Center Hamburg-Eppendorf, adhering to institutional guidelines and explicit permissions from local authorities. Animals were housed under standard conditions in pathogen-free environments, with temperature- and humidity-controlled housing and access to food and water in a 12-h light–dark cycle. For xenograft experiments, the Institutional Animal Care and Use Committee established guidelines based on indications of morbidity, with mice killed if they displayed signs of neurological morbidity or lost 15% or more of their body weight.

### Orthotopic xenografting of patient-derived low- and high-neural glioblastoma cells

NSG mice (NOD-SCID-IL2Rγ-chain-deficient, The Jackson Laboratory) were used for experiments conducted at Stanford University, with equal distribution of male and female mice. Primary patient-derived low- (‘UCSF-UKE-1’) or high-neural (‘UCSF-UKE-2’) glioblastoma neurospheres were prepared in sterile Hanks balanced salt solution (HBSS) and stereotactically implanted into the premotor cortex (M2) of mice at postnatal day (P) 28–30. Mice survival analyses were performed on NMRI-Foxn1nu immunodeficient mice (Janvier-Labs) at the University Medical Center Hamburg-Eppendorf. Neurospheres from cultured primary patient-derived low- (‘GS-8’, ‘GS-10’, ‘GS-73’ and ‘GS-80’) or high-neural (‘GS-57’, ‘GS-74’, ‘GS-75’ and ‘GS-101’) glioblastoma were injected into the striatum. External validation of mice survival data was conducted using publicly available datasets from Vaubel et al.^28^ and Golebiewska et al.^29^.

### Perfusion and immunofluorescence staining

Eight weeks post-xenografting, low and high-neural glioblastoma-bearing mice were anesthetized with intraperitoneal avertin and transcardially perfused with PBS followed by fixation in 4% paraformaldehyde (PFA) overnight at 4 °C. After cryoprotection in 30% sucrose for 48 h, brains were embedded in Tissue-Tek O.C.T. and sectioned coronally at 40 μm using a sliding microtome. For immunofluorescence, sections were blocked in a solution of 3% normal donkey serum and 0.3% Triton X-100 in TBS, followed by incubation with primary antibodies overnight at 4 °C. Antibodies used included mouse anti-human nuclei clone 235-1, rabbit anti-Ki67, rat anti-MBP, mouse anti-nestin, guinea pig anti-synapsin-1/2, chicken anti-neurofilament or anti-PSD95. After rinsing, sections were incubated with appropriate secondary antibodies and mounted with ProLong Gold Mounting medium.

### Confocal imaging and quantification of cell proliferation and infiltration

Cell quantification within xenografts was conducted by a blinded investigator using a Zeiss LSM980 scanning confocal microscope. A 1-in-6 series of coronal brain sections were selected, with four consecutive slices analyzed at approximately 1.1–0.86 mm anterior to bregma. HNA-positive tumor cells were quantified in each field to determine the proliferation index, calculated as the percentage of HNA-positive cells co-labeled with Ki67. Infiltration into the corpus callosum was assessed in the same sections, with HNA-positive tumor cells co-labeled with Ki67 and divided by the total number of DAPI-marked nuclei.

### Confocal puncta quantification

Images were captured using a ×63 oil-immersion objective on a Zeiss LSM980 confocal microscope. Colocalization analysis of synaptic puncta images from both low and high-neural glioblastoma xenograft samples was performed by a blinded investigator. A custom ImageJ processing script, developed at the Stanford Shriram Cell Science Imaging Facility, was utilized for this purpose. The script defined each pre- and postsynaptic puncta and assessed colocalization within a defined proximity of 1.5 μM. To subtract local background, the ImageJ rolling ball background subtraction method was applied. Peaks were identified using the imglib2 DogDetection plugin, which employs the difference of Gaussians to enhance the signal of interest. The plugin then assigned ROIs to each channel based on predefined parameters. Neuron and glioma ROIs were quantified, and the script extracted the number of glioma ROIs within 1.5 μm of the neuron ROIs. This script was implemented in Fiji/ImageJ using the ImgLib2 and ImageJ Ops libraries.

### Sample preparation and image acquisition for electron microscopy

Twelve weeks post-xenografting of low- (*n* = 3, ‘UCSF-UKE-1’) and high-neural glioblastoma cells (*n* = 3, ‘UCSF-UKE-2’), mice were killed via transcardial perfusion with Karnovsky’s fixative: 2% glutaraldehyde and 4% PFA in 0.1 M sodium cacodylate (pH 7.4). Transmission electron microscopy (TEM) analysis was conducted on tumor masses within the CA1 region of the hippocampus. Samples were post-fixed in 1% osmium tetroxide, washed and en bloc-stained overnight. Dehydration was performed using graded ethanol and acetonitrile. Samples were then infiltrated with EMbed-812 resin, followed by embedding in TAAB capsules and oven curing. Sections of 40–60 nm were cut on a Leica Ultracut S and mounted on 100-mesh Ni grids. For immunohistochemistry, grids underwent microetching with periodic acid and osmium elution with sodium metaperiodate. Grids were blocked, incubated with primary goat anti-RFP antibody overnight, rinsed and incubated with secondary antibodies. Grids were contrast stained with uranyl acetate and lead citrate. Imaging was conducted using a JEOL JEM-1400 TEM at 120 kV, with image capture facilitated by a Gatan Orius digital camera.

### Cell culture

Fresh glioblastoma samples were obtained from patients operated in the Department of Neurosurgery, University Medical Center Hamburg-Eppendorf. Samples were immediately placed in HBSS (Invitrogen), transferred to the laboratory and processed within 20 min. The tissue was cut into <1-mm^3^ fragments, washed with HBSS and digested with 1 mg ml^−1^ collagenase/dispase (Roche) for 30 min at 37 °C. Digested fragments were filtered using a 70-μm cell mesh (Sigma-Aldrich) and the cells were seeded into T25 flasks at 2,500–5,000 cells per cm^2^. The culture medium consisted of neurobasal medium (Invitrogen) with B27 supplement (20 μl ml^−1^, Invitrogen), Glutamax (10 μl ml^−1^, Invitrogen), fibroblast growth factor-2 (20 ng ml^−1^, Peprotech), epidermal growth factor (20 ng ml^−1^, Peprotech) and heparin (32 IE ml^−1^, Ratiopharm). Growth factors and heparin were renewed twice weekly. Spheres were split by mechanical dissociation when they reached a size of 200–500 μm. In this study, analyzed cell cultures with clinical data are represented in Extended Data Fig. 4. Long-term cultivation cell cultures were used from a publicly available dataset (*n* = 7, GSE181314) and one in-house cell line (*n* = 1).

### Neuron-glioma co-culture experiments

Neurons were isolated from CD1 (The Jackson Laboratory) mice at P0 using the Neural Tissue Dissociation kit - Postnatal Neurons (Miltenyi) and followed by the Neuron Isolation kit, Mouse (Miltenyi). After isolation, 150,000 neurons were plated onto glass coverslips (Electron Microscopy Services) after pre-treatment with poly-l-lysine (Sigma) and mouse laminin (Thermo Fisher)^4^. Neurons are cultured in BrainPhys neuronal medium (StemCell Technologies) containing B27 (Invitrogen), BDNF (10 ng ml^−1^, Shenandoah), GDNF (5 ng ml^−1^, Shenandoah), TRO19622 (5 μM; Tocris) and β-mercaptoethanol (Gibco). Half of the medium was replenished on days in vitro (DIV) 1 and 3. On DIV 5, half of the medium was replaced in the morning. In the afternoon, the medium was again replaced with half serum-free medium containing 75,000 cells from patient-derived low- (‘UCSF-UKE-1’) or high-neural (‘UCSF-UKE-2’) cell cultures. Cells were cultured with neurons for 72 h and then fixed with 4% PFA for 20 min at room temperature and stained for puncta quantification as described above.

### EdU proliferation assay

For EdU proliferation assays, coverslips were prepared as described above. Again, at DIV 5, low-neural (‘UCSF-UKE-1’) or high-neural (‘UCSF-UKE-2’) glioblastoma cells were added to the neuron cultures. Forty-eight hours after addition of glioblastoma cells, slides were treated with 10 μM EdU. Cells were fixed after an additional 24 h using 4% PFA and stained using the Click-iT EdU kit and protocol (Invitrogen). Proliferation index was then determined by quantifying the percentage of EdU-labeled glioblastoma cells (identified by EdU^+^/DAPI^+^) over total number of glioblastoma cells using confocal microscopy.

### 3D migration assay

3D migration experiments were performed as previously introduced^70^ with some modifications. In brief, 96-well flat-bottomed plates (Falcon) were coated with 2.5 μg per 50 μl laminin per well (Thermo Fisher) in sterile water. After coating, a total of 200 μl of culture medium per well was added to each well. A total of 100 μl of medium was taken from 96-well round-bottom ULA plates containing ~200-μm diameter neurospheres of low- (‘UCSF-UKE-1’) and high-neural (‘UCSF-UKE-2’) glioblastoma lines and the remaining medium, including neurospheres was transferred into the pre-coated plates. Images were then acquired using an EVOS M5000 microscope (Thermo Fisher Scientific) at time 0, 24, 48 and 72 h after encapsulation. Image analysis was performed using ImageJ by measuring the diameter of the invasive area. The extent of cell migration on the laminin was measured for six replicate wells normalized to the diameter of each spheroid at time zero and the data are presented as a mean ratio for three biological replicates.

### Bioinformatic and statistical analysis

#### DNA methylation profiling and processing

DNA was extracted from tumors, extracellular vesicles and bulk plasma, and analyzed for genome-wide DNA methylation patterns using the Illumina EPIC (850k) array. The processing of DNA methylation data was performed with custom approaches^71^. Methylation profiling results from the first surgery were submitted to the molecular neuropathology methylation classifier v.12.5 hosted by the German Cancer Research Center^18^. Patients were included if the calibrated score for the specific methylation class was >0.84 at the time of diagnosis^71^. For IDH-wild-type glioblastoma, patients (scores between 0.7 and 0.84) with a combined gain of chromosome 7 and loss of chromosome 10 or amplification of *EGFR* were included in accordance with cIMPACT-NOW criteria^72^. A class member score of ≥0.5 for one of the glioblastoma subclasses was required. Evaluation of the *MGMT* promoter methylation status was made from the classifier output v.12.5 using the *MGMT*-STP27 method^73^.

All IDAT files were processed using the preprocess Illumina (minfi, v.1.40.0)^74^. Probes with detection *P* values <0.01 were kept for further analysis. Probes with <3 beads in at least 5% of samples, all non-CpG probes, SNP-related probes and probes located on X and Y chromosomes were discarded.

#### Dichotomization of tumors into low- and high-neural subgroups

We used the cell-type-specific methylation signature available from Moss et al.^17^ consisting of 25 cell-type components. We used the original implementation of Moss et al. to perform cell-type deconvolution using non-negative least square linear regression.

We deciphered the neural signature in GBM using a combined dataset (*n* = 1,058) from Capper et al.^18^ (*n* = 624) and our institutional cohorts from Hamburg, Berlin and Frankfurt (all Germany) (*n* = 434). The combined dataset was dichotomized into low- (*n* = 529) and high-neural (*n* = 529) tumors using the median neural proportion of 0.41. This cutoff value was used to classify GBM into low- and high-neural tumors for all analyses. External validation was performed using the publicly available dataset from the TCGA-GBM database (*n* = 178)^19^.

#### Reproducibility of differential methylation sites between low- and high-neural groups

We performed differential methylation analysis of 363 samples of the internal cohort using dmpFinder function from minfi R package^74^ (v.1.40.0). In total, we identified 1,289 CpG sites differentiating low- and high-neural groups. To estimate the predictive power of these sites, we trained a logistic regression model using scikit-learn package (v.1.2.2) on the clinical cohort using the differentially methylated sites as input features. The model was subsequently applied to the other cohorts.

#### Cell state composition analysis

To infer cell-type and cell state abundance, we conducted a bulk DNA methylation assay using EPIC arrays and applied the reference-free deconvolution method by Silverbush et al.^75^. This method, trained on the DKFZ glioblastoma cohort and tested on TCGA-GBM data, successfully infers cell types (immune, glia and neuron) and malignant cell states (stem-like and differentiated). We followed the protocol of Silverbush et al.^75^, using the EpiDISH package^76^, utilizing the provided encoding and RPC method with 2,000 maximum iterations.

#### DNA tumor purity

Tumor purity was predicted in silico from DNA methylation data using the RF_purify Package in R^77^. This package uses the ‘absolute’ method, which measures the frequency of somatic mutations within the tumor sample and relates this to the entire DNA quantity^78^.

#### Integrative analysis of methylation and gene expression

WGCNA was performed using the hdWGCNA^22^ R package. Methylation-derived neural subgroup labels were considered as a trait. The optimal soft power was determined to be 16. For dimension reduction and visualization of the coexpression network, we employed the UMAP via the ModuleUMAPPlot function. Gene Ontology analysis was subsequently performed on the top 100 module-associated genes using the compareCluster function. Visualization of module-associated pathway activations was accomplished using the clusterProfiler package.

To contextualize the identified modules at a single-cell level, we utilized GBMap^23^ and the reference dataset of human motor cortex (Allen Institute). Both datasets were integrated by alignment of the latent space representation. Based on the zero-inflated nature of single-cell data, we estimated the module enrichment by the frequency of each gene (g) being detected and the expression values as follows:

$${m}_{\exp }=\frac{\mathop{\sum }\nolimits_{i=1}^{n}{x}_{n}\bullet n{\rm{\#}}i=1\left({x}_{n}=0\right)}{2n}$$

${m}_{\exp }$ refers to the module expression score per cell which is estimated by the mean of *x* the log normalized and scaled expression values of *n* genes from the WGCNA modules. The mean is normalized by the frequency of nonzero-determined genes.

#### SRT data analysis

Computational analysis of spatially resolved transcriptomics (SRT) data was performed by the SPATA2 R package (v.2.01). An SPATA object was prepared for the SRT data.

#### Single-cell deconvolution

Single-cell deconvolution was performed using Cell2location^79^ with the GBMap single-cell data^23^ as a reference. The SPATA object was converted into the AnnData format and mitochondrial genes were sequestered into the obsm[‘MT’] matrix of the object before training the model for 500 epochs on the GPU. After training, we invoked export_posterior on the model to extract the posterior distribution of cell-type abundances, drawing 1,000 samples to robustly estimate these abundances across spatial locations. The cell-type abundances were exported back to the SPATA object by the addFeature function of SPATA2.

#### RNA deconvolution

We utilized the GBMapExtended single-cell RNA-seq (scRNA-seq) dataset and the human neocortex dataset from the Allen Institute to perform cell-type deconvolution. Data preparation involved loading and transforming the scRNA-seq data into a SingleCellExperiment object with Seurat and SingleCellExperiment libraries in R, annotated with relevant cell and gene identifiers. We leveraged the digitalDLSorteR package to train a deconvolution model, initiating with the setting of a random seed for reproducibility, followed by loading scRNA-seq profiles into the digitalDLSorteR framework. Key parameters, including cell and gene identifiers and cell-type annotations, were specified. The digitalDLSorteR’s zinbwave parameters were estimated to simulate single-cell profiles, incorporating previous knowledge of cell-type distributions to refine the simulation. A bulk cell matrix was generated based on probabilistic design from simulated cell profiles, and a digitalDLSorter model was trained on this matrix with standard scaling. Post-training, the model was applied to deconvolve a dataset comprising RNA-seq and methylation data, processed to extract counts and metadata. The deconvolution results were then visualized using ggplot2, with sample types and percentage compositions graphed, showcasing the cellular heterogeneity across different samples.

#### Construction of spatial graphs from Visium SRTs

The SRT object was preprocessed with SPATA2, including log transformation of the count matrix and alignment of the imaging dataset (H&E Image). Nucleus positions were annotated using an automated ilastik pretrained segmentation algorithm. For samples with low image quality, we adapted CytoSpace^80^ in our workflow. Spot coordinates were extracted via the getCoordsDf function and a pairwise distance matrix was computed based on the ‘*x*’ and ‘*y*’ coordinates of cells. The zero values in the distance matrix were replaced with a constant value of 1,000 to avoid computational issues. This ensured that subsequent thresholding steps would not falsely consider a cell as its own neighbor. A distance threshold (one unit greater than the smallest nonzero distance) was employed to construct an adjacency matrix, where cells within the threshold distance were designated ‘1’ for adjacency and cells beyond the threshold were assigned ‘0’ for no adjacency. Unique cell barcodes were used to label the rows and columns of the adjacency matrix, obtained from getCoordsDf. The adjacency matrix was then transformed into an undirected graph using the graph_from_adjacency_matrix function from the igraph package. We obtained the gene expression matrix with 5,000 most variable genes from our object and transposed it to align with the graph’s vertices. Using the graphical representation, we characterized the local topology around a specific location, termed a ‘query spot,’ by identifying its *n*-hop neighborhood. Specifically, the three-hop neighborhood of a query spot was defined as the set of all spots reachable within three edges from the query spot in the graph.

#### GNN architecture

We used a deep neural network combining a graph isomorphism network (GIN) backbone with multiple multilayer perceptron (MLP) prediction heads. We used the Pytorch Geometric library and defined each spot as a node and edges were defined as the direct neighbors of each individual spot within a three-hop neighborhood. Node features were log-scaled and normalized expression values from the 5,000 most variably expressed genes. Non-expressed genes within a subgraph were masked. Edge features were defined based on each node’s direct neighbors, with each node having a maximum of six neighbors. Subgraphs with fewer than 15 nodes were excluded. Self-loop edges were added to input graphs before forward pass.

We employed a three-layer GIN, and in the *k*th graph convolutional layer to process batches (size of 32) of SRT data, messages were computed using MLPs,

$${m}_{{uv}}={\mathrm{MLP}}\left({h}_{u}\right)$$

where $u,v\in N\left(v\right)$ and then aggregated for each node *v* over neighborhood *N*(*v*),

$${a}_{v}=\sum _{u\in N\left(v\right)}{m}_{{uv}}$$

The updated embedding of node *v* was updated on the basis of all incoming messages to *v*,

$${h}_{v}^{{\prime} }={\mathrm{MLP}}\left({a}_{v}\right)$$

The GIN layers are represented as follows: *x*_v_ defines the expression vector of node v and *N*(v) is the set of its neighbors. The GIN convolution operation updates the feature vector of node v by aggregating features from *N*(v) and combining them with *x*_v_ own features. The updated feature vector ${x}_{v}^{{\prime} }$ is computed with ReLU (rectified linear unit) as follows:

$${x}_{v}^{\,{\prime} }={\mathrm{ReLU}}\left(\left(\left(1+\epsilon \right)\times {x}_{v}+\sum _{u\in N\left(v\right)}{\mathrm{ReL}}\left({x}_{u}\right)\right)\right)$$

we define $\epsilon$ as a learnable parameter that allows the model to weigh the importance of a node’s own features versus the features of its neighbors. This operation is stacked multiple times (*k* = 2) in the *k*th GIN to allow for deeper aggregation of neighborhood information. After each GIN convolutional layer, batch normalization and LeakyReLU activation with a negative slope of 0.2 are applied, followed by a dropout layer with a dropout rate of 0.5 for regularization. The latent space representation of the graph is obtained by passing the output of the second GIN convolutional layer through a linear transformation (self.merge) with weights initialized using the Xavier uniform method. The resulting features are merged into a latent space and then global mean pooling is applied to create graph-level representations.

For the prediction tasks, separate MLP modules are employed. Each MLP consists of a linear layer, a ReLU activation, batch normalization, dropout and a final linear layer that outputs the predictions. The MLPs are structured as follows:

$$h\left(x\right)={W}_{2}\times D\times B\times \phi \left({W}_{1}\times x+{b}_{1}\right)+{b}_{2}$$

Where *x* is the latent space vector to the MLP, *W*_1_ and *W*_2_ are the weight matrices for the first and second linear transformations, respectively, *b*_1_ and *b*_2_ are the bias vectors for the first and second linear transformations, respectively, *ϕ* denotes the ReLU activation function, applied element-wise, where ${\phi }_{z}={ma}\left(0,z\right)$, *B* represents the batch normalization operation applied to the activated output and *D* represents the dropout operation, which randomly zeroes some of the elements of its input with a certain probability to prevent overfitting.

For neural score prediction tasks, we minimized the squared L1 norm loss between predictions and score (torch.nn.L1loss).

#### Data split and evaluation metrics

We evaluated the GNN and comparative methods on both our proprietary Visium dataset and additional public domain datasets. We split the data into training and evaluation subsets using a stratified procedure. For the training dataset, we selected 20,000 subgraphs from spatial transcriptomics samples across 20 patients, incorporating clinical attributes such as tumor type and epigenetic neural score. For the evaluation dataset, we reserved samples from the remaining four patients, covering a range of neural scores. Additionally, we included a validation set of 24,000 subgraphs from all 24 patients, ensuring independence from the training set.

This approach ensured robust evaluation across diverse clinical and molecular features, with the neural score used as the prediction task, evaluated by *R*^2^ against the neural score from EPIC methylation profiling.

#### Evaluation of the subgraph cell composition

We commenced by retrieving the spatial coordinates of each nucleus using the getNucleusPosition function from the SPATAwrappers package. The spatial coordinates representing the nuclei positions were obtained as $P=\{\,{p}_{i}| i=1,\ldots ,N\}$ where *p*_*i*_ is the coordinate pair for the *i*th nucleus and *N* is the total number of nuclei. Spatial grid coordinates corresponding to the transcriptomics data points were retrieved, denoted as $G=\{\,{g}_{j}| j=1,\ldots ,M\}$, with each *g*_*j*_ representing the coordinate pair for the *j*th grid point. For each grid point *g*_*j*_, a vector of deconvolution scores ${D}_{j}=\{{d}_{{jk}}| k=1,\ldots ,T\;\}$ was extracted, where *d*_*jk*_ represents the score for the *k*th cell type at grid point *j* and *T* is the number of cell types. The scores were normalized to a range of [0, 1], and the number of cells of each type at each grid point was estimated as:

$${C}_{{jk}}={\mathrm{round}}\left(\frac{{d}_{{jk}}^{{\prime} }\times {N}_{j}}{\mathop{\sum }\nolimits_{k=1}^{T}{d}_{{jk}}^{{\prime} }}\right)$$

where ${d}_{{jk}}^{{\prime} }$ is the normalized score and *N*_*j*_ is the number of cells at grid point *j*. Cell types were assigned to each grid point *g*_*j*_ to create a mapping *M*_*j*_, correlating grid points with their respective cell types. The cell-type mapping was integrated with nucleus position data to produce a comprehensive spatial map of cell-type distribution: $S=\{\left({p}_{i},{M}_{j}\right)| {p}_{i}\in P,{M}_{j}\in M\}$. This methodology facilitates the visualization and analysis of the cellular composition within the tissue section, providing insights into the complex spatial organization of the cellular environment.

#### Proteomic data processing

Proteomic samples (*n* = 28) were measured with liquid chromatography–tandem mass spectrometry (LC–MS/MS) systems and processed with Proteome Discoverer v.3.0. and searched against a reviewed FASTA database (UniProtKB^81^: Swiss-Prot, *Homo* *sapiens*, February 2022, 20,300 entries). The protein abundances were normalized at the peptide level. Perseus v.2.0.3 was used to obtain log_2-_transformed intensities. The imputation was performed using the random forest imputation algorithm (hyperparameters, 1,000 trees and ten repetitions) in RStudio v.4.3.

#### WGCNA for proteomics

We used hdWGCNA^82^ to identify gene coexpression modules, employing a soft power of 9 and minimum module size of 10. After correcting for technical batch effects, significant modules (*P* < 0.05) were selected based on their correlation with traits. Overrepresentation analysis of gene sets within these modules was performed using clusterProfiler^67^. Cell-type enrichment within modules was identified using gene sets from PanglaoDB through the Python package enrichr^68^. Module scores on single cells were calculated using Scanpy’s score_genes function with the core GBM single-cell atlas (GBMap)^23^.

#### Electron microscopy data analysis

Sections from xenografted hippocampi of mice were imaged using TEM imaging. The xenografts were originally generated for a study by Krishna et al.^12^ and mouse tissue was re-analyzed after epigenetic profiling and assignment to low- or high-neural glioblastoma groups. Here, 42 sections of high-neural glioblastoma across three mice and 45 sections of low-neural glioblastoma across three mice were analyzed. Electron microscopy images were taken at ×6,000 with a field of view of 15.75 μm^2^. Glioma cells were counted and analyzed after identification of immunogold particle labeling with three or more particles. Furthermore, to determine synaptic structures all three of the following criteria had to be clearly met as previously described^4^: (1) presence of synaptic vesicle clusters; (2) visually apparent synaptic cleft; and (3) identification of postsynaptic density in the glioma cell. To quantify the percentage of glioma cells forming synaptic structures, the number of glioma-to-neuron synapses identified was divided by the total number of glioma cells analyzed.

#### Statistical analysis

Gaussian distribution was confirmed using the Shapiro–Wilk test. Parametric data were analyzed with an unpaired two-tailed Student’s *t*-tests or one-way ANOVA with Tukey’s post hoc tests. Survival curves were generated using the Kaplan–Meier method, with statistical significance determined by two-tailed log-rank analyses. Multivariate analysis for overall survival and PFS included computing hazard ratios and 95% confidence intervals using Cox proportional hazards regression models. Variables with *P* < 0.05 in univariate analysis were included. Significance was set at *P* < 0.05. GraphPad Prism v.10 was used for statistical analyses and data illustrations and R Studio was used for alluvial plots.

### Reporting summary

Further information on research design is available in the Nature Portfolio Reporting Summary linked to this article.

## Online content

Any methods, additional references, Nature Portfolio reporting summaries, source data, extended data, supplementary information, acknowledgements, peer review information; details of author contributions and competing interests; and statements of data and code availability are available at 10.1038/s41591-024-02969-w.

### Supplementary information

Supplementary Information Gene sets and cell lines.

Reporting Summary

### Source data

Source Data Fig. 1 BDNF western blot.

Source Data Fig. 2 GAPDH western blot.

## Figures and Tables

**Extended Data Table 1:**
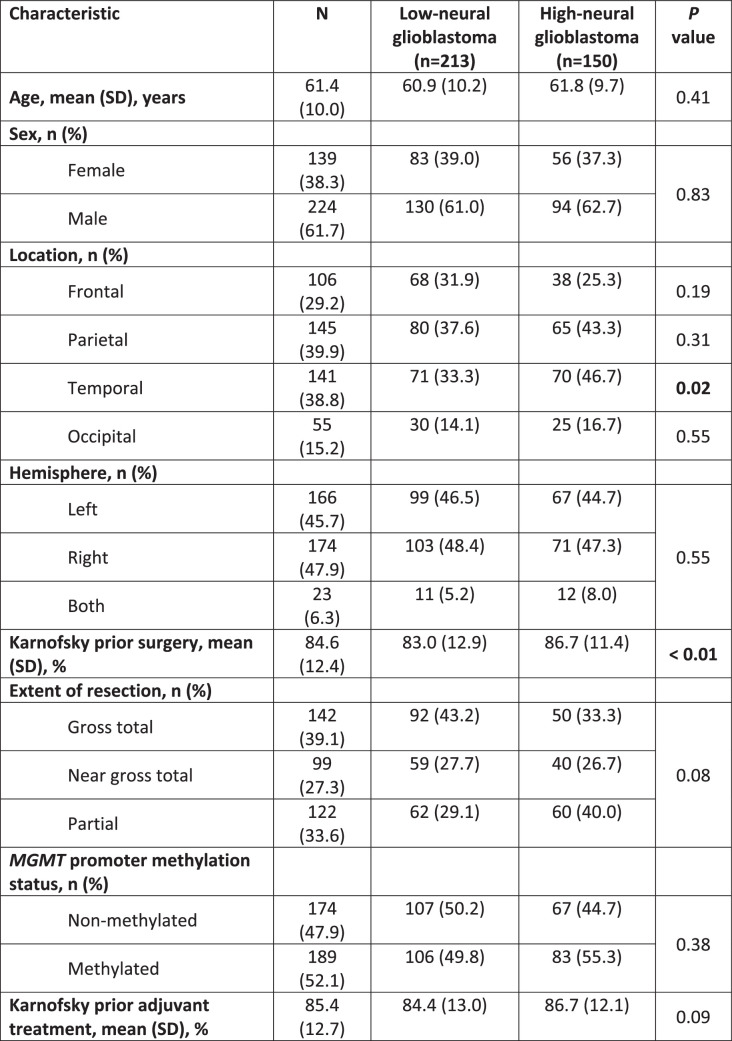
Clinical Characteristics

Clinical characteristics of patients with glioblastoma who were treated with combined radio chemotherapy after surgical resection. SD: standard deviation, MGMT: O6-methylguanine-DNA-methyltransferase.

## Data Availability

IDAT files of the clinical cohort (363 patients with GBM) are available at the Gene Expression Omnibus under accession code GSE240704. The methylation data provided by Capper et al.^18^ as illustrated in Extended Data Fig. 1 are accessible under accession code GSE109381. The TCGA-GBM cohort analyzed for external validation and as shown in Fig. 1d is accessible at https://portal.gdc.cancer.gov/projects/TCGA-GBM. Data files used in the spatial transcriptomic analyses are accessible at *Zenodo* at 10.5281/zenodo.10863736 (ref. ^83^). The single-cell RNA-seq dataset GBMap is available from the original publication and can be accessed through cellXgene (https://cellxgene.cziscience.com/collections/999f2a15-3d7e-440b-96ae-2c806799c08c) and the human motor cortex single-cell RNA-seq dataset is available from the Allen Brain Institute at https://portal.brain-map.org/atlases-and-data/rnaseq/human-m1-10x. Source data are provided with this paper.

## Code availability

The code used to perform DNA methylation and proteomics analysis is available at https://github.com/imsb-uke/epigenetic-neural-glioblastoma. Codes used for performing transcriptomic analyses in Figs. 2 and 3 and Extended Data Figs. 3 and 4f are available at https://github.com/heilandd/GBNeural. Additionally, the code for the non-reference-based multi-dimensional single-cell deconvolution from DNA methylation data as presented in Fig. 6f and Supplementary Fig. 4i can be found at https://github.com/danasilv/Deconvolution_of_GBM_bulk_DNA_methylation_profiles.
