# Supplementary material for: A prognostic neural epigenetic signature in high-grade glioma
Source: Nat Med. 2024 May 17;30(6):1622–35. doi: 10.1038/s41591-024-02969-w (PMC11186787; doi:10.1038/s41591-024-02969-w)
Supplement: Supplementary file 1 — Gene sets and cell lines. [file 41591_2024_2969_MOESM1_ESM.pdf]

nature medicine

Article https://doi.org/10.1038/s41591-024-02969-w

A prognostic neural epigenetic signature in high-grade glioma

In the format provided by the authors and unedited

Invasivity signature refering to Fig. 2a, modified from Venkataramani et al. (C		
**Gene with CpG site**	**FDC in high-neural glioblastoma**	**p-value**
ACAT2 cg01552187	-0.002831499	0.505450424
ACAT2 cg05879334	-0.00716411	0.240693002
ACAT2 cg06131755	0.009578746	0.878246513
ACAT2 cg09900253	-0.031348605	0.047378021
ACAT2 cg10268548	-0.089339749	0.000186993
ACAT2 cg10432632	-0.005114947	0.327391606
ACAT2 cg11366901	-0.000844221	0.890444426
ACAT2 cg13804918	-0.001767365	0.655053412
ACAT2 cg14767165	-0.022957351	0.017055807
ACAT2 cg15298323	0.007980776	0.892321385
ACAT2 cg18959411	0.007946555	0.434174425
ACAT2 cg23829577	-0.006605033	0.272794245
ACAT2 cg24224803	-0.010696249	0.089468298
ACAT2 cg24683514	-0.005742684	0.563348584
ACTB cg00804485	-0.001459163	0.758845435
ACTB cg01741041	-0.041150009	0.110366757
ACTB cg02356111	0.000586255	0.83973706
ACTB cg03324421	0.002539935	0.24438669
ACTB cg04490349	0.087479008	3.76202E-09
ACTB cg06003197	0.001098783	0.84901267
ACTB cg07476653	0.003021393	0.790127206
ACTB cg09041756	-0.001080041	0.913240466
ACTB cg13677897	-0.003932465	0.20803228
ACTB cg14145074	-0.029789492	0.211481959
ACTB cg15356195	6.33335E-05	0.98743804
ACTB cg18080670	0.00221091	0.597194605
ACTB cg23162587	0.000156949	0.983763106
ACTB cg23175281	-0.00231874	0.240008907
ACTB cg23261233	-0.000951931	0.826811142
ANAPC11 cg00014986	0.00266718	0.889811954
ANAPC11 cg01515426	-0.002872009	0.381683056
ANAPC11 cg02132003	-0.002832782	0.484925874
ANAPC11 cg02387854	-0.003452136	0.490020313
ANAPC11 cg05147328	-0.005058256	0.378086946
ANAPC11 cg08184047	-0.009016522	0.071462883
ANAPC11 cg08217660	-0.000573827	0.909824095
ANAPC11 cg10468580	-0.016037495	0.355238891
ANAPC11 cg12439463	-0.003445827	0.575885197
ANAPC11 cg13780054	-0.00144562	0.747206976
ANAPC11 cg14209926	-0.003639239	0.636761606
ANAPC11 cg18375296	-0.002544165	0.574105223
ANAPC11 cg21639988	-0.002891732	0.447969291
ANAPC11 cg21823502	0.109799697	0.007481461

ANAPC11 cg23402661	-0.00486093	0.430114732
ANAPC11 cg23639857	-0.002971663	0.646986047
ANAPC11 cg25245636	-0.002138617	0.611796194
ANXA2 cg00155593	-0.00208531	0.821163128
ANXA2 cg00930615	0.015082206	0.661056585
ANXA2 cg02072495	0.224340512	9.67929E-10
ANXA2 cg03957109	-0.03992108	0.088203536
ANXA2 cg06738887	0.110049076	8.55297E-05
ANXA2 cg08081036	-0.004200192	0.228862944
ANXA2 cg08163714	-0.023696827	0.159257088
ANXA2 cg09419486	-0.000414986	0.972523294
ANXA2 cg09533293	0.075811557	0.000125432
ANXA2 cg11421255	0.003164667	0.927987565
ANXA2 cg11681321	0.068389148	0.093502285
ANXA2 cg13313836	0.112597111	4.6538E-08
ANXA2 cg13634501	0.002205587	0.673941038
ANXA2 cg13785221	0.00188554	0.66595642
ANXA2 cg14397690	0.140771728	3.24978E-05
ANXA2 cg16472060	0.00152041	0.761721887
ANXA2 cg17893775	0.007862617	0.413031085
ANXA2 cg20118643	-0.010147926	0.791900126
ANXA2 cg21711008	0.000756063	0.978783908
ANXA2 cg22293140	0.013520142	0.40023998
ANXA2 cg22365276	0.027893444	0.344574201
ANXA2 cg22502206	-0.001510245	0.971858078
ANXA2 cg22581200	-0.077056656	5.65177E-05
ANXA2 cg25515997	0.040723081	0.013656094
ANXA2 cg27554954	0.00049047	0.986226239
AP2S1 cg00388479	-0.00091617	0.829704065
AP2S1 cg01358551	-0.000887304	0.954871857
AP2S1 cg02303361	0.000434366	0.893302566
AP2S1 cg06591892	0.002876302	0.682851005
AP2S1 cg07661340	0.000688833	0.967328744
AP2S1 cg10672726	-0.005841973	0.889713892
AP2S1 cg12258824	-0.005985545	0.902466201
AP2S1 cg13321746	0.000185525	0.971562253
AP2S1 cg14008648	-0.00123259	0.71260334
AP2S1 cg14815743	-0.023997915	0.258047761
AP2S1 cg23131950	-0.065172502	0.105822528
AP2S1 cg25230305	-0.00153449	0.936936157
ASCL1 cg00799539	-0.033931824	0.184798231
ASCL1 cg02246645	-0.000571143	0.918922054
ASCL1 cg03700449	0.001578118	0.850033386
ASCL1 cg04610718	-0.009202086	0.758023488
ASCL1 cg08927739	0.000596862	0.964141374
ASCL1 cg10672201	-0.017506898	0.496274039
ASCL1 cg10700564	-0.010173686	0.001265123
ASCL1 cg11228052	-0.011447233	0.030017588
ASCL1 cg14794428	-0.002110542	0.579469321

ASCL1 cg16712637	-0.000174087	0.974617716
ASCL1 cg16921310	-0.001664361	0.911411176
ASCL1 cg17015844	-0.002897518	0.634575379
ASCL1 cg20053158	-0.005436232	0.036172557
ASCL1 cg22154024	-0.000599841	0.90469773
ASCL1 cg22356339	-0.001177586	0.867203362
ASCL1 cg25734490	-0.003623736	0.32099803
ASCL1 cg26866482	-0.004199431	0.402155742
ASCL1 cg27420520	-0.000461612	0.895680955
ASCL1 cg27569040	-0.003413897	0.321736856
ASPM cg03750742	0.007430442	0.477751994
ASPM cg07346077	0.000823674	0.670799333
ASPM cg10212558	-0.00275131	0.411849926
ASPM cg10519406	0.001695201	0.580698053
ASPM cg11215015	0.011254807	0.705932117
ASPM cg11336294	-0.001427556	0.791214734
ASPM cg12528753	0.001202956	0.829247505
ASPM cg13073409	-0.00903459	0.691091681
ASPM cg13650137	-0.001881355	0.737990322
ASPM cg14212631	-0.000375555	0.96622384
ASPM cg16552589	-0.001588784	0.810574327
ASPM cg19350340	-0.002003377	0.577600434
ASPM cg24401645	0.002374414	0.702257808
ATF3 cg01064286	-0.034039613	0.327778743
ATF3 cg01668452	-0.00309132	0.909525172
ATF3 cg02636234	-0.006912706	0.305009067
ATF3 cg03717803	-0.004035877	0.182901128
ATF3 cg04203508	-0.02406342	0.002281661
ATF3 cg05036120	-0.04244999	0.007578291
ATF3 cg07139026	-0.012332452	0.494887946
ATF3 cg07199812	-0.001046542	0.830358469
ATF3 cg10103631	0.000893211	0.820144908
ATF3 cg10985914	0.193598595	1.50204E-12
ATF3 cg13230348	-0.002901235	0.549373491
ATF3 cg13243329	-0.000542458	0.862106421
ATF3 cg15831613	0.00460767	0.887750663
ATF3 cg17080534	-0.003543549	0.6328169
ATF3 cg18174834	-0.001909547	0.80236872
ATF3 cg18317154	-0.034891993	0.34393291
ATF3 cg18350796	-0.007104866	0.170527033
ATF3 cg19519612	-0.005727051	0.321138193
ATF3 cg20169506	0.004532186	0.166942409
ATF3 cg21160472	-0.001798761	0.949987815
ATF3 cg21281234	-0.001440779	0.893958421
ATF3 cg21395152	-0.011043032	0.720957877
ATF3 cg23020752	-0.01673109	0.18158993
ATF3 cg23534142	-0.089976141	0.002446341
ATF3 cg24141233	-0.003718048	0.745481824
ATF3 cg24365042	-0.0198477	0.314796813

ATF3 cg25386151	-0.022701073	0.060959597
ATP1B3 cg03278965	0.002600497	0.78457982
ATP1B3 cg09459719	-0.000240604	0.976950092
ATP1B3 cg16419706	-0.005655744	0.166636508
ATP1B3 cg17322125	-0.001809844	0.776355298
ATP1B3 cg19936194	-0.001357228	0.756105907
ATP1B3 cg24382918	0.020868795	0.673871027
ATP1B3 cg25798112	-0.001372827	0.634312168
ATP1B3 cg26675219	-0.000460252	0.942122289
AURKA cg01596892	2.09981E-05	0.995997868
AURKA cg03139435	-0.01333975	0.726539535
AURKA cg03776827	-0.003688735	0.273084213
AURKA cg04444873	-0.000272118	0.943678171
AURKA cg09712306	0.092536011	5.07025E-06
AURKA cg14275127	-0.00016175	0.979830123
AURKA cg16417909	-5.46997E-05	0.999270223
AURKA cg19506755	-0.002786216	0.544719828
AURKA cg21763021	0.000217416	0.945676786
AURKA cg21772838	-0.002366921	0.562842209
AURKA cg22732672	0.001720785	0.461672761
AURKA cg22989919	-0.006937198	0.128144473
AURKA cg24941405	-0.00010969	0.978004582
AURKA cg27590859	0.002988862	0.688416333
AURKAIP1 cg01508988	-0.016561665	0.212157641
AURKAIP1 cg02144516	0.037710508	0.032896944
AURKAIP1 cg07395004	-0.008913496	0.051778403
AURKAIP1 cg09864992	-0.020762107	0.086377297
AURKAIP1 cg14094639	-0.024157278	0.335534211
AURKAIP1 cg15604233	0.001120446	0.809944267
AURKAIP1 cg15617543	-0.001494493	0.808393661
AURKAIP1 cg19823490	-0.004011708	0.514617333
AURKAIP1 cg19871494	-0.002392257	0.824616085
AURKAIP1 cg26865478	-0.006385359	0.868858348
AURKB cg02245743	1.12354E-05	0.998561009
AURKB cg04612488	-0.000385516	0.963171378
AURKB cg05617798	-0.001466291	0.803373149
AURKB cg05811247	0.006516426	0.24973696
AURKB cg09132923	-0.033560239	0.16860039
AURKB cg11009596	-0.004538066	0.317008878
AURKB cg12844497	0.0023165	0.623899525
AURKB cg13678641	-0.002046883	0.744939924
AURKB cg17365525	-0.007139718	0.88347342
AURKB cg18576335	0.00022577	0.988870795
AURKB cg21724796	0.003123093	0.877003186
AURKB cg26183021	-0.007993486	0.893477997
B2M cg00079638	0.001932268	0.623195515
B2M cg00837838	0.001854189	0.362662841
B2M cg03355298	0.001668684	0.608539775
B2M cg07192821	-0.001381446	0.792784039

B2M cg08188779	0.009093115	0.559468713
B2M cg08350173	-0.000789138	0.783886427
B2M cg08459087	0.003430624	0.801950805
B2M cg10081723	-0.000137453	0.988162217
B2M cg13964393	0.000583674	0.909506598
B2M cg16039157	-0.002802464	0.936083493
B2M cg18555073	0.042105442	0.072126882
B2M cg18696027	0.101002779	8.1121E-07
B2M cg19404757	0.001014645	0.80418725
B2M cg24134304	0.001233233	0.807828891
BCAN cg00502190	-0.036801637	0.384213156
BCAN cg00765922	0.005678397	0.824293967
BCAN cg02599463	-0.007105952	0.155234255
BCAN cg03265671	-0.055764789	0.151522544
BCAN cg04843111	-0.028091214	0.217431451
BCAN cg05654780	-0.04636532	0.000265925
BCAN cg05888917	-0.003186367	0.391925727
BCAN cg06929846	0.008301026	0.572282638
BCAN cg08465120	0.037771765	0.044068883
BCAN cg09182138	-0.038213421	0.000376308
BCAN cg10389771	0.054239684	0.095978295
BCAN cg12664038	-0.027479049	0.000236365
BCAN cg13069892	-0.147114626	6.62731E-07
BCAN cg13950948	0.002384669	0.579920265
BCAN cg14423692	-0.017011542	0.000355178
BCAN cg16269733	0.047392934	0.215310724
BCAN cg19321979	-0.065523117	6.34985E-06
BCAN cg20270188	-0.021938698	0.000958183
BCAN cg21475402	-0.018883071	0.003575384
BCAN cg22168255	0.000806628	0.879151483
BCAN cg23640929	-0.161901586	1.64453E-08
BCAN cg25773262	0.014217953	0.693344057
BCL7C cg00283876	-0.059453959	0.00086611
BCL7C cg05386903	-0.00341131	0.555859393
BCL7C cg06815754	-0.000275533	0.930841256
BCL7C cg07013258	-0.038799846	2.8512E-06
BCL7C cg07378245	-0.004275985	0.280367168
BCL7C cg08297053	-0.00367449	0.551663485
BCL7C cg10337500	0.005257203	0.849316059
BCL7C cg14290127	-0.011167697	0.015412898
BCL7C cg16657816	-0.001133196	0.724606036
BCL7C cg22377963	-0.045993999	1.62563E-09
BCL7C cg27420156	-0.000963022	0.86965839
BCL7C cg27609508	-0.002678976	0.831249945
BIRC5 cg00017271	-0.187032038	5.00085E-12
BIRC5 cg01886741	-0.001229929	0.702959525
BIRC5 cg04271999	-0.008495785	0.475459899
BIRC5 cg04972436	-0.014032263	0.708069012
BIRC5 cg07366188	-0.189180367	1.03748E-09

BIRC5 cg10070788	-0.176462371	4.72707E-08
BIRC5 cg10140240	-0.233278607	2.24539E-11
BIRC5 cg11912239	-0.143337454	1.15321E-09
BIRC5 cg15270350	-0.09189201	1.66352E-06
BIRC5 cg17515702	0.002009715	0.812537951
BIRC5 cg21070864	-0.000206632	0.952561575
BIRC5 cg23302638	0.00065527	0.938036224
BIRC5 cg25986496	-0.003947094	0.409387286
BTG1 cg04100724	-0.004581136	0.472570783
BTG1 cg04211745	-0.005322472	0.039345392
BTG1 cg05819371	-0.002454079	0.648585032
BTG1 cg06551025	-0.000787509	0.748407652
BTG1 cg08832851	-0.025503385	0.001205155
BTG1 cg09918929	-9.0996E-05	0.991321909
BTG1 cg13132650	-0.001311811	0.84695018
BTG1 cg20078640	-0.001635735	0.803172896
BTG1 cg21381360	-0.000869584	0.768582224
BTG1 cg25218905	-0.000539372	0.895492991
BUB1 cg01114573	-0.000159746	0.975970155
BUB1 cg02066650	0.002815674	0.725534741
BUB1 cg03208501	-0.000834086	0.917817229
BUB1 cg03292206	-0.163991142	1.20094E-07
BUB1 cg10416105	-0.005121717	0.386628234
BUB1 cg10954392	0.001158589	0.841761956
BUB1 cg15054972	-0.010631186	0.105874785
BUB1 cg18518914	-0.004724986	0.073729523
BUB1 cg19177701	-0.002493856	0.862971463
BUB1 cg23198623	0.075723282	0.000328458
BUB1 cg26628795	0.003595896	0.691383755
BUB1 cg27210766	0.002609472	0.350898158
BUD31 cg01115668	-0.00430286	0.180949873
BUD31 cg01916058	0.008629311	0.793079017
BUD31 cg02798801	-0.009012638	0.824158702
BUD31 cg03695448	-0.006770367	0.038386041
BUD31 cg06688409	-0.000565336	0.92265759
BUD31 cg07477109	-0.005433358	0.717917904
BUD31 cg10213821	0.026100272	0.589027204
BUD31 cg10883583	-0.001950258	0.729803144
BUD31 cg11724156	-0.01413671	0.371619142
BUD31 cg16327497	0.001600512	0.48997761
BUD31 cg16415856	-0.005493672	0.166912971
BUD31 cg17841065	-0.001247119	0.901358542
BUD31 cg18634506	0.021735791	0.676573656
BUD31 cg21786957	-0.000806531	0.893616942
BUD31 cg24990989	0.025619106	0.443277074
BUD31 cg25782445	0.004515991	0.597348804
BUD31 cg26259610	-0.000252011	0.951384331
BUD31 cg27334224	-0.00482764	0.620798182
CALM1 cg01267274	-0.000695873	0.935601686

CALM1 cg01311654	-0.001315656	0.714066416
CALM1 cg01579928	-0.000221768	0.956306075
CALM1 cg02272150	4.37004E-05	0.992395135
CALM1 cg03062665	-0.001388568	0.758957804
CALM1 cg04712435	-0.003555592	0.55847342
CALM1 cg05077358	0.011720093	0.681312739
CALM1 cg07150772	-0.001804042	0.784374439
CALM1 cg13481335	-0.00178996	0.845175869
CALM1 cg15930145	0.000312375	0.972664814
CALM1 cg18079499	-0.022943043	0.226180299
CALM1 cg18087520	-0.023640879	0.542893792
CALM1 cg18858717	-0.000526416	0.858960568
CALM1 cg20250935	-0.029632997	0.310736742
CALM1 cg20328328	-0.001224332	0.704619971
CALM1 cg21086878	-0.00251461	0.642898922
CALM1 cg22979624	-0.002486538	0.514194757
CALM1 cg23047920	0.001909871	0.812541948
CALM1 cg26007849	-0.008107753	0.726238939
CALM2 cg01600782	0.000219461	0.94670453
CALM2 cg03034696	-0.052316211	0.001790935
CALM2 cg03545677	-0.000860665	0.800281722
CALM2 cg09707038	-0.005622422	0.636059722
CALM2 cg09920725	-0.00312972	0.382889433
CALM2 cg09942741	-0.00134336	0.85388394
CALM2 cg10805483	-0.001096913	0.943861676
CALM2 cg12478290	-0.005393099	0.390939118
CALM2 cg16653203	0.001901345	0.529628867
CALM2 cg19536127	-0.005928252	0.276044685
CALM2 cg20203588	0.00116555	0.694591052
CALM2 cg21029591	-0.002437496	0.59298336
CALM2 cg21361646	-0.04664686	0.042179615
CALM2 cg24431318	-0.002143733	0.849193615
CALM2 cg27073431	0.083297289	4.66123E-07
CASC5 cg00091781	0.000187723	0.966864471
CASC5 cg00235645	0.004517041	0.305692886
CASC5 cg00437399	-0.00955118	0.518432261
CASC5 cg03355190	-0.000643753	0.882143281
CASC5 cg03605064	0.002844202	0.665801888
CASC5 cg07106138	-0.006109381	0.385411479
CASC5 cg09861958	0.001660087	0.443112781
CASC5 cg16228087	0.003682282	0.55106199
CASC5 cg17355835	0.001788358	0.657317605
CASC5 cg18582520	-0.01590234	0.463652341
CASC5 cg20314325	-0.001395136	0.643932297
CASC5 cg26431595	0.003127339	0.238168993
CASC5 cg27279253	0.002587316	0.569897087
CASC5 cg27659014	-0.001588786	0.830163607
CCDC109B cg00131899	0.001758478	0.867168869
CCDC109B cg01442334	-0.001596369	0.845005295

CCDC109B cg02105485	0.002132852	0.520069103
CCDC109B cg03005261	-0.005760034	0.911116751
CCDC109B cg03591709	0.002946165	0.436370613
CCDC109B cg05248542	-0.03546057	0.294736095
CCDC109B cg05702851	0.000792846	0.973923576
CCDC109B cg11498516	-0.028361399	0.054372687
CCDC109B cg12309813	-0.099518824	9.17465E-06
CCDC109B cg15209369	-0.038190669	0.004101989
CCDC109B cg17756860	-0.003860647	0.875075139
CCDC109B cg19563331	0.001061186	0.847487875
CCDC109B cg19905837	-0.00176011	0.639965425
CCDC109B cg20886165	0.002306499	0.313083472
CCDC109B cg21837443	-0.002727376	0.494374549
CCDC109B cg23196549	-0.006130182	0.924511812
CCNA2 cg01854459	-0.001940648	0.562080374
CCNA2 cg04314790	-0.000130614	0.964813226
CCNA2 cg04512424	-0.000432452	0.840577477
CCNA2 cg06762614	0.000439212	0.947654408
CCNA2 cg06817840	0.004287098	0.536914474
CCNA2 cg06952910	-6.36731E-05	0.998133953
CCNA2 cg07263562	-0.069436543	0.015802112
CCNA2 cg07806866	-0.004343079	0.422423614
CCNA2 cg08201690	0.008110224	0.023163366
CCNA2 cg10002561	-0.000126944	0.977111926
CCNA2 cg18913527	0.000533602	0.911961804
CCNA2 cg20391652	-0.002640221	0.302271054
CCNA2 cg20573242	-0.001382348	0.663173641
CCNA2 cg20844771	0.007154474	0.004489953
CCNA2 cg22219489	0.00121264	0.683917157
CDCA3 cg07446572	-0.029263248	0.541291025
CDCA3 cg15942562	-0.035514438	0.189858636
CDCA3 cg25700897	-0.0351497	0.033348792
CDK1 cg02401235	-0.002209296	0.621599974
CDK1 cg04271103	-0.000840246	0.821889406
CDK1 cg06793798	0.007552926	0.298068606
CDK1 cg13227473	-0.002948953	0.344706131
CDK1 cg13554667	0.003142808	0.937699877
CDK1 cg13954297	-0.001009655	0.832630293
CDK1 cg14922279	-0.002845693	0.413190639
CDK1 cg15172601	-0.001529221	0.777991034
CDK1 cg17778722	-0.012445571	0.461489417
CDK1 cg19147990	-0.006088753	0.349908696
CDK1 cg23973115	-0.08564302	0.005218867
CDK1 cg25228510	0.004965375	0.666341999
CDK1 cg25793692	0.001311933	0.750384659
CDK1 cg27457323	0.00109583	0.719035684
CDKN3 cg00649500	0.004510196	0.758840429
CDKN3 cg01209943	-0.006483145	0.313708164
CDKN3 cg02252340	-0.002486405	0.811795739

CDKN3 cg02626977	-0.001164194	0.853507652
CDKN3 cg03724882	-0.007570922	0.686359486
CDKN3 cg04276794	-0.003260051	0.266176693
CDKN3 cg04290510	0.00298384	0.92154878
CDKN3 cg09988663	-0.012267925	0.406118779
CDKN3 cg14144732	-0.000403876	0.9907812
CDKN3 cg18471460	-0.007721527	0.422582785
CDKN3 cg20454620	0.000266227	0.958793092
CDKN3 cg20908785	6.72765E-06	0.999707344
CDKN3 cg24032252	-0.086493838	0.003333275
CENPA cg03666973	-0.000575384	0.913877161
CENPA cg03798852	0.00051619	0.85657277
CENPA cg04749129	-0.008006116	0.346611987
CENPA cg10315993	-0.018097205	0.361417163
CENPA cg10480329	0.075644983	2.41185E-07
CENPA cg13874838	-0.001469933	0.679173855
CENPA cg14934766	-0.000434288	0.965743508
CENPA cg17206978	-0.002998205	0.491171596
CENPA cg21023052	-0.002356454	0.321495518
CENPA cg21360828	-0.016545181	0.283063412
CENPA cg21450708	-0.003755671	0.873827344
CENPA cg22284448	-0.089226851	1.29101E-05
CENPA cg24127278	-0.003787636	0.924959563
CENPA cg25942688	-0.004709093	0.834915209
CENPA cg27025088	0.006075325	0.509785815
CENPF cg00620223	-0.004172996	0.630536326
CENPF cg00987890	-0.008875708	0.219634084
CENPF cg03327327	-0.004160962	0.829071983
CENPF cg04552378	0.004174947	0.651297823
CENPF cg05794695	-0.006014144	0.628149069
CENPF cg07140595	-0.001549201	0.762408726
CENPF cg09065776	-0.000979926	0.837716415
CENPF cg09885951	-0.000676597	0.847451783
CENPF cg09933323	-0.041115668	0.339019839
CENPF cg10379951	0.001336187	0.879306213
CENPF cg10716356	-0.056562588	0.025558782
CENPF cg13085338	-0.000102508	0.976195779
CENPF cg15009294	0.003318137	0.713097821
CENPF cg15639974	-0.004187568	0.154437946
CENPF cg15722631	-0.003923954	0.928123876
CENPF cg16327647	-0.008848892	0.079081132
CENPF cg17750334	-0.00340171	0.263599326
CENPF cg21164723	0.001197216	0.805388452
CENPF cg21693965	-0.07269509	0.001116707
CENPF cg22041712	-0.022961457	0.523844611
CENPF cg22426789	0.003303881	0.78864363
CENPF cg25284075	-0.00339202	0.62198421
CENPK cg00984787	-0.026364623	0.183253162
CENPK cg01403114	-0.005707332	0.349882379

CENPK cg11044823	0.002492271	0.781099262
CENPK cg11190754	-0.005123065	0.516862967
CENPK cg13034654	-0.001669994	0.76335961
CENPK cg18192574	0.013779211	0.379623094
CENPK cg18503711	-0.003185444	0.665295543
CENPK cg22719655	-0.03826576	0.022795648
CENPK cg23818046	-0.000165106	0.977905396
CFL1 cg02715140	0.001575586	0.52569798
CFL1 cg06443583	-0.003482482	0.70115056
CFL1 cg08425745	0.000361314	0.958774086
CFL1 cg10983586	-0.001527633	0.788447433
CFL1 cg11042866	-0.002367286	0.620890149
CFL1 cg12815271	-0.002014708	0.688916374
CFL1 cg12849795	0.001053426	0.954571253
CFL1 cg14395634	0.00260213	0.556430449
CFL1 cg16985113	0.000564378	0.893477997
CFL1 cg20909159	-0.013858314	0.268079388
CFL1 cg22068741	-0.001061424	0.897858347
CFL1 cg22548788	-0.002803802	0.514088784
CHCHD3 cg14187409	-0.004542561	0.464870011
CHCHD3 cg14577835	-0.002411049	0.85569339
CHCHD3 cg14936778	-0.008185482	0.258918583
CHCHD3 cg17755880	-0.041168747	0.368661106
CHCHD3 cg19717492	-0.002458532	0.631110849
CHCHD3 cg21096502	-0.001663137	0.708008142
CHCHD3 cg21424664	0.008671581	0.822364756
CHCHD3 cg22383121	-0.061988192	0.061914369
CHCHD3 cg25408620	-0.00184937	0.918480977
CHCHD3 cg25529965	0.000326419	0.908771777
CHCHD3 cg26399411	0.000432359	0.98189765
CHCHD3 cg00032852	-0.004106989	0.490471057
CHCHD3 cg00446758	-0.019393126	0.016164099
CHCHD3 cg02204578	-0.009412725	0.165181429
CHCHD3 cg07486100	-0.002201529	0.739457996
CHCHD3 cg07590705	0.003848267	0.676508416
CHCHD3 cg08734988	-0.005628442	0.17850301
CHCHD3 cg08757624	-0.087067957	0.000164477
CHCHD3 cg10488050	-0.185736864	7.55381E-06
CHCHD3 cg10590628	0.000279641	0.960820363
CHCHD3 cg11369172		
CHCHD3 cg13476800		
CHCHD3 cg14583365		
CHCHD3 cg15373257		
CHCHD3 cg16770546		
CHCHD3 cg18047684		
CHCHD3 cg18987274		
CHCHD3 cg19632026		
CHCHD3 cg19918623		
CHCHD3 cg21025171		

CKAP2 cg04566791	-0.003242882	0.411727209
CKAP2 cg07533350	5.53274E-05	0.993827156
CKAP2 cg07898084	-0.000403755	0.903490441
CKAP2 cg08997021	-0.006817182	0.647351173
CKAP2 cg10218426	-0.00206039	0.601141956
CKAP2 cg10728060	-0.001110465	0.713423644
CKAP2 cg12988843	0.000572708	0.907815483
CKAP2 cg14221264	-0.005856031	0.298744377
CKAP2 cg14425257	0.001391134	0.720872751
CKAP2 cg23721425	0.005126426	0.760651861
CKAP2 cg24469095	0.00373579	0.457658877
CKAP2 cg26660792	0.004859863	0.815564239
CKS1B cg00993057	0.000462471	0.888469097
CKS1B cg08733086	0.001777867	0.855239529
CKS1B cg22234827	6.68566E-05	0.989350221
CKS1B cg25017976	0.004437111	0.80420494
CLIC1 cg00140361	-0.003830735	0.808637481
CLIC1 cg00579105	0.030750133	0.000586669
CLIC1 cg00657529	0.003445269	0.727163376
CLIC1 cg01578324	0.07358951	4.45878E-05
CLIC1 cg03619586	0.041096954	0.205145743
CLIC1 cg04658027	0.049056913	0.000848063
CLIC1 cg07404514	-0.002949316	0.906071712
CLIC1 cg08254733	0.003624144	0.913671321
CLIC1 cg09887589	0.073192288	0.000145946
CLIC1 cg10041232	0.062479183	0.026043297
CLIC1 cg11093373	0.02346178	0.010133036
CLIC1 cg12506597	-0.055452969	0.145956048
CLIC1 cg12921375	0.024134064	0.007038077
CLIC1 cg13026137	0.04385248	0.09600182
CLIC1 cg13622821	0.003648985	0.800906885
CLIC1 cg14138132	0.035301748	0.000394286
CLIC1 cg16587838	0.095557592	4.3594E-09
CLIC1 cg17069739	0.004769811	0.209953846
CLIC1 cg18402034	-0.049831245	0.001236629
CLIC1 cg18909389	-0.030083729	0.029371872
CLIC1 cg20165831	0.001451052	0.955039151
CLIC1 cg21769117	0.096053734	8.22189E-08
CLIC1 cg23065732	0.013683752	0.684327758
CLIC1 cg23425079	0.104093933	4.17176E-06
CLIC1 cg24208375	0.022955578	0.012839109
CLIC1 cg25588725	0.005119702	0.516174019
CLU cg00359590	0.033250079	0.235559942
CLU cg00928580	0.020242317	0.277210517
CLU cg01572979	0.00093443	0.935649591
CLU cg01916918	-0.149953515	3.5944E-05
CLU cg02393097	0.011431811	0.370593291
CLU cg03735511	0.007452927	0.501003464
CLU cg04929736	-0.002439873	0.397780605

CLU cg05499834	0.002530216	0.778261083
CLU cg08594681	-0.012565466	0.189537298
CLU cg09969545	0.000272484	0.984668411
CLU cg11467637	-0.004387885	0.417051635
CLU cg11694223	0.007437013	0.250636086
CLU cg11697169	-0.008013828	0.716015435
CLU cg11775417	0.000479684	0.928653555
CLU cg11783834	0.033200697	0.041383857
CLU cg12729838	-0.02413152	0.001927641
CLU cg13159325	-0.006454016	0.049832853
CLU cg13488078	0.001792646	0.932796603
CLU cg13620439	0.016141698	0.501418099
CLU cg14917244	-0.01160151	0.029960287
CLU cg16292768	-0.005361694	0.925314577
CLU cg19442470	-0.059537587	0.001474272
CLU cg19549068	-0.0022148	0.46855968
CLU cg20504077	-0.002531829	0.544983309
CLU cg21739400	-0.004107392	0.318251173
CLU cg22313574	-0.011490708	0.227251219
CLU cg22738440	-0.00196452	0.850256912
CLU cg23460950	-0.012631208	0.010125412
CLU cg23548686	-0.000249802	0.961963188
CLU cg24030037	0.001350604	0.879956043
CLU cg24257038	-3.93907E-05	0.99480484
CLU cg25302704	0.083002398	0.031288845
CNN3 cg01045913	-0.004207233	0.871399802
CNN3 cg02718725	-0.002692806	0.534283321
CNN3 cg02835561	-0.01248736	0.228960724
CNN3 cg07823585	-0.002272331	0.758660811
CNN3 cg08940075	0.078059725	0.02109977
CNN3 cg10992558	-0.001508422	0.958294357
CNN3 cg12448539	-0.001435521	0.824781793
CNN3 cg12613632	0.067913271	0.045076862
CNN3 cg14669167	-0.00180227	0.400931434
CNN3 cg15496336	0.002874746	0.63437294
CNN3 cg18158385	-0.00227013	0.462262165
CNN3 cg18344652	-0.003530476	0.458935156
CNN3 cg19164761	-0.053991084	0.02552851
CNN3 cg22045340	-0.000331496	0.980397555
CNN3 cg22333836	0.000435608	0.920870198
CNN3 cg24663131	-0.00172209	0.774163707
CNN3 cg24731597	0.003767388	0.606653592
CNN3 cg25026898	-0.002388106	0.489479467
CNN3 cg25311623	-0.009064094	0.803126331
CNN3 cg26227186	-0.003088935	0.826906982
CNN3 cg26619317	-0.009312699	0.051677238
CNN3 cg27231335	-0.009939415	0.031029232
COX5A cg00023695	0.001257023	0.770347674
COX5A cg00926162	-0.001851672	0.424649879

COX5A cg01737828	-0.000770534	0.855376061
COX5A cg02035565	-0.010634435	0.54012426
COX5A cg04249030	-0.000701807	0.888497596
COX5A cg10147044	-0.002922398	0.265522327
COX5A cg12478809	0.008287118	0.679601518
COX5A cg17397401	0.005060134	0.799335929
COX5A cg18817426	0.006935792	0.756171038
COX5A cg25250073	0.001785506	0.867154181
COX5A cg27140083	0.0029907	0.572611062
COX8A cg01818597	0.001576381	0.835987633
COX8A cg04674081	-0.001525845	0.781168543
COX8A cg04912466	-0.001722893	0.858990893
COX8A cg07104773	-0.001901963	0.798052749
COX8A cg08063013	-0.002632607	0.363298528
COX8A cg10297721	-0.001727325	0.685644633
COX8A cg11338389	-0.000934067	0.946858655
COX8A cg14265380	0.007201477	0.215104768
COX8A cg17292384	-0.041393981	0.335664708
COX8A cg19806933	-0.003200579	0.556245856
CRYAB cg00514609	-0.014939946	0.396813121
CRYAB cg07476508	-0.020822881	0.267102633
CRYAB cg10048349	-0.022051823	0.119013795
CRYAB cg11694519	0.010626994	0.890864964
CRYAB cg13084335	-0.051080941	5.68069E-11
CRYAB cg15204861	-0.039439725	0.036386626
CRYAB cg15545878	-0.068419573	1.26903E-10
CTNNAL1 cg02446003	0.002056743	0.759122518
CTNNAL1 cg05485060	-0.010454263	0.745477813
CTNNAL1 cg13546538	0.030721427	0.055766366
CTNNAL1 cg13813623	0.000485933	0.921239617
CTNNAL1 cg13832679	-0.003754211	0.788629745
CTNNAL1 cg14442329	0.002522639	0.816862357
CTNNAL1 cg14452121	-0.003654388	0.772760406
CTNNAL1 cg14487878	0.001763616	0.826721398
CTNNAL1 cg15459742	-0.001637881	0.609549172
CTNNAL1 cg16969852	0.000376358	0.955039151
CYR61 cg03519711	-0.000240188	0.948862332
CYR61 cg04453065	-0.001039972	0.73304293
CYR61 cg05395648	-0.005575831	0.146540675
CYR61 cg06805280	-0.00260201	0.449365155
CYR61 cg07165167	0.039446616	0.240253594
CYR61 cg07350718	0.006814025	0.148992064
CYR61 cg07561554	-0.003946883	0.379310209
CYR61 cg07602008	0.002999416	0.522183111
CYR61 cg11750851	-0.044497145	5.11121E-06
CYR61 cg12278705	0.056973006	0.068737979
CYR61 cg13206932	0.038025676	0.264015784
CYR61 cg13871826	0.033082771	0.118370283
CYR61 cg13984515	-0.007323461	0.108096961

CYR61 cg15648041	-0.022038744	0.402776109
CYR61 cg17319576	0.000723376	0.916112446
CYR61 cg17499345	0.002735007	0.856043541
CYR61 cg18027683	-0.010345151	0.716750366
CYR61 cg20293609	0.005906931	0.098443456
CYR61 cg22123915	0.067221598	0.038529436
CYR61 cg23785940	-0.011306138	0.00374251
CYR61 cg24692177	-0.014361496	0.000449873
CYR61 cg25720697	-0.007683498	0.043498578
DCX cg02103689	-0.001321987	0.727131267
DCX cg02404933	0.000157614	0.989861035
DCX cg04563643	0.001636739	0.821806811
DCX cg06247470	-0.003337333	0.322392363
DCX cg07073120	-0.008461055	0.067368741
DCX cg07847925	-0.00847862	0.227078372
DCX cg08897132	-0.002789872	0.634203996
DCX cg10636821	0.000391797	0.980212152
DCX cg10723012	-0.001672196	0.697567018
DCX cg18605491	-0.006456236	0.147584596
DCX cg20757526	-0.004626411	0.477037058
DCX cg27226927	0.009034649	0.627919867
DLGAP5 cg05225373	0.001974951	0.528267924
DLGAP5 cg13517138	-0.007389513	0.449050006
DLGAP5 cg18941521	0.003519395	0.848511705
DLGAP5 cg23678254	0.106206615	1.76646E-06
DLGAP5 cg24733298	4.17522E-07	0.999954443
DLGAP5 cg25465634	-0.008467208	0.760656778
DLL1 cg00084338	-0.055315739	0.000306747
DLL1 cg00356645	-0.083590435	0.000593904
DLL1 cg00474209	-0.007857178	0.042193709
DLL1 cg00494883	-0.00208333	0.585133332
DLL1 cg00839434	-0.001672186	0.594182097
DLL1 cg01475503	-0.029226764	0.042955073
DLL1 cg03228386	-0.012431819	0.109533665
DLL1 cg03394401	-0.014875096	0.191318998
DLL1 cg03815571	-0.000724061	0.911370493
DLL1 cg05228964	-0.116324988	2.04475E-05
DLL1 cg06432036	-0.093610772	1.3931E-05
DLL1 cg07598357	-0.142436695	7.88266E-08
DLL1 cg11519760	-0.150196506	2.24526E-05
DLL1 cg12024311	0.001053224	0.78733835
DLL1 cg12581730	-0.001836559	0.709865818
DLL1 cg13250324	-0.024861675	0.107643824
DLL1 cg13356429	-0.00076345	0.441556626
DLL1 cg13494348	-0.153697794	1.48092E-08
DLL1 cg14347219	-0.083670742	0.012228654
DLL1 cg14497851	-0.002878121	0.510805878
DLL1 cg15319528	-0.020425059	0.005452615
DLL1 cg15931921	-0.14946891	3.19889E-05

DLL1 cg17586256	-0.021900582	0.375983458
DLL1 cg18239372	-0.000273936	0.958462904
DLL1 cg19189355	-0.039750077	0.004405671
DLL1 cg19524023	-0.066085373	0.000176931
DLL1 cg20926049	-0.048795236	5.23715E-05
DLL1 cg21293831	-0.007903556	0.508988229
DLL1 cg23191576	-0.126110219	7.14366E-06
DLL1 cg25372103	-0.011326131	0.027445897
DLL1 cg25389470	-0.001608667	0.845222499
DLL1 cg27139933	0.000905265	0.973559078
DLL1 cg27246129	-0.046344863	0.000794148
DLL1 cg27614489	-0.017241586	0.000502965
DLL3 cg00323695	-0.010532301	0.006837553
DLL3 cg01519877	-0.008119107	0.153792765
DLL3 cg01590848	0.032627074	0.355532982
DLL3 cg02873868	-0.001187727	0.900205348
DLL3 cg03139057	-0.002262632	0.355205842
DLL3 cg03807316	0.05067645	0.093483062
DLL3 cg06664357	-0.067727006	0.172168312
DLL3 cg07640648	0.008426755	0.896613615
DLL3 cg08145231	-0.004167737	0.564031872
DLL3 cg08551532	0.052291894	0.091177291
DLL3 cg09431525	-0.090999224	0.004412646
DLL3 cg10832304	0.027376896	0.545283501
DLL3 cg11680792	-0.006488676	0.464354238
DLL3 cg14925296	-0.045989653	7.25579E-05
DLL3 cg15132673	0.039156755	0.30517816
DLL3 cg15412721	-0.028572604	0.112016609
DLL3 cg17945053	-0.075738456	0.011890315
DLL3 cg19183842	-0.088175248	0.017369597
DLL3 cg21718857	-0.001953511	0.644450827
DLL3 cg22174088	-0.001097337	0.846632062
DLL3 cg25425784	-0.013781633	0.086414319
DLL3 cg25627144	-0.000692664	0.952711947
DLL3 cg26536750	-0.02050824	0.114745995
DLL3 cg26801047	-0.00430351	0.5709431
DNJAB1 cg00395795	-0.005828637	0.262118203
DNJAB1 cg01083584	0.013268709	0.275809396
DNJAB1 cg05514743	-0.008040106	0.082007964
DNJAB1 cg07813370	0.000492107	0.991986778
DNJAB1 cg13682437	-0.003535917	0.447759659
DNJAB1 cg15712267	-0.03785041	0.063860453
DNJAB1 cg19799760	-0.002248503	0.661594642
DNJAB1 cg23889338	-0.001018328	0.821325446
DNJAB1 cg24714147	-0.001887004	0.770331176
DTYMK cg00130180	-0.068516474	0.053617592
DTYMK cg00590500	-0.005327764	0.665359838
DTYMK cg00969323	-0.067430299	0.086471283
DTYMK cg02823437	-0.05159954	0.014848218

DTYMK cg03301433	-0.014651644	0.177120983
DTYMK cg05880944	-0.061725836	0.000308354
DTYMK cg06974460	-0.012281463	0.54778289
DTYMK cg08081485	-0.122186881	0.000465466
DTYMK cg10460130	-0.011868196	0.193100522
DTYMK cg10888811	-0.052741922	0.004640697
DTYMK cg11383961	-0.001554461	0.919999023
DTYMK cg12508009	0.002573678	0.705903674
DTYMK cg12570942	-0.008613527	0.335380673
DTYMK cg13800586	-0.006688261	0.192687018
DTYMK cg15720975	-0.004140348	0.442158057
DTYMK cg15734942	-0.105864399	0.000320384
DTYMK cg16760712	-0.051382752	7.24636E-05
DTYMK cg18361031	-0.003260284	0.363419705
DTYMK cg19024381	-0.001947253	0.716335136
DTYMK cg19288752	-0.00223162	0.71648875
DTYMK cg19463885	-0.005848506	0.045082978
DTYMK cg19935506	-0.003787231	0.356917298
DTYMK cg21478921	-0.018837404	0.034719392
DTYMK cg23232749	-0.006312962	0.52759517
DTYMK cg24414737	-0.06479337	0.003364133
EEF1B2 cg04202957	-0.059486898	4.38615E-07
EEF1B2 cg10188403	0.002583104	0.47150003
EEF1B2 cg12608507	0.00216781	0.97319508
EEF1B2 cg12907060	-0.000708528	0.793349301
EEF1B2 cg13582873	0.000491075	0.947954769
EEF1B2	-0.036375439	0.198105881
EEF1B2 cg19638845	-0.001949904	0.882257685
EEF1B2 cg19656890	0.001433259	0.532404098
EEF1B2 cg21166785	-0.002210466	0.618923695
EEF1B2 cg21660531	0.005338796	0.342502798
EEF1B2 cg22929018	0.001012623	0.787116402
EEF1B2 cg24330485	-0.000375098	0.908852917
EEF1B2 cg25099438	0.003717752	0.585181349
EEF1B2 cg27248887	-0.002153583	0.971070416
ETFB cg02666184	-0.000555493	0.917055423
ETFB cg02837212	-0.001264048	0.795500792
ETFB cg04322978	-0.003795408	0.490749144
ETFB cg08854169	0.000512435	0.880815031
ETFB cg10219093	-0.000622621	0.876682878
ETFB cg19497071	-0.004182085	0.560249714
ETFB cg22469897	-0.111169833	0.003087162
ETFB cg23851027	-0.057108515	0.010986303
ETFB cg24129914	-0.000495367	0.960643182
ETV1 cg00328593	-0.000186681	0.97319508
ETV1 cg00469341	-0.000256168	0.981860643
ETV1 cg01958221	-0.002007777	0.743844567
ETV1 cg02881625	-0.000736166	0.848698853
ETV1 cg02971555	-0.008163061	0.303742437

ETV1 cg03844154	0.000769735	0.85765241
ETV1 cg03932308	0.001145038	0.89139471
ETV1 cg03985360	-0.067347711	0.004122318
ETV1 cg05131488	-0.081766792	0.001008789
ETV1 cg05605257	0.000215916	0.959168289
ETV1 cg05918135	-0.000713145	0.840325261
ETV1 cg06154860	0.000262994	0.943909624
ETV1 cg06735111	-0.001646017	0.602857783
ETV1 cg07323919	-0.001655701	0.746022268
ETV1 cg09366357	-0.000334487	0.935519067
ETV1 cg10386045	-0.000981049	0.845456894
ETV1 cg11104510	-0.001798822	0.572090543
ETV1 cg11279021	-0.003832914	0.083754315
ETV1 cg11897145	-0.000997321	0.86951059
ETV1 cg13524180	0.001296075	0.756523437
ETV1 cg15256387	-0.10261305	8.64858E-05
ETV1 cg17548881	-0.002427377	0.403932365
ETV1 cg20500248	-0.011340701	0.413688695
ETV1 cg21249126	0.001214395	0.8145458
ETV1 cg21463790	-0.059475018	4.06577E-06
ETV1 cg22479366	-0.002667036	0.527878606
ETV1 cg22748443	0.003260617	0.587911155
ETV1 cg26796083	-0.079011934	0.000795993
ETV1 cg26986937	-0.000106157	0.984259404
FAM64A cg03736603	-0.000680501	0.910411426
FAM64A cg11001581	-0.036307858	3.503E-05
FAM64A cg11090139	0.000174017	0.98809046
FAM64A cg14606680	0.001657037	0.838196868
FAM64A cg15847614	-0.001238884	0.940770327
FAM64A cg16057782	-0.000162792	0.985722116
FAM64A cg20124188	-0.026445655	2.31753E-06
FAM64A cg20479311	-0.000351278	0.966835308
FAM64A cg23465990	-0.00264339	0.764271178
FAM64A cg23545250	-0.045431135	0.001002585
FAM64A cg25288085	0.005736512	0.765088426
FAM64A cg27248073	-0.002941926	0.894262906
FAM64A cg27469199	-0.004549467	0.371199122
FAM83D cg02301527	0.01729291	0.468924419
FAM83D cg04071118	-0.005117044	0.794109021
FAM83D cg06163215	-0.000337593	0.981795361
FAM83D cg10751856	0.001049967	0.9768091
FAM83D cg13849422	0.035196219	0.368602655
FAM83D cg14647299	0.002215096	0.795100499
FAM83D cg15195292	0.025855371	0.418053467
FAM83D cg16808310	-0.005681148	0.817246701
FAM83D cg20241801	0.028874761	0.259302569
FAM83D cg21361343	0.031807624	0.257130143
FAM83D cg24314026	0.004846139	0.834201164
FAM83D cg25782652	-0.025146478	0.212285932

FAM83D cg26220336	0.005431884	0.925896518
FAM83D cg26351764	0.041528496	0.150851674
FAM83D cg27660920	0.018090274	0.420276906
FBXO5 cg00220769	0.011050545	0.064967759
FBXO5 cg01547733	-0.002496636	0.416943357
FBXO5 cg02426093	0.000596147	0.927876855
FBXO5 cg06131526	0.000100533	0.986820353
FBXO5 cg06434997	0.001109483	0.82952256
FBXO5 cg08463929	-0.015089554	0.03556839
FBXO5 cg10367364	0.000523203	0.882729939
FBXO5 cg11370748	0.083865325	0.015952516
FBXO5 cg12290002	-0.000742885	0.819608382
FBXO5 cg12551462	-0.002683186	0.445597869
FBXO5 cg14765172	-0.004589125	0.611101476
FBXO5 cg16493566	-0.001378053	0.676604408
FBXO5 cg17828223	0.000394377	0.895309217
FBXO5 cg18198306	-0.031868002	0.01628221
FBXO5 cg19384905	-0.035878236	0.030403967
FBXO5 cg20219159	-0.001125348	0.673508846
FBXO5 cg22417733	-0.022750961	0.004210284
FBXO5 cg23777444	-0.00177085	0.69427938
FBXO5 cg25401754	-0.000698711	0.877157529
FBXO5 cg25833238	0.003215993	0.424786787
FBXO5 cg25960313	-0.002576296	0.304858762
FBXO5 cg26425730	0.000259539	0.957560719
FDPS cg00034416	-0.000264501	0.968710305
FDPS cg03933784	0.000268035	0.946071405
FDPS cg05954884	-0.000306148	0.949074385
FDPS cg06979924	-0.153910813	1.26415E-05
FDPS cg08847296	0.00138828	0.631848676
FDPS cg10836036	0.00158978	0.573292901
FDPS cg12489819	-0.002652564	0.680088492
FDPS cg14026927	0.000539293	0.897432113
FDPS cg14788660	-0.001520835	0.790481847
FDPS cg15470750	-0.002459542	0.325197429
FDPS cg17142371	-9.59392E-05	0.976431014
FDPS cg21787190	7.52389E-05	0.993837913
FDPS cg23022783	-0.002217135	0.870008743
FDPS cg26316072	-0.005635863	0.814481486
FDPS cg26420739	-0.000337539	0.956587473
FIBP cg07796475	-0.005502141	0.132320729
FIBP cg14387684	-0.003522021	0.299817728
FIBP cg20853702	-0.014038105	0.020148403
FIBP cg25601275	0.000961952	0.899110776
FIBP cg26453990	-0.003514413	0.41255775
GAPDH cg00241355	-0.005642716	0.222522096
GAPDH cg00252813	-0.001797085	0.689098679
GAPDH cg02519286	-0.005340433	0.0915911
GAPDH cg03400107	-0.002381305	0.550607948

GAPDH cg06362313	-0.032898551	8.49138E-06
GAPDH cg09193981	-0.002654731	0.66294362
GAPDH cg09644986	-0.002855649	0.790483024
GAPDH cg15350627	-0.002661036	0.403087862
GAPDH cg15869694	-8.24408E-05	0.989048075
GAPDH cg18689930	-0.001610085	0.614172441
GAPDH cg20917484	0.002457748	0.515925462
GAPDH cg21225563	-0.055975866	0.006639772
GFAP cg02407342	0.043916242	0.171211376
GFAP cg03383006	-0.118937607	2.79891E-10
GFAP cg07918341	-0.104760699	1.71307E-10
GFAP cg08156531	-0.065185141	0.003382258
GFAP cg08358624	-0.101332325	7.18451E-09
GFAP cg09038914	-0.092405815	5.79661E-08
GFAP cg09639715	-0.099070165	1.71968E-08
GFAP cg16343401	0.048175227	0.187900858
GFAP cg17265120	0.01318091	0.748387717
GFAP cg18208742	-0.068687857	9.0615E-06
GFAP cg20668546	-0.00293973	0.934104483
GFAP cg20911989	0.033899145	0.246909684
GFAP cg21601845	-0.111519787	2.74492E-08
GFAP cg21944455	-0.107208073	5.2574E-11
GFAP cg22100937	-0.047360613	0.040550469
GFAP cg23016243	-0.008605407	0.791895602
GFAP cg23242944	-0.104212486	2.16261E-10
GFAP cg24797574	0.020160713	0.638181327
GNB2 cg01399255	-0.002955808	0.556149733
GNB2 cg02557189	-0.008368182	0.100160978
GNB2 cg02620388	-0.000317198	0.947438699
GNB2 cg03550794	-0.00234537	0.475567576
GNB2 cg04803128	-0.00105959	0.810760361
GNB2 cg07056567	-0.009748859	0.050552131
GNB2 cg07982057	-0.003240737	0.50678621
GNB2 cg08310216	-0.00189012	0.482852083
GNB2 cg11223864	-0.000680752	0.864180192
GNB2 cg14298577	-0.001311952	0.838764235
GNB2 cg16177440	-0.009290287	0.836999501
GNB2 cg16359985	-0.027437869	0.199558125
GNB2 cg16560679	0.002729741	0.944973881
GNB2 cg16684939	-0.002256324	0.484960271
GNB2 cg17702518	-0.00163832	0.591066555
GNB2 cg17854981	-0.024384419	0.136812834
GNB2 cg22377237	-0.001967273	0.823205027
GNB2 cg23325230	-0.0043992	0.196827133
GNB2 cg25392362	-0.000230325	0.966746787
GNB2 cg25963822	0.00770088	0.752044477
GNB2 cg26621408	-0.036296131	0.044861244
GNB2 cg27369641	-0.056603005	0.036871722
GPX4 cg00502555	-0.001011757	0.849271485

GPX4 cg01588546	-0.001363358	0.694132868
GPX4 cg01613691	-0.06520202	0.000180995
GPX4 cg04903600	0.009291455	0.799166245
GPX4 cg04928147	-0.001008036	0.900758415
GPX4 cg10732871	0.095426158	1.08395E-05
GPX4 cg13612480	0.000575276	0.872848515
GPX4 cg14682345	0.001525135	0.749875502
GPX4 cg14894245	0.022841725	0.134181639
GPX4 cg16715692	-0.00204026	0.754334858
GPX4 cg17812013	-0.004904168	0.214763165
GPX4 cg18061485	0.003214102	0.58579494
GPX4 cg18506744	0.064995032	1.68961E-08
GPX4 cg19512730	-0.001097328	0.632208242
GPX4 cg24783234	0.000955965	0.88661605
GPX4 cg25540845	0.000215889	0.977198804
GPX4 cg27106726	0.002292129	0.65987091
GSTP1 cg02659086	0.000329346	0.961296857
GSTP1 cg04920951	0.005491099	0.541380924
GSTP1 cg05244766	-0.02604273	0.469867716
GSTP1 cg06841499	0.015306323	0.511242337
GSTP1 cg06928838	0.005600738	0.363734145
GSTP1 cg09038676	0.004437578	0.552203419
GSTP1 cg11566244	0.020706023	0.433032039
GSTP1 cg19114214	0.01093043	0.751347098
GSTP1 cg22224704	-0.016791559	0.639376854
GSTP1 cg23725454	-0.012617663	0.838367944
GSTP1 cg25135322	-0.029497524	0.183599617
GSTP1 cg25866895	-0.06219665	0.105848897
GSTP1 cg26250609	0.009410719	0.332996846
GTSE1 cg01773692	-0.00021932	0.951483282
GTSE1 cg08629647	-0.00414294	0.223679693
GTSE1 cg18884805	-9.3208E-05	0.989967753
GTSE1 cg21542574	-0.013452809	0.591963406
GTSE1 cg22396353	-0.000212029	0.954149115
GTSE1 cg24015316	-0.002084922	0.443664284
GTSE1 cg24199050	-0.000167453	0.975195982
H2AFZ cg17709884	-0.000453573	0.941117479
H2AFZ cg20934652	-2.41354E-05	0.996386371
HES6 cg02105326	-0.017045081	2.15081E-06
HES6 cg08121041	-0.124217785	3.05072E-06
HES6 cg09163117	0.000369694	0.953250018
HES6 cg10210789	-0.008580307	0.066904668
HES6 cg10976861	-0.014983564	0.315124018
HES6 cg11027325	0.00491986	0.449798915
HES6 cg11815105	-0.002425839	0.385820552
HES6 cg15319457	-0.002662008	0.563654145
HES6 cg17283117	-0.002713949	0.530560697
HES6 cg23969423	-0.071696065	0.00169117
HES6 cg24127874	-0.005523039	0.497199076

HINT1 cg01175053	-0.004379336	0.831834383
HINT1 cg07158495	-0.002441207	0.804926473
HINT1 cg08523029	0.006409117	0.379571741
HINT1 cg10627136	-0.002232534	0.670564176
HINT1 cg12810560	0.000524434	0.921537877
HINT1 cg14645481	-0.001442553	0.809713742
HINT1 cg15686437	-0.002018422	0.584569373
HINT1 cg20640983	-0.001029917	0.917594856
HINT1 cg24936032	-0.003798271	0.45774032
HINT1 cg26967385	-0.039709641	0.058375217
HINT2 cg01328164	-0.001920077	0.750204634
HINT2 cg13565152	-0.002952015	0.531513061
HINT2 cg13931999	-0.007980949	0.388456845
HINT2 cg27490193	-0.003086746	0.511456998
HIST1H4C cg03376565	0.001072759	0.780913193
HIST1H4C cg11190156	-0.002022829	0.807904756
HIST1H4C cg12089985	-0.001711137	0.77541858
HIST1H4C cg14648265	-0.003897451	0.458740118
HIST1H4C cg15855450	-0.007373852	0.262850449
HIST1H4C cg18269503	-0.000740192	0.968884346
HIST1H4C cg18364779	0.000841558	0.951967335
HIST1H4C cg24453353	0.001832548	0.626761555
HJURP cg00688330	-0.000320547	0.987106344
HJURP cg01835144	-0.00063711	0.905191129
HJURP cg01983548	-0.030861604	0.073236904
HJURP cg02598797	-0.007835836	0.007060219
HJURP cg04550103	-0.060675638	0.086063851
HJURP cg05734866	-0.005474133	0.677918616
HJURP cg06085544	-0.007033309	0.119436024
HJURP cg06310422	0.000796121	0.908617914
HJURP cg07549195	-0.010876646	0.42322789
HJURP cg08010337	-0.006884094	0.218008038
HJURP cg12802413	-0.012990839	0.275030875
HJURP cg13260726	-0.001021579	0.957388673
HJURP cg14664879	-0.003165688	0.613856369
HJURP cg16639766	-0.000799015	0.809383935
HJURP cg17084697	-0.004122784	0.753852869
HJURP cg17919570	-0.005295323	0.118272143
HJURP cg18553762	-5.74997E-05	0.99437381
HJURP cg21133613	-0.003966376	0.260934102
HJURP cg21542780	0.001790936	0.678554471
HJURP cg21602955	-0.000183404	0.956326822
HJURP cg24074913	-0.002273414	0.934208035
HJURP cg27143307	-0.005685519	0.445882869
HMGB2 cg00321931	0.000297709	0.959031565
HMGB2 cg02666901	-0.001418937	0.627236503
HMGB2 cg03002428	-0.000122115	0.979878674
HMGB2 cg03124231	0.000707536	0.906307723
HMGB2 cg05944369	0.003181609	0.743703528

HMGB2 cg06841464	-0.003995862	0.286180062
HMGB2 cg08269316	0.000770156	0.899692975
HMGB2 cg09162056	-0.003567258	0.535239415
HMGB2 cg11186122	6.11317E-05	0.992124928
HMGB2 cg12571493	-0.043838495	0.09362734
HMGB2 cg13515674	0.000677161	0.917596647
HMGB2 cg14705778	0.006917541	0.42637993
HMGB2 cg16569664	0.000328948	0.968852347
HMGB2 cg19091677	-0.000115567	0.98534806
HMGB2 cg19371349	0.005482583	0.810712651
HMGB2 cg20409358	-0.003845436	0.384139681
HMGB2 cg21499459	-0.000322098	0.972801371
HMGB2 cg23199053	-0.006687923	0.098295396
HMGB2 cg23467443	-0.000103576	0.982257443
HMGB2 cg23860479	-0.000404068	0.940759768
HMGB2 cg24448859	0.002656538	0.751466864
HMGB2 cg24551570	0.002004411	0.92512927
HMGB2 cg25086702	0.000404104	0.947438699
HMGB2 cg27164753	0.002516485	0.828840287
HOPX cg00493422	-0.042412686	6.4884E-07
HOPX cg04085076	-0.059960887	1.81103E-05
HOPX cg04307083	0.026607273	0.520069103
HOPX cg04754315	-0.013546348	0.689071481
HOPX cg06202778	-0.003731836	0.802194375
HOPX cg06771126	-0.066775695	4.29201E-05
HOPX cg09853371	-0.015790751	0.201009004
HOPX cg10800833	-0.010626896	0.384731334
HOPX cg12791492	-0.004333503	0.458189508
HOPX cg13939978	-0.042225025	0.033449687
HOPX cg14196225	-0.008249575	0.609958311
HOPX cg16936060	-0.090739939	0.015219973
HOPX cg16975863	-0.08553128	4.43805E-10
HOPX cg19673329	-0.119904305	8.87449E-06
HOPX cg19978674	-0.042843686	0.282129232
HOPX cg21899596	-0.004526007	0.856254544
HOPX cg24852548	-0.008774706	0.620986402
HOPX cg25456368	-0.05198879	0.022281665
HOPX cg25948037	-0.058111991	0.09131948
HOPX cg27305383	-0.020469787	0.427416863
ITGB1BP1 cg00024812	0.000956058	0.646975781
ITGB1BP1 cg00522231	-0.008508882	0.785419832
ITGB1BP1	-0.001062505	0.79537251
ITGB1BP1 cg07814910	-0.002325021	0.603728285
ITGB1BP1 cg07974891	-0.149001763	7.19173E-10
ITGB1BP1 cg08100159	-0.03054094	0.07390772
ITGB1BP1 cg08937729	-0.005669533	0.284440311
ITGB1BP1 cg09734418	0.006593918	0.849821757
ITGB1BP1 cg09761705	0.002026119	0.781199657
ITGB1BP1 cg09949845	-0.002386757	0.318798032

ITGB1BP1 cg20093808	0.000332179	0.927987565
ITGB1BP1 cg20361001	-0.00104382	0.86603679
ITGB1BP1 cg23889771	-0.000998446	0.753726749
ITGB1BP1 cg24873957	-0.004671204	0.336990766
ITGB1BP1 cg26306976	-0.035193739	0.165099241
KCNQ1OT1 cg01744331	-0.069504562	0.009700171
KCNQ1OT1 cg01873334	0.000486619	0.986311971
KCNQ1OT1 cg01893176	0.006054216	0.841072211
KCNQ1OT1 cg02219360	0.003311901	0.920162106
KCNQ1OT1 cg02798157	0.001827719	0.947522264
KCNQ1OT1 cg03401726	-0.010019039	0.568005071
KCNQ1OT1 cg03422070	0.001735026	0.960917419
KCNQ1OT1 cg03654058	-0.047636832	0.032558574
KCNQ1OT1 cg04762676	-0.014022766	0.000504286
KCNQ1OT1 cg05740879	0.017040572	0.506522574
KCNQ1OT1 cg05816130	0.008083894	0.795239998
KCNQ1OT1 cg06809295	-0.053811322	2.5177E-05
KCNQ1OT1 cg07595203	0.003180732	0.911398519
KCNQ1OT1 cg08359167	0.003802253	0.804049772
KCNQ1OT1 cg08446215	0.009879592	0.753258553
KCNQ1OT1 cg09518720	0.01531385	0.381700205
KCNQ1OT1 cg11272547	-0.081690698	0.001393903
KCNQ1OT1 cg11666921	0.001769054	0.939708668
KCNQ1OT1 cg12077660	-0.003837404	0.856611782
KCNQ1OT1 cg12433386	-0.03060689	0.247650799
KCNQ1OT1 cg14243741	0.005645118	0.851398003
KCNQ1OT1 cg14958441	0.016508633	0.484911931
KCNQ1OT1 cg15651941	-0.009748912	0.708615593
KCNQ1OT1 cg16556677	-0.083646461	0.001273695
KCNQ1OT1 cg16739686	0.009383892	0.738851994
KCNQ1OT1 cg23906872	-0.033392208	0.023539049
KCNQ1OT1 cg25306939	0.00559026	0.855738089
KCNQ1OT1 cg26094482	0.003054905	0.897965407
KCNQ1OT1 cg26104781	0.005523104	0.866593233
KCNQ1OT1 cg26963277	-0.075840893	0.002246912
KCNQ1OT1 cg27323091	0.010084546	0.538855979
KIAA0101 cg00223963	-0.001626052	0.976367413
KIAA0101 cg02065795	-0.000386547	0.950834206
KIAA0101 cg03849685	0.001169212	0.742003091
KIAA0101 cg05877740	0.001189346	0.816026615
KIAA0101 cg08128680	0.002200507	0.337015602
KIAA0101 cg09214993	0.002395793	0.587735418
KIAA0101 cg11741168	-0.002032281	0.738822866
KIAA0101 cg19839691	0.002083723	0.509255551
KIAA0101 cg22679412	0.003864698	0.941989337
KIAA0101 cg23386807	0.00168291	0.707219749
KIAA0101 cg26155739	0.002594247	0.908204708
KIAA0101 cg26889367	0.003578422	0.29294593
KIF23 cg00126739	0.00186902	0.499313453

KIF23 cg01906442	0.000259895	0.963239905
KIF23 cg02714566	-0.002998411	0.432763152
KIF23 cg05717833	0.005685141	0.333658348
KIF23 cg05749577	-0.001837014	0.727245612
KIF23 cg06869971	-0.0044491	0.537035372
KIF23 cg08817171	-0.004985452	0.239906393
KIF23 cg15465548	-0.003040135	0.719723753
KIF23 cg15642412	-0.000686127	0.810342585
KIF23 cg16587794	-0.000166316	0.978489341
KIF23 cg22240424	0.001008047	0.894082911
KIF23 cg22412481	-0.037547176	0.009360687
KIF23 cg24170947	-0.003170127	0.508996468
KIF23 cg24657687	-0.00046663	0.910572035
KIF23 cg25142045	-0.099364916	0.000139539
KIF23 cg27514624	-0.003313813	0.795052958
KIF2C cg00148881	0.000370161	0.949677777
KIF2C cg00251536	6.88165E-06	0.999390307
KIF2C cg00455333	-0.001271223	0.725506558
KIF2C cg02890457	0.00093304	0.828115039
KIF2C cg03331258	-0.001117301	0.841815351
KIF2C cg03907454	0.00635037	0.678221345
KIF2C cg06922130	0.000245061	0.962389637
KIF2C cg12269116	-0.000137276	0.969620959
KIF2C cg12276329	0.000395359	0.92789013
KIF2C cg17418643	-0.026694137	0.248970364
KIF2C cg20487572	-0.046089819	0.003924937
KIF2C cg24539339	-0.02213449	0.126385265
KPNA2 cg05771398	-0.00076638	0.928028015
KPNA2 cg06090053	-0.001782214	0.889189635
KPNA2 cg06390411	-0.002168134	0.767428775
KPNA2 cg09771049	-0.017759989	0.144635036
KPNA2 cg13777502	0.008044904	0.703977811
KPNA2 cg14732136	-0.002764945	0.870598657
KPNA2 cg14898140	-0.003236354	0.809455843
KPNA2 cg16501440	0.000274177	0.961340771
KPNA2 cg17985418	-0.004528325	0.594061779
KPNA2 cg21018429	-0.00119423	0.846644646
KPNA2 cg21820889	-0.000494957	0.93244441
KPNA2 cg22429852	-0.007540669	0.88715083
KPNA2 cg23206777	-0.003744652	0.560317863
LMNB1 cg01077085	7.17814E-05	0.98044301
LMNB1 cg04284063	-0.071198544	0.013655051
LMNB1 cg05484879	-0.003724087	0.216029254
LMNB1 cg06405317	-0.025719352	0.018659909
LMNB1 cg08568504	-0.003394878	0.775098713
LMNB1 cg10204478	-0.005310561	0.822080074
LMNB1 cg14064399	0.002604856	0.443999185
LMNB1 cg16089416	-0.007575079	0.153088135
LMNB1 cg17248495	-0.000540835	0.942604341

LMNB1 cg18262958	0.000641253	0.781598877
LMNB1 cg20986134	-0.002060651	0.592033336
LMNB1 cg22007501	-0.00671855	0.066647488
LMNB1 cg22183605	-0.003845646	0.432791525
LMNB1 cg23953133	-0.001964193	0.837719857
LMNB1 cg25022925	0.00390194	0.743876232
LMNB1 cg26611836	-0.001763869	0.750445986
LLRN1 cg00742851	-0.099596643	1.95492E-11
LLRN1 cg01300546	-0.006655769	0.091340124
LLRN1 cg01442531	-0.160582213	2.3731E-08
LLRN1 cg02276274	0.027393276	0.161546288
LLRN1 cg03280839	0.003198299	0.769820055
LLRN1 cg03930929	-0.079445623	0.01054894
LLRN1 cg04150219	-0.032674905	0.024519119
LLRN1 cg06152533	0.004443486	0.820568482
LLRN1 cg08556718	-0.167519097	2.9307E-05
LLRN1 cg08844848	0.000751626	0.956621377
LLRN1 cg09615224	-0.003809298	0.32268864
LLRN1 cg09623845	-0.124394747	8.61331E-10
LLRN1 cg09665338	-0.144458343	4.25389E-06
LLRN1 cg10507275	-0.001787409	0.839718073
LLRN1 cg12136608	0.001791202	0.958258926
LLRN1 cg14784497	-0.007518266	0.12783209
LLRN1 cg15482530	-0.004719338	0.21414784
LLRN1 cg15999997	-0.05879329	0.001870511
LLRN1 cg17199247	0.003231953	0.746144121
LLRN1 cg17792833	-0.025560871	0.001061406
LLRN1 cg17912112	-0.006293736	0.66666557
LLRN1 cg18739166	-0.108588804	7.54621E-10
LLRN1 cg20438207	-0.088386551	0.00347707
LLRN1 cg22595235	-0.005809623	0.181471961
LLRN1 cg23238483	-0.16370328	7.17894E-09
LLRN1 cg23479065	-0.12524713	6.052E-09
LLRN1 cg25095722	-0.000653622	0.936945814
LLRN1 cg26163699	-0.001198607	0.853297711
LLRN1 cg27570309	-0.129099997	4.0723E-05
LSMD1 cg06935501	3.54126E-05	0.994654996
LSMD1 cg07402456	0.007139905	0.316399053
LSMD1 cg07666210	-0.008379728	0.487440024
MAD2L1 cg00680768	-0.001316952	0.941256262
MAD2L1 cg01131147	0.000110022	0.972044388
MAD2L1 cg03799581	-0.031448503	0.427298206
MAD2L1 cg05233515	-0.004949556	0.458854676
MAD2L1 cg05985659	-0.000239925	0.949220738
MAD2L1 cg06918925	-0.00129169	0.632294911
MAD2L1 cg16225753	0.000844045	0.814081471
MAD2L1 cg21262733	-0.002157603	0.713195192
MAD2L1 cg23781003	0.001161913	0.717796261
MAD2L1 cg27080119	0.00041172	0.957560719

MAD2L1 cg27094117	0.001031442	0.760061007
MALAT1 cg01185801	-0.002046191	0.509890147
MALAT1 cg02652361	-0.052600923	2.14146E-05
MALAT1 cg02943285	0.00237447	0.535756778
MALAT1 cg04073608	0.001788435	0.672692132
MALAT1 cg04378603	-0.004377353	0.614330116
MALAT1 cg04868132	0.002079697	0.446218762
MALAT1 cg04977124	0.007689193	0.623878347
MALAT1 cg05491695	0.007037535	0.656494648
MALAT1 cg07799005	0.000431663	0.945228351
MALAT1 cg10631284	-0.000738355	0.924857709
MALAT1 cg12498916	-0.000205074	0.988698067
MALAT1 cg14690315	0.002779575	0.463600876
MALAT1 cg15574972	0.000262289	0.987026915
MALAT1 cg17153055	-0.000311664	0.958912085
MALAT1 cg18501142	-0.003234184	0.682620346
MALAT1 cg19878733	0.001127106	0.879523065
MALAT1 cg20503416	-0.00319373	0.544166016
MALAT1 cg22311458	-0.006761525	0.296792722
MALAT1 cg23566411	-0.002241422	0.889794207
MALAT1 cg26489875	-0.002145877	0.699989406
MANF cg01044394	-0.006726269	0.281881268
MANF cg02225143	-0.004338731	0.401629893
MANF cg02255184	-0.002040416	0.787274253
MANF cg02264238	-0.032262057	0.114923895
MANF cg04007931	-0.006817053	0.316973236
MANF cg07118985	-0.011393915	0.138123088
MANF cg08058672	0.024586924	0.369193717
MANF cg08667777	-0.00642124	0.129630227
MANF cg10029076	0.014531823	0.374566453
MANF cg15881088	-0.003105456	0.341431459
MANF cg18639405	-0.004011916	0.527382225
MANF cg18861151	-0.003765408	0.471794598
MANF cg20936475	-0.000825856	0.904012772
MANF cg21034633	0.00451616	0.729923611
MANF cg22477501	-0.002766114	0.589807254
MANF cg24295551	-0.002262252	0.743151697
MARCKSL cg00292107	-0.069833539	3.2119E-10
MARCKSL cg00556029	-0.01516101	0.075424
MARCKSL cg02455803	-0.001552383	0.783830933
MARCKSL cg04348222	0.049052882	0.038537152
MARCKSL cg06221946	0.022660538	0.191059049
MARCKSL cg07092593	-0.000898203	0.78435531
MARCKSL cg09072560	-0.024159641	0.028149435
MARCKSL cg09327628	-0.0042605	0.158604429
MARCKSL cg22710219	-0.005766683	0.251814152
MEA1 cg00857557	-0.001559338	0.850708849
MEA1 cg03535439	-0.000200795	0.962846635
MEA1 cg04616717	0.001158683	0.894964959

MEA1 cg06802599	0.000879968	0.79972174
MEA1 cg10599035	-0.000994778	0.790483024
MEA1 cg13397365	0.000922101	0.85665554
MEA1 cg13871588	0.000677224	0.798941981
MEA1 cg15057823	0.001337456	0.787259231
MEA1 cg15329277	-0.027492483	0.327745612
MEA1 cg16044674	-0.007048382	0.360664865
MEA1 cg20235337	0.001408998	0.853914472
MEA1 cg20494108	-0.003137169	0.735502266
MEA1 cg21910650	-0.026544375	0.651256229
MEA1 cg23661578	-0.009542307	0.41582334
MFNG cg01765930	-0.027818102	0.564178576
MFNG cg03737999	0.005599404	0.35184206
MFNG cg11201532	-0.037322167	0.417312759
MFNG cg17066620	0.001710007	0.781826434
MFNG cg17809471	-0.000501655	0.93105067
MFNG cg18452324	0.000202747	0.989740375
MFNG cg22406469	-0.022537085	0.515918652
MFNG cg25597784	-0.021194629	0.587163839
MKI67 cg02707638	0.002168932	0.379147055
MKI67 cg04074793	0.002198835	0.675934621
MKI67 cg06442750	-0.0016622	0.939595842
MKI67 cg07066918	0.006410471	0.789154449
MKI67 cg07314148	-0.010062652	0.643667737
MKI67 cg13188795	-0.023389943	0.360238919
MKI67 cg17295447	-0.09799901	0.00011853
MKI67 cg17850838	-0.003108746	0.470073539
MKI67 cg17929627	0.001343378	0.792614545
MKI67 cg18691055	0.002755548	0.840731586
MKI67 cg18785410	0.000310486	0.937941997
MKI67 cg26235537	0.007434171	0.623391348
MPRL37 cg04095833		
MPRL37 cg08622517		
MPRL37 cg10535847		
MPRL37 cg19019972		
MT2A cg01190915	0.001640663	0.845614121
MT2A cg01910484	0.002069722	0.751456172
MT2A cg06663317	0.12297381	0.000263221
MT2A cg07395075	-0.008444609	0.232896971
MT2A cg08409642	0.141986153	2.4116E-07
MT2A cg16737422	-0.001547887	0.678668444
MT2A cg17886959	0.064305184	0.033103473
MT2A cg23581183	0.006761576	0.83229494
MXD3 cg02693857	-0.001859981	0.579323826
MXD3 cg04586126	0.066461913	0.000307481
MXD3 cg07671752	0.026701671	0.033889939
MXD3 cg08076617	-0.002812555	0.803172896
MXD3 cg09044296	0.01236174	0.286630801
MXD3 cg09564133	-0.006989549	0.140944894

MXD3 cg10533277	-0.000373612	0.929968103
MXD3 cg13278795	0.003459271	0.418880884
MXD3 cg16616449	0.004131374	0.724318573
MXD3 cg18465082	0.039350126	0.104967761
MXD3 cg25101061	-0.001752915	0.710308229
MXD3 cg25213720	0.066561174	0.00079112
MXD3 cg25368647	-0.09683877	5.14996E-08
MXD3 cg26087598	-0.002236768	0.569107522
MYC cg00163372	-0.012950891	0.571536782
MYC cg00611675	0.004922809	0.572952495
MYC cg01052743	-0.000523319	0.941497881
MYC cg01203549	-0.000971456	0.834393845
MYC cg03003858	0.002409917	0.696391674
MYC cg03076047	-0.002037599	0.739476708
MYC cg03498895	-0.005705299	0.586962099
MYC cg05927173	-0.001051698	0.896891493
MYC cg07871324	0.001908216	0.647984594
MYC cg08428512	-0.001368579	0.789561253
MYC cg08526705	-0.019455959	0.312681075
MYC cg10302505	0.000872743	0.871631903
MYC cg10340053	-3.71064E-05	0.994431335
MYC cg10832291	0.000906202	0.96622384
MYC cg10836034	-0.000157415	0.972664814
MYC cg11688275	0.001139704	0.902699014
MYC cg12057615	-0.000638394	0.906168855
MYC cg13244804	-0.003445888	0.735902803
MYC cg14732699	-6.33117E-05	0.997248388
MYC cg15465092	-0.003465938	0.580975843
MYC cg15801573	-0.008291166	0.410239535
MYC cg16100135	0.001893517	0.699925329
MYC cg17160660	-0.011653633	0.081272169
MYC cg17299732	-0.007637958	0.533110976
MYC cg17505251	-0.000998366	0.774412845
MYC cg19972619	0.001313858	0.849020339
MYC cg21803052	0.004007645	0.1248498
MYC cg22331096	0.001679044	0.812229475
MYC cg23187103	-0.000169599	0.984475599
MYC cg24666276	0.001991114	0.849825372
MYC cg25080152	0.006315789	0.337716088
MYC cg25826331	-0.000417898	0.945773939
MYC cg26221243	0.001791112	0.645889829
MYC cg26441142	-0.000145069	0.972423294
MYC cg27045396	-0.00320235	0.83557214
MYC cg27207274	-0.002608937	0.502731984
NCAPG cg02810967	-0.050255082	2.6384E-06
NCAPG cg03509813	-0.002357897	0.712458049
NCAPG cg03604819	-0.003463118	0.454155049
NCAPG cg06034885	-0.000843729	0.946945464
NCAPG cg06549407	0.005809896	0.722345178

NCAPG cg10333701	0.001225648	0.867505322
NCAPG cg11390445	-0.002644749	0.299406804
NCAPG cg13111374	-0.00105534	0.843282903
NCAPG cg14377370	-0.002734107	0.540008464
NCAPG cg21454030	-0.000419743	0.980166833
NCAPG cg22496254	-0.023350148	0.0290401
NCAPG cg23435440	-0.00366807	0.751154605
NCAPG cg26071746	-0.001378188	0.75438093
NDC80 cg00277996	0.000245048	0.979544256
NDC80 cg02807146	-0.002476671	0.59043445
NDC80 cg05407825	-6.55116E-05	0.99119581
NDC80 cg06454226	-0.093144145	4.0975E-10
NDC80 cg07333223	-0.001164919	0.75849833
NDC80 cg13425515	-0.000297173	0.952330254
NDC80 cg14313868	-0.000674131	0.886366056
NDC80 cg16421612	0.00630277	0.034401367
NDC80 cg16932672	-0.001106053	0.819400168
NDC80 cg17241550	0.002127521	0.79957583
NDC80 cg26393983	-0.000188875	0.959234701
NDC80 cg26661543	-0.000132768	0.970592433
NDUFA4 cg01145709	-0.002272	0.752247184
NDUFA4 cg01945793	-0.002621223	0.500969017
NDUFA4 cg02076804	0.002112871	0.265270567
NDUFA4 cg05143012	-0.019527975	0.523154821
NDUFA4 cg05715003	0.001755901	0.401812521
NDUFA4 cg08929390	-0.005209451	0.477418148
NDUFA4 cg10938970	-0.002549967	0.301740115
NDUFA4 cg10940374	0.002179341	0.592397088
NDUFA4 cg14972755	0.003047336	0.367491317
NDUFA4 cg16837769	0.00067278	0.768257896
NDUFA4 cg17268726	-0.010240243	0.207243961
NDUFA4 cg17695010	0.002160524	0.331938122
NDUFA4 cg21053748	-0.000311871	0.932848979
NDUFA4 cg23711939	-0.003736933	0.325587798
NDUFA6 cg00395296	0.002224214	0.936083493
NDUFA6 cg01529207	0.002706442	0.952748574
NDUFA6 cg02050560	0.002141238	0.77547194
NDUFA6 cg02882774	4.53E-05	0.994419384
NDUFA6 cg03805410	-0.000491609	0.886499199
NDUFA6 cg04607975	-0.002875835	0.441268193
NDUFA6 cg04764416	0.002416978	0.568064231
NDUFA6 cg07601932	-0.00458747	0.299126363
NDUFA6 cg07890336	0.00016586	0.986213559
NDUFA6 cg07987587	-0.004937766	0.74836287
NDUFA6 cg08957018	-0.003168313	0.774744968
NDUFA6 cg10846032	-0.003466443	0.343128718
NDUFA6 cg11189039	-0.001682386	0.771122214
NDUFA6 cg16085276	-0.001567901	0.664589313
NDUFA6 cg21025089	-0.002265226	0.753582613

NDUFA6 cg22183448	0.00786833	0.598764069
NDUFS8 cg05463966	0.087471407	0.000216733
NDUFS8 cg05753046	-0.046056769	0.073764776
NDUFS8 cg07693657	-0.005664542	0.624504224
NDUFS8 cg08139833	-0.002537696	0.43613243
NDUFS8 cg12070987	0.098906262	9.35699E-10
NDUFS8 cg14943827	-0.00217583	0.882608723
NDUFS8 cg16546622	-0.005276474	0.508906445
NDUFS8 cg16906595	0.000906117	0.827740714
NDUFS8 cg17583008	-0.007304007	0.295321227
NDUFS8 cg18386131	-0.001489831	0.831144795
NDUFS8 cg20906345	0.000103387	0.995342515
NDUFS8 cg22247282	0.003021566	0.797482205
NDUFS8 cg22951283	-0.003251198	0.574291485
NDUFS8 cg23962826	-0.002018933	0.811533039
NDUFS8 cg24713080	-0.000710004	0.946614666
NDUFS8 cg24757160	0.04790433	0.000117466
NDUFS8 cg26209546	0.000274863	0.958675367
NDUFS8 cg26406131	0.030014418	0.369433476
NEK2 cg07170222	0.001823289	0.773467095
NEK2 cg09897338	-0.001382356	0.841232229
NEK2 cg11225435	-0.002125205	0.543167564
NEK2 cg15831905	0.016088649	0.614505107
NEK2 cg17241013	-9.567E-05	0.991122051
NEK2 cg17931972	-0.109066801	0.000110681
NEK2 cg21395147	0.00155873	0.898290862
NEK2 cg24458466	0.017362361	0.140315932
NEK2 cg26036067	-0.001001447	0.87667941
NEU4 cg01987333	-0.083817918	0.00058308
NEU4 cg03372905	-0.154743225	7.21069E-07
NEU4 cg03877418	-0.014682339	0.095154627
NEU4 cg05195017	0.132602197	1.34703E-10
NEU4 cg08180831	-0.15724828	6.26677E-09
NEU4 cg10504150	-0.10091717	0.000116195
NEU4 cg11452267	-0.096753883	4.30895E-11
NEU4 cg11706790	-0.109229494	0.001177418
NEU4 cg15576082	-0.197214131	5.51872E-11
NEU4 cg15746851	-0.05370513	0.000109412
NEU4 cg16660312	-0.131010815	1.6842E-05
NEU4 cg17280514	0.002247411	0.965694935
NEU4 cg17696563	-0.025679078	0.041231182
NEU4 cg17981927	-0.06254452	0.000456876
NEU4 cg18095041	-0.134534006	2.22444E-06
NEU4 cg18485872	-0.080015667	0.00055928
NEU4 cg19007167	-0.104299643	0.001522375
NEU4 cg20052509	0.005823522	0.59050397
NEU4 cg20189808	-0.052495446	0.018756342
NEU4 cg20791593	-0.097996485	2.95247E-10
NEU4 cg21570020	0.017696672	0.017861511

NEU4 cg22399646	-0.060948687	0.005640416
NEU4 cg24429037	-0.141053102	0.000107796
NEU4 cg24629571	0.002731714	0.919022124
NEU4 cg26034168	-0.087577938	3.66178E-08
NMB cg03822754	0.007650248	0.562012443
NMB cg12220058	-0.030860587	0.00115068
NMB cg12863693	-0.050507505	0.017588937
NMB cg13893221	-0.001089376	0.929734033
NMB cg17173187	-0.031318207	0.003829213
NMB cg17891194	-0.001699193	0.485165236
NMB cg18582073	-0.004521155	0.547632328
NMB cg19517291	-0.001873814	0.952205123
NMB cg19997245	0.005668451	0.783194829
NMB cg20915763	-0.063121306	0.005727583
NMB cg24134342	0.035922019	0.105708186
NMB cg25653336	-0.010703569	0.681904498
NMB cg26576155	0.004593902	0.641826875
NMB cg27032142	-0.002710666	0.316399387
NMB cg27379587	0.007529062	0.492576056
NMB cg27417717	0.008075154	0.157478527
NUF2 cg01964683	-0.002436515	0.705500156
NUF2 cg09936566	-0.00061568	0.878721012
NUF2 cg11823214	-0.004814587	0.486816665
NUF2 cg15778350	-0.016068405	0.101987762
NUF2 cg16604136	-0.002182397	0.811632297
NUF2 cg17428694	-0.000172898	0.981951019
NUF2 cg21934237	-0.001727299	0.667762014
NUF2 cg21959457	0.038922121	0.384150504
NUF2 cg26029744	-0.001042955	0.765152518
NUF2 cg27042667	-0.021694198	0.000602666
NUSAP1 cg02417622	0.003214303	0.596447912
NUSAP1 cg07501391	-0.001314472	0.863261617
NUSAP1 cg07883696	0.002005953	0.865842032
NUSAP1 cg08782897	0.003259833	0.268473112
NUSAP1 cg11324740	-0.00044951	0.906292958
NUSAP1 cg12822975	-0.001514837	0.533501307
NUSAP1 cg13345248	0.000395718	0.941719706
NUSAP1 cg13503364	-0.00116839	0.832822249
NUSAP1 cg14581502	0.000676038	0.875624009
NUSAP1 cg16711321	0.006186692	0.070668388
NUSAP1 cg19105821	0.000164865	0.976369715
NUSAP1 cg21071862	-0.000870854	0.808779899
NUSAP1 cg21284844	-0.002567235	0.322392281
NUSAP1 cg24696207	-0.00500943	0.500857516
NUSAP1 cg25217313	0.001228955	0.8457816
NUSAP1 cg25861327	0.001149948	0.783513695
NUSAP1 cg26516489	0.00229254	0.612474557
NUSAP1 cg27127142	0.005757435	0.563934478
OLIG1 cg02348151	-0.012120482	0.599749074

OLIG1 cg02736969	-0.003774609	0.26111608
OLIG1 cg03732762	-0.000392342	0.951500223
OLIG1 cg03867475	-0.000138862	0.986430348
OLIG1 cg04351905	-0.030640866	0.429063574
OLIG1 cg05442477	-0.095947494	9.64586E-08
OLIG1 cg05522774	-0.009639401	0.16860039
OLIG1 cg05720454	-0.00328798	0.459456102
OLIG1 cg06106763	-0.007010512	0.405490589
OLIG1 cg06570025	0.006956636	0.5647678
OLIG1 cg15893431	-0.029135181	0.204381575
OLIG1 cg17016394	-0.005947516	0.787071931
OLIG1 cg20340508	-0.005539826	0.866846705
OLIG1 cg21569006	-0.006348667	0.234478356
OLIG1 cg26770917	-0.000742142	0.827709943
OLIG1 cg27237300	-0.009038438	0.78611191
OLIG1 cg27280904	-0.000776099	0.867604246
PBK cg00410990	-0.000867616	0.834274383
PBK cg01579019	0.001410672	0.848600491
PBK cg06816054	-0.01971191	0.002073326
PBK cg11026430	-0.001472272	0.788563255
PBK cg12863373	-0.003301171	0.248681357
PBK cg17795291	0.005011596	0.771889433
PBK cg17973773	-0.072789581	0.026643035
PBK cg21177558	0.000716479	0.929487015
PBK cg21210376	-0.036635438	0.028566032
PBK cg21734487	-0.00181652	0.649137649
PBK cg21887430	-0.070164508	0.002319623
PBK cg26261431	-0.014155909	0.124560804
PBK cg27310938	0.000999976	0.89649667
PCOLCE cg01197156	-0.129488241	1.19909E-05
PCOLCE cg01706943	0.007297885	0.90329752
PCOLCE cg02797569	-0.052229901	0.124047543
PCOLCE cg04522932	-0.017904909	0.236397006
PCOLCE cg05920870	-0.022605089	0.004943847
PCOLCE cg06330722	-6.70907E-05	0.992243849
PCOLCE cg09326362	-0.00427192	0.334793508
PCOLCE cg13655570	-0.004300753	0.159887463
PCOLCE cg14047091	-0.002864011	0.930002378
PCOLCE cg18178595	-0.041680397	0.025700759
PCOLCE cg19685521	-0.022059794	0.54883864
PCOLCE cg21684654	-0.03016818	0.083029613
PCOLCE cg22082800	-0.046793554	0.120260996
PCOLCE cg25680486	-0.075125965	0.002726884
PCOLCE cg26100986	-0.052578531	0.007200204
PCOLCE cg26122963	-0.075940868	0.00230628
PCOLCE cg26777475	-0.04051138	0.370101271
PCOLCE cg27069566	-0.047885987	0.168211375
PFN1 cg00581295	0.00080089	0.923748186
PFN1 cg06308537	0.003935365	0.827618851

PFN1 cg06554928	-0.002448247	0.465504688
PFN1 cg07465344	-0.017716873	0.619549103
PFN1 cg10240449	-0.004374691	0.669048292
PFN1 cg13491759	-0.004303678	0.377213354
PFN1 cg16889556	-0.003875099	0.317113015
PFN1 cg19752117	-0.00078886	0.890814393
PFN1 cg22114827	-0.000519942	0.932674256
PHF19 cg00045753	0.004752551	0.84598671
PHF19 cg02388253	-0.086928669	0.007494271
PHF19 cg03808351	0.085864661	0.000110183
PHF19 cg05834805	0.047524631	0.10298891
PHF19 cg11054680	-0.008235963	0.422050413
PHF19 cg11594303	0.000702538	0.817671977
PHF19 cg13419692	-0.066216183	0.09342377
PHF19 cg13501527	-0.093611536	0.00715588
PHF19 cg13793166	0.001208268	0.955709199
PHF19 cg14218513	-0.004119081	0.296814265
PHF19 cg14425045	0.02880781	2.29039E-05
PHF19 cg14543508	0.117569962	3.99575E-05
PHF19 cg14614490	-0.042483235	0.065994395
PHF19 cg18224492	-0.008288362	0.584698722
PHF19 cg21179713	0.00553125	0.649375171
PHPT1 cg07432438	-0.003723654	0.211178416
PHPT1 cg11185291	-0.09980129	1.97391E-08
PHPT1 cg11453712	0.091400095	0.000345683
PHPT1 cg13639672	-0.001025168	0.798088655
PHPT1 cg13789711	-0.003275908	0.349039466
PHPT1 cg14162552	-0.09313154	3.9229E-09
PHPT1 cg14245947	0.067098169	0.037738147
PHPT1 cg14290445	-0.095098692	1.49041E-09
PHPT1 cg16777839	0.000426245	0.939366137
PHPT1 cg17963089	-0.002024919	0.49256369
PHPT1 cg27152619	-0.003579007	0.515501009
POL2RI cg01166180	-0.003562909	0.213167025
POL2RI cg05031283	0.002048525	0.732384669
POL2RI cg07634150	0.000436256	0.947679599
POL2RI cg12960866	-0.007031812	0.14594924
POL2RI cg13597949	-0.007358199	0.17380891
POL2RI cg19720917	-0.000523387	0.96440634
POL2RI cg22746529	-0.043073981	0.050799547
POL2RI cg25230235	-0.001733039	0.572985349
POL2RI cg26681081		
PPIA cg03041365	-0.005746627	0.665039691
PPIA cg06436997	-0.008572248	0.05985705
PPIA cg08300117	-0.003686572	0.846533896
PPIA cg08901752	-0.001309651	0.502474881
PPIA cg11056045	0.003307893	0.809989812
PPIA cg12111272	-0.001390399	0.585997822
PPIA cg12342027	-0.003524051	0.851889052

PPIA cg12520111	-0.004792947	0.565510823
PPIA cg14056620	-0.005473368	0.269691926
PPIA cg22024117	-0.005690944	0.024621722
PPIA cg25980157	-0.001768459	0.675661252
PPIA cg26194633	0.002423082	0.822710484
PPIA cg26307266	-0.00350834	0.403105815
PRC1 cg01213445	0.001973146	0.886208602
PRC1 cg01407062	-0.09571187	1.43983E-05
PRC1 cg03379200	-0.00080231	0.915626227
PRC1 cg04509607	-0.007780053	0.293434612
PRC1 cg05564953	-0.000945651	0.934426149
PRC1 cg06613755	0.001131336	0.889794207
PRC1 cg09180926	-0.001467136	0.833602227
PRC1 cg12467864	-0.000575444	0.834174682
PRC1 cg12488346	-0.002130637	0.728878479
PRC1 cg14398957	-0.069435842	0.028796887
PRC1 cg14921757	-0.004684107	0.769916209
PRC1 cg15595742	-0.002416426	0.570752609
PRC1 cg15878949	-0.00748588	0.557556487
PRC1 cg23887623	-0.008548859	0.432070892
PRC1 cg24095889	-0.002549874	0.826773224
PRC1 cg25237056	-0.002948206	0.351869138
PRC1 cg25699121	0.000753014	0.953784985
PRC1 cg26181196	-0.004947834	0.759108585
PRR24 cg00581979	-0.007692817	0.713841582
PRR24 cg02861966	0.001667653	0.795225205
PRR24 cg05161930	-0.001305357	0.927924268
PRR24 cg06563149	0.005775326	0.699181531
PRR24 cg09260634	-0.045476927	2.76043E-06
PRR24 cg11239220	-0.001744841	0.873703849
PRR24 cg11324538	-0.042680829	0.000960966
PRR24 cg11557938	0.001517689	0.863160255
PRR24 cg12111432	-0.001276567	0.863227032
PRR24 cg16033376	0.003818691	0.56291242
PRR24 cg17398851	-0.004710473	0.696565478
PRR24 cg17460200	-0.000159226	0.972965054
PRR24 cg20642610	-0.002448853	0.646595279
PRR24 cg25253217	-0.002745045	0.813297884
PSMA7 cg00071265	-0.026689836	0.005129348
PSMA7 cg01423077	-0.004761902	0.347984811
PSMA7 cg03416888	0.000103439	0.982175029
PSMA7 cg04928513	-0.002327855	0.686535221
PSMA7 cg07864765	-0.001396098	0.848308751
PSMA7 cg08575399	-0.006715363	0.201827362
PSMA7 cg08833432	-0.033122018	0.007456342
PSMA7 cg11012980	-0.002146582	0.782881676
PSMA7 cg12800803	-0.011473367	0.080225699
PSMA7 cg13251669	-0.001421906	0.673052798
PSMA7 cg13475699	-0.004095643	0.141316144

PSMA7 cg15580043	0.002982139	0.693007947
PSMA7 cg17665883	-0.07688481	0.000265645
PSMA7 cg21803754	-0.002319916	0.560702522
PSMA7 cg24738006	-0.003011904	0.364533881
PSMB3 cg01970017	0.006660201	0.296476721
PSMB3 cg03255265	0.00220154	0.803213353
PSMB3 cg03394764	-0.000213095	0.979120386
PSMB3 cg04409945	0.002264622	0.363009554
PSMB3 cg06027620	-0.001642343	0.963330082
PSMB3 cg07456797	0.000336282	0.968028697
PSMB3 cg11349328	0.003402077	0.501268724
PSMB3 cg13671121	0.006667595	0.563818399
PSMB3 cg13824734	0.001301643	0.601459381
PSMB3 cg16545499	0.001386975	0.851485124
PSMB3 cg17215234	0.000488573	0.922034715
PSMB3 cg22348431	-0.000132112	0.977809585
PSMB3 cg24866651	-0.000145889	0.982893142
PSMB3 cg27434072	-0.002726495	0.640763302
PSME2 cg00730794	-0.001960838	0.510705111
PSME2 cg00962125	0.001965214	0.889349887
PSME2 cg05341425	-0.003345124	0.662196871
PSME2 cg05487736	-0.004491155	0.158569153
PSME2 cg10297165	0.001683233	0.739363885
PSME2 cg10769343	0.001341655	0.887362049
PSME2 cg12590010	-0.003259057	0.405282841
PSME2 cg13545642	-0.003145618	0.874019795
PSME2 cg21235874	0.002269462	0.620132983
PSME2 cg23381116	-0.003952714	0.315514282
PSME2 cg23525241	-0.002743504	0.732446347
PTN cg00653387	-0.00779363	0.001169717
PTN cg01751605	-0.004305499	0.454432267
PTN cg02701024	-0.040182321	0.346816994
PTN cg03464514	-0.004326692	0.578188483
PTN cg03749900	-0.002436931	0.868846958
PTN cg06052255	0.012086583	0.567389641
PTN cg06147210	-0.070808867	0.000497978
PTN cg06644721	0.012018475	0.087228545
PTN cg06915773	-0.001146188	0.751727318
PTN cg08991965	-0.058193392	0.033587269
PTN cg11521965	-0.00251003	0.661034041
PTN cg11874123	-0.000193607	0.954229459
PTN cg11991398	-0.049711124	0.128155439
PTN cg15284177	0.024242647	0.633332235
PTN cg16029260	-0.085535133	1.64489E-10
PTN cg18769353	0.002528002	0.610085132
PTN cg22979093	-0.006726401	0.356778869
PTN cg23787144	-0.004727371	0.852351471
PTN cg26979473	-0.003301068	0.795564527
PTTG cg00116688	9.02462E-05	0.987516216

PTTG cg09468767	-0.000554664	0.929672367
PTTG cg12430567	-0.010862051	0.040035978
PTTG cg15407784	-0.027966392	0.027556116
PTTG cg17367077	-0.088118004	0.015549302
PTTG cg19619065	0.000753471	0.949860885
PTTG cg21784134	-0.000938428	0.84271479
PTTG cg23024444	-0.043544315	0.004113073
PTTG cg26775866	-0.009352079	0.12289588
PTTG cg27185377	-0.004336613	0.466301047
PXMP2 cg00630583	-0.000289028	0.97202121
PXMP2 cg01226748	0.028387141	0.431490069
PXMP2 cg05508447	-0.001746433	0.73304293
PXMP2 cg08310608	-0.082135874	0.000324593
PXMP2 cg08829281	0.000727635	0.817061362
PXMP2 cg11855524	-0.101889875	0.001143824
PXMP2 cg12755061	-0.030237365	0.003826614
PXMP2 cg16246698	-0.006548794	0.5021923
PXMP2 cg18877514	-0.03747583	0.025080204
PXMP2 cg21894028	-0.020283495	0.142052674
PXMP2 cg26271840	-0.026539424	0.34111329
RACGAP1 cg05863443	0.000755775	0.868740266
RACGAP1 cg09631044	-0.000310604	0.964794757
RACGAP1 cg11079525	-0.00279611	0.466771778
RACGAP1 cg11203371	-0.000741283	0.909350187
RACGAP1 cg12556960	-4.80786E-05	0.996288361
RACGAP1 cg16210078	-0.058052214	0.047800125
RACGAP1 cg16614527	-0.058375003	0.03526995
RACGAP1 cg16935670	0.000285499	0.98852189
RACGAP1 cg17434273	0.000537634	0.896175444
RACGAP1 cg19339811	-0.001078783	0.725435706
RACGAP1 cg21161070	0.001340122	0.567447077
RACGAP1 cg21660921	0.000158452	0.945606931
RACGAP1 cg23517124	-0.000892135	0.787465997
RACGAP1 cg27159962	-0.097287823	0.00137822
RBX1 cg00585572	-0.007375808	0.129694894
RBX1 cg03160045	-0.008712821	0.05199747
RBX1 cg03614664	-0.002141557	0.573555935
RBX1 cg07288693	-0.000537315	0.975994961
RBX1 cg10781403	-0.002099431	0.602575252
RBX1 cg12338176	-0.034716557	0.084619948
RBX1 cg14789928	0.003859917	0.688547021
RBX1 cg19850545	-0.01072601	0.18286914
RBX1 cg21454656	-0.000517517	0.936588664
RDX cg00626438	-0.000302687	0.955301969
RDX cg01015879	4.89598E-05	0.988650324
RDX cg02243606	-0.002929394	0.939200427
RDX cg02535507	-0.004270209	0.243826373
RDX cg03141158	0.012962906	0.400090859
RDX cg05842391	-0.006056941	0.165670859

RDX cg08451637	-0.007290238	0.80531917
RDX cg08714586	-0.000996411	0.876498102
RDX cg09331704	-0.000749271	0.950778594
RDX cg09715738	-0.004537061	0.434736529
RDX cg11567172	-0.000619245	0.893477997
RDX cg15496005	-0.000603268	0.91725892
RDX cg15825059	0.040268952	0.246095455
RDX cg17015511	0.001353235	0.839541875
RDX cg19336191	-0.041759483	0.017949399
RDX cg23352579	-0.004306617	0.425097872
RDX cg24508561	-0.001807883	0.733051365
RDX cg25166381	-0.002622114	0.620339116
RDX cg25229114	-0.003375666	0.527034893
RDX cg25260114	-0.006201693	0.652457367
RDX cg26204042	-0.034284648	0.025481577
RRM2 cg00506866	-0.006167815	0.17872413
RRM2 cg02237186	-0.001308896	0.867921496
RRM2 cg04817183	-0.005035096	0.531305438
RRM2 cg05515713	0.000600769	0.855179009
RRM2 cg07545636	-0.000268138	0.962757853
RRM2 cg15345369	0.000121906	0.993322196
RRM2 cg17779456	0.000726974	0.943148374
RRM2 cg18623836	-0.046821053	0.041753232
RRM2 cg18639038	-5.17512E-05	0.987053976
RRM2 cg19516340	-0.034244126	0.005258017
RRM2 cg24419094	-0.117722479	0.004099107
RRM2 cg26095266	-0.004386487	0.69284761
RRM2 cg27637048	-0.037356338	0.02866202
SGOL1 cg02114449	-0.001459913	0.611894871
SGOL1 cg02921887	0.002439789	0.758763378
SGOL1 cg04951797	0.004702576	0.73820753
SGOL1 cg11494149	-0.004456842	0.209426312
SGOL1 cg12185908	0.000632938	0.919705263
SGOL1 cg12506980	0.002167238	0.650338756
SGOL1 cg12930269	-0.001682796	0.704382499
SGOL1 cg12933903	-0.033163325	0.001208155
SGOL1 cg14339353	0.001086341	0.870535433
SGOL1 cg16339185	-0.00122314	0.628408126
SGOL1 cg19823793	0.001085261	0.940927505
SGOL1 cg21395120	0.000714107	0.881545946
SGOL1 cg21745444	-0.002472661	0.59337859
SGOL1 cg21755440	0.000151684	0.979942691
SGOL2 cg22240704	0.015204746	0.540971716
SGOL2 cg22604940	0.000227758	0.971233364
SGOL2 cg05409597	-0.005130358	0.016197475
SGOL2 cg08404560	-0.001445401	0.576017865
SGOL2 cg09018151	-0.003336073	0.501616413
SGOL2 cg10630197	-0.002138938	0.905126074
SGOL2 cg13718278	-0.023994401	0.032141291

SGOL2 cg19235490	-0.001052632	0.844892458
SGOL2 cg24423567	-0.00243609	0.556480602
SGOL2 cg24930416	0.064192632	0.200698756
SGOL2 cg25338577	-0.004871073	0.068207147
SGOL2 cg26742320	0.023421542	0.037113758
SGOL2 cg26961824	-0.012137251	0.644596358
SGOL2 cg27400282	-0.002476605	0.890004953
SHD cg05891007	-0.015465611	0.078950539
SHD cg06065657	-0.008887397	0.034080712
SHD cg08852149	0.011774007	0.135483292
SHD cg14200834	-0.030150472	0.409557244
SHD cg18581445	-0.010907409	0.225025835
SHD cg23296369	-0.085539164	0.001780959
SHD cg24408057	-0.075539916	2.4252E-08
SHD cg24830664	-0.007722738	0.426306238
SHD cg26646370	-0.002909555	0.900342361
SHMT2 cg01267941	-0.002166658	0.590187683
SHMT2 cg01763913	-0.009213867	0.323103391
SHMT2 cg02773601	-0.001677592	0.788581702
SHMT2 cg03052162	0.068692361	0.009875545
SHMT2 cg06868473	0.055450003	0.057220821
SHMT2 cg08163918	-0.016688554	0.538140394
SHMT2 cg09215553	0.000192005	0.975892126
SHMT2 cg10952910	0.000843903	0.889706385
SHMT2 cg11350278	-0.006236902	0.498424268
SHMT2 cg11851129	-0.052886417	0.004918207
SHMT2 cg12502815	-0.004953497	0.500975568
SHMT2 cg12639933	-0.100566674	1.86223E-06
SHMT2 cg12988522	0.012405142	0.595818998
SHMT2 cg19535267	0.022812924	0.636525108
SHMT2 cg19621579	-0.000664401	0.916664821
SHMT2 cg21549333	-0.001229814	0.810073087
SHMT2 cg21697007	0.004585065	0.391195525
SHMT2 cg23588553	-0.001122147	0.891081326
SHMT2 cg25110782	-0.058076942	0.002222182
SHMT2 cg26220528	0.011178216	0.453696397
SHMT2 cg27039453	-0.010187551	0.238280958
SIVA1 cg02825646	2.25378E-05	0.997404289
SIVA1 cg08568075	-0.001844093	0.567083764
SIVA1 cg09315921	-0.000301433	0.964881229
SIVA1 cg10157936	-0.009635274	0.747793039
SIVA1 cg10285897	-0.001882321	0.659839597
SIVA1 cg13109045	-0.003575712	0.359869304
SIVA1 cg14043411	-0.000819457	0.836827982
SIVA1 cg14235723	-0.001338883	0.887687355
SIVA1 cg19495714	0.028434133	0.304765082
SIVA1 cg20257916	-0.000706654	0.890371001
SIVA1 cg22864355	-0.096397359	2.71545E-06
SIVA1 cg26953093	-0.00314696	0.716494407

SLC25A5 cg01325409		
SLC25A5 cg01497576		
SLC25A5 cg04643655		
SLC25A5 cg08411738		
SLC25A5 cg09456905		
SLC25A5 cg09625274		
SLC25A5 cg13223777		
SLC25A5 cg18242760		
SLC25A5 cg18585464		
SLC25A5 cg19409156		
SLC25A5 cg24125468		
SMC4 cg04212239	0.004066375	0.765948237
SMC4 cg04479354	0.001931353	0.794447709
SMC4 cg09100593	-0.039071974	0.240841578
SMC4 cg12785694	0.001065535	0.970010931
SMC4 cg13783238	0.015830468	0.680120638
SMC4 cg15174463	0.003827573	0.522874011
SMC4 cg16053434	0.003740398	0.481006992
SMC4 cg16978263	-0.001282687	0.818596926
SMC4 cg17472985	0.006123224	0.610916909
SMC4 cg17554896	-0.07893183	0.014917164
SMC4 cg21508214	-0.001150627	0.772552977
SMC4 cg21710313	0.002136785	0.544450931
SMC4 cg22668533	0.000515939	0.895309217
SMC4 cg25007351	0.002060766	0.595444328
SOCS3 cg01897823	-0.004035227	0.286287327
SOCS3 cg03752138	-0.056290619	0.003729487
SOCS3 cg04548563	0.012866231	0.061094463
SOCS3 cg05761277	-0.004217315	0.514574461
SOCS3 cg10279487	-0.042696328	0.340406135
SOCS3 cg10508317	-0.033505995	0.461780999
SOCS3 cg14721618	0.004796409	0.475787191
SOCS3 cg15502888	-0.007028528	0.184118101
SOCS3 cg18181703	0.000248965	0.995695141
SOCS3 cg19894975	-0.000695279	0.986319009
SOCS3 cg21500342	0.001711148	0.680072811
SOCS3 cg21985352	-0.001439099	0.89990726
SOCS3 cg22749855	0.055854264	0.043970203
SOCS3 cg23985214	-0.007146746	0.336154852
SOCS3 cg26998429	-0.000841962	0.89857009
SOCS3 cg27637521	-0.054856217	0.238836582
SOX4 cg00792966	-0.003317747	0.529651686
SOX4 cg02301209	-0.010270451	0.417110696
SOX4 cg03203320	-0.016097851	0.414278758
SOX4 cg05281544	-0.001482084	0.783627765
SOX4 cg06170053	0.000535503	0.933379058
SOX4 cg06512858	-0.002949361	0.610120475
SOX4 cg08625851	0.000108036	0.986642313
SOX4 cg08718230	-0.001104462	0.737058287

SOX4 cg08950757	0.000279207	0.960643182
SOX4 cg13995516	-0.006119653	0.443819753
SOX4 cg14499797	0.000391451	0.91510406
SOX4 cg22274825	0.000760528	0.920325229
SOX4 cg22671938	-0.003567867	0.25051621
SOX4 cg23148651	-0.010250212	0.300054297
SOX4 cg23345561	-0.004546312	0.77548768
SOX4 cg23780597	0.001141856	0.683618867
SOX4 cg24249751	-0.00266717	0.79348016
SPARC cg06571753	0.016948425	0.405622748
SPARC cg07539983	0.009833555	0.20287581
SPARC cg08331313	0.006983211	0.547550201
SPARC cg08879559	0.001187909	0.948395054
SPARC cg10505630	0.046144369	0.026581908
SPARC	0.023453285	0.378371954
SPARC cg21877464	0.037619975	0.099033459
SPARC cg22116670	0.008098379	0.542380954
SPARC cg23174201	0.009249012	0.769903638
SPARC cg25587069	0.01226501	0.43646145
SPARC cg25913233	-0.000495897	0.966907016
SPARC cg26389330	0.120265818	1.96782E-10
SPARC cg27128761	0.095516492	6.03983E-06
SPC25 cg04949346	-0.010509029	0.295258009
SPC25 cg05868191	-0.00680138	0.816136716
SPC25 cg06350524	0.003872141	0.535673466
SPC25 cg06580318	-0.001941733	0.729339846
SPC25 cg06971765	-0.00209817	0.82949881
SPC25 cg07224215	-0.001744885	0.847678222
SPC25 cg13605690	-0.002508198	0.348162036
SPC25 cg14465028	-0.000860548	0.890170959
SPC25 cg15237047	0.000832231	0.804506807
SPC25 cg17942426	-0.004688825	0.412153002
SPC25 cg20609092	-0.003583585	0.800946177
SPC25 cg22278106	-0.003810676	0.394364972
SSBP4 cg00751072	0.020942699	0.406136165
SSBP4 cg01450303	0.015287496	0.628639104
SSBP4 cg01532080	-0.012193623	0.400117474
SSBP4 cg07527607	-0.051147027	0.022528665
SSBP4 cg07561894	-0.0137502	0.23043797
SSBP4 cg09516349	-0.027233084	0.317859629
SSBP4 cg09529667	-0.000291205	0.991000797
SSBP4 cg10790698	-0.009817519	0.121546759
SSBP4 cg11778563	0.030775687	0.446243329
SSBP4 cg13331354	-0.007659584	0.571008278
SSBP4 cg18362448	0.015056065	0.626007041
SSBP4 cg18547371	0.003206277	0.796110202
SSBP4 cg20556517	-0.006572787	0.489234953
SSBP4 cg23544107	-0.061665028	0.0265316
SSBP4 cg25902939	-0.021986298	0.535949298

STK17A cg03345106	-0.002476171	0.520352598
STK17A cg05415936	-0.029336435	0.06430613
STK17A cg11022926	-0.056769345	4.02884E-07
STK17A cg14426688	-0.002541561	0.702257808
STK17A cg17568409	-0.002936729	0.473035038
STK17A cg17707057	0.047309111	0.070886862
STK17A cg18552620	-0.001510962	0.745824433
STK17A cg21309049	-0.000828784	0.832601433
STK17A cg22243438	-0.002885644	0.553407625
STK17A cg23213696	0.000319623	0.955116451
STK17A cg23281417	-0.000253973	0.949927604
STK17A cg23785719	0.002199311	0.827969174
TAGLN2 cg03138854	-0.002509391	0.340952047
TAGLN2 cg03651478	0.023628565	0.000331796
TAGLN2 cg04116858	-0.000180445	0.972047766
TAGLN2 cg04130057	8.54816E-05	0.987087946
TAGLN2 cg06992027	0.000444448	0.899381207
TAGLN2 cg08661227	-0.003310556	0.226839825
TAGLN2 cg13562386	0.007168584	0.107894381
TAGLN2 cg13892570	0.181616732	1.05798E-08
TAGLN2 cg14086160	0.003960394	0.464183046
TAGLN2 cg14633426	0.002868116	0.309457498
TAGLN2 cg15641364	0.076473039	0.005388275
TAGLN2 cg16107628	0.19708992	2.15679E-10
TAGLN2 cg18763326	0.0002488	0.937803477
TAGLN2 cg21571466	0.028011736	0.004872439
TAGLN2 cg22339338	0.007561217	0.862871593
TAGLN2 cg22698489	0.077742049	5.54162E-06
TAGLN2 cg26546310	-0.007885274	0.601611886
TCF12 cg00016066	0.00025161	0.975729232
TCF12 cg00027650	-0.047007001	0.003219629
TCF12 cg01778974	-0.051491483	0.006985117
TCF12 cg02734001	-0.108488327	4.12148E-07
TCF12 cg03237845	-0.043426564	0.143409504
TCF12 cg03334076	-0.02679156	0.000402586
TCF12 cg04858164	-0.067438395	0.043236877
TCF12 cg05141289	-0.009048542	0.683811166
TCF12 cg05359518	-0.082246432	0.000446428
TCF12 cg07046007	-0.003777442	0.566068861
TCF12 cg07276338	-0.055430442	0.004454318
TCF12 cg07769332	-0.00251231	0.640142517
TCF12 cg08128148	-0.021411525	0.085997083
TCF12 cg09989727	-0.125656989	1.42561E-06
TCF12 cg10146710	0.003330191	0.379113941
TCF12 cg11289748	-0.013934463	0.0156288
TCF12 cg12426141	-0.004437697	0.477493936
TCF12 cg12637615	-0.095602945	6.03576E-06
TCF12 cg13033086	0.007923324	0.663227263
TCF12 cg13165992	-0.032179063	0.021794762

TCF12 cg14303079	0.005711284	0.733275688
TCF12 cg14507132	-0.006046784	0.648295281
TCF12 cg15085879	-0.001674565	0.758331351
TCF12 cg16458838	-0.019541999	0.224550448
TCF12 cg16515457	-0.105685341	0.000663444
TCF12 cg17140034	-0.00421442	0.89378305
TCF12 cg17396978	-0.004427684	0.625191793
TCF12 cg17883068	0.06951655	0.06724018
TCF12 cg18117669	-0.045000254	0.009651302
TCF12 cg20555018	-0.042817133	0.002766715
TCF12 cg21831937	-0.111221918	0.000356867
TCF12 cg22677180	-0.005053422	0.741736218
TCF12 cg24079727	0.09098571	0.000653927
TCF12 cg24492778	0.09231839	2.39842E-06
TCF12 cg24783019	-0.029380572	0.023952049
TCF12 cg24784129	-0.089127163	0.00033813
TCF12 cg25005357	-0.131626829	1.25605E-08
TCF12 cg25688583	-0.035582894	0.049464811
TCF12 cg25707951	-0.070870985	0.053481617
TCF12 cg26810157	-0.05144906	0.198614371
TCF12 cg27119612	-0.114243922	2.25055E-11
TCTEX1D2 cg00961292	-0.005310097	0.750961973
TCTEX1D2 cg01915044	-9.17495E-05	0.976129682
TCTEX1D2 cg02026253	-0.006955443	0.097787335
TCTEX1D2 cg04887335	-0.018726701	0.274234093
TCTEX1D2 cg10336858	-0.006107869	0.559812952
TCTEX1D2 cg14465410	-0.023382649	0.354596659
TCTEX1D2 cg15268007	-0.001060051	0.732880131
TCTEX1D2 cg19600391	-0.000482962	0.938263798
TCTEX1D2 cg22505907	-0.048461628	0.039162609
TCTEX1D2 cg22624966	-0.005364519	0.38086105
TECR cg01542019	-0.053347889	6.17768E-06
TECR cg02644301	-0.001591312	0.530304186
TECR cg03162410	0.008673207	0.829611575
TECR cg03205495	-0.003284567	0.320608309
TECR cg04387237	-0.000626025	0.878994648
TECR cg06893491	-0.007668065	0.868461402
TECR cg08345979	-0.001345503	0.854942043
TECR cg08434594	-0.002092365	0.822406876
TECR cg10007692	-0.001836249	0.626665557
TECR cg13237829	0.001941892	0.620920638
TECR cg14339287	-0.002014582	0.770146742
TECR cg14898306	0.000132254	0.970975522
TECR cg15649193	-0.000854294	0.895374723
TECR cg15697902	-0.027676775	0.127744005
TECR cg15939937	-0.020607324	0.284734041
TECR cg16860821	0.003302685	0.922626025
TECR cg19263877	-0.010868024	0.019462249
TECR cg20630207	-0.006042411	0.450973668

TECR cg21511523	0.005015672	0.755373202
TECR cg23470272	-0.000232431	0.989656745
TECR cg23690893	0.013615943	0.669225435
TECR cg24143196	-0.093156921	0.0016585
TECR cg25685510	0.001286146	0.965534777
TK1 cg00715343	-0.020259293	0.014589528
TK1cg03291825	-0.016120323	0.688189135
TK1cg06098276	-0.090405141	0.00527884
TK1cg08115732	-0.107773914	9.81913E-09
TK1cg18767057	0.026200687	0.099786847
TK1cg20104688	0.000928909	0.824572655
TK1cg20740903	0.000780337	0.984833006
TK1cg21519872	-0.046391806	0.030479931
TK1cg25069807	-0.069749509	0.017842129
TK1cg26206461	-0.015096712	0.248538998
TMEM147 cg02035330	-0.001266886	0.825694531
TMEM147 cg05815685	0.000973966	0.939154027
TMEM147 cg08480906	-0.002647525	0.285971417
TMEM147 cg13869971	-0.001437063	0.775706911
TMEM147 cg19016517	-0.001775323	0.756619457
TMEM147 cg24080181	-0.002341937	0.362515323
TMEM54 cg06179485	-0.001294564	0.966831275
TMEM54 cg12598340	-0.02500381	0.444454094
TMEM54 cg15626665	-0.013060207	0.07657892
TMEM54 cg15999547	-0.025837167	0.033267018
TMEM54 cg17832212	-0.011983352	0.273051056
TNFRSF12A cg00510447	0.022112147	0.002092033
TNFRSF12A cg06097320	0.002804788	0.639740862
TNFRSF12A cg06209210	-0.000503523	0.939750298
TNFRSF12A cg08798492	0.008415637	0.502908795
TNFRSF12A cg15460516	0.002982906	0.670762198
TNFRSF12A cg20195987	0.000287644	0.95957928
TNFRSF12A cg26808293	0.007411615	0.255709844
TNK2 cg00190795	-0.061130224	2.54813E-06
TNK2 cg00359285	-0.048711405	0.004097883
TNK2 cg00514684	-0.005493314	0.604352193
TNK2 cg00544413	-0.00403224	0.444945453
TNK2 cg00651401	-0.011165611	0.692054494
TNK2 cg01006102	-0.106323158	3.68967E-10
TNK2 cg01766844	-0.006800507	0.68827686
TNK2 cg01792640	-0.040106261	0.041718847
TNK2 cg01962086	0.009081484	0.821193831
TNK2 cg02584969	0.000533409	0.948239105
TNK2 cg02633073	-0.000954543	0.835833665
TNK2 cg02920514	-0.02729491	0.000106188
TNK2 cg03207310	0.00022137	0.958670313
TNK2 cg03421964	-0.078177259	0.001225405
TNK2 cg04056669	-0.136411288	4.00681E-06
TNK2 cg04214520	-0.168729498	1.09713E-08

TNK2 cg04848502	-0.002929187	0.538874209
TNK2 cg05064121	0.000628642	0.953250018
TNK2 cg05802478	-0.031913608	0.045415113
TNK2 cg08048178	-0.007409729	0.751990649
TNK2 cg08119325	-0.080798979	2.1754E-07
TNK2 cg09459146	-0.007673814	0.293959213
TNK2 cg09628877	-0.137923154	1.77347E-07
TNK2 cg10542362	0.012810328	0.406682192
TNK2 cg10843210	-0.11729846	1.0641E-05
TNK2 cg11095099	-0.011070979	0.574101954
TNK2 cg11150667	-0.025096219	0.00343181
TNK2 cg14030878	-0.173524962	2.01481E-12
TNK2 cg15010544	-0.121592488	0.000101083
TNK2 cg15065340	0.041088773	0.179249469
TNK2 cg15854365	-0.168846021	2.04678E-13
TNK2 cg16047223	-0.006610901	0.036584579
TNK2 cg16229161	-0.188705417	1.47995E-08
TNK2 cg16568314	-0.001867909	0.897497339
TNK2 cg16615155	-0.036904665	0.006346653
TNK2 cg16929775	-0.178654346	7.01504E-08
TNK2 cg17640485	-0.046303367	3.85393E-05
TNK2 cg17663253	-0.000599098	0.946675374
TNK2 cg17913255	-0.033196849	0.07605596
TNK2 cg18473978	-0.190388755	9.58394E-10
TNK2 cg18716096	-0.004016162	0.327401421
TNK2 cg18783792	-0.146392072	2.68767E-08
TNK2 cg19955173	0.018990703	0.595171658
TNK2 cg20697427	-0.038118961	0.000255407
TNK2 cg21008828	-0.011264788	0.3841559
TNK2 cg21425003	-0.008578455	0.040694025
TNK2 cg21438160	-0.178383496	5.09006E-08
TNK2 cg21553700	-0.146573678	5.82696E-07
TNK2 cg21642245	-0.022854731	0.498581368
TNK2 cg22558522	-0.00760024	0.432008359
TNK2 cg22762745	-0.005794011	0.546728661
TNK2 cg22809023	-0.004398861	0.487658159
TNK2 cg23258717	-0.215520077	2.60362E-09
TNK2 cg23548201	-0.071821666	3.74144E-07
TNK2 cg25152312	-0.023244641	0.21347456
TNK2 cg26033513	-0.029460455	0.000149838
TNK2 cg26686927	-0.025775023	0.073977204
TNK2 cg27228383	-0.194731824	4.54764E-09
TNK2 cg27418204	-0.179627514	7.93651E-13
TOP2A cg03359285	-0.000672003	0.929632502
TOP2A cg05120042	-0.10138251	2.5625E-07
TOP2A cg07388903	-0.006378789	0.367121503
TOP2A cg08817965	0.005114994	0.362758221
TOP2A cg09273772	-0.007258098	0.226391671
TOP2A cg11393025	-0.002559838	0.573079574

TOP2A cg14572634	-0.005116706	0.631804697
TOP2A cg15769921	0.002515295	0.712396225
TOP2A cg15797971	-0.002138725	0.719153243
TOP2A cg17504397	-0.007435752	0.210600379
TOP2A cg18314885	-0.015910659	0.482184682
TOP2A cg18998002	-0.010223815	0.019185391
TOP2A cg21499137	-0.003732112	0.49566176
TOP2A cg22912359	0.002262465	0.708417322
TOP2A cg22935319	-0.009527393	0.716152695
TOP2A cg25450986	0.001999006	0.637807239
TOP2A cg25581784	-0.000567742	0.934216475
TOP2A cg26007540	-0.000270011	0.971999321
TOP2A cg26134124	-0.0013694	0.762735761
TOP2A cg26164164	-0.003281394	0.571369853
TPI1 cg01213014	-0.001707674	0.733434564
TPI1 cg01441359	0.001739447	0.476180988
TPI1 cg01607849	0.001095247	0.934799372
TPI1 cg03067226	-0.000838988	0.811596865
TPI1 cg03737815	0.001920024	0.466364648
TPI1 cg09357135	-0.000275248	0.948975372
TPI1 cg10471644	-0.002280429	0.481887744
TPI1 cg11021764	0.001639567	0.644489355
TPI1 cg15325042	-0.053028257	0.037242128
TPI1 cg16426148	0.00011043	0.990952236
TPI1 cg17184604	-0.006187525	0.394136373
TPI1 cg24555065	-0.003038606	0.184047202
TPI1 cg24672547	0.001906723	0.616815513
TPI1 cg26269324	-0.001375587	0.690209302
TPI1 cg26276120	-0.011565609	0.092281407
TPX2 cg02256410	-0.002343412	0.689561827
TPX2 cg03352975	-0.004472727	0.65512378
TPX2 cg09863659	0.001751269	0.777519834
TPX2 cg09877164	-0.000274423	0.931601603
TPX2 cg10476558	-0.00278693	0.531772763
TPX2 cg10480038	-0.001332406	0.731129004
TPX2 cg14326210	-0.008256771	0.405689556
TPX2 cg14878016	-0.000310429	0.931558288
TPX2 cg16338822	-0.000368536	0.933173313
TPX2 cg21838233	-0.000270194	0.94419613
TPX2 cg23807894	-0.001684879	0.933552529
TPX2 cg25649779	0.000112887	0.987236418
TPX2 cg26820641	0.001809107	0.673487897
TUBA1B cg00973724	-0.004478138	0.412792404
TUBA1B cg02084811	-0.001659346	0.693780426
TUBA1B cg02585102	0.000756878	0.955223489
TUBA1B cg07420135	-0.001419514	0.649529135
TUBA1B cg11220397	-0.004479151	0.659146748
TUBA1B cg13709639	-0.009671089	0.573518744
TUBA1B cg14217534	-0.001788119	0.535224182

TUBA1B cg14483935	-0.083949679	1.81404E-06
TUBA1B cg16975985	0.001446301	0.900647336
TUBA1B cg18239763	0.004098542	0.482810991
TUBA1B cg19676328	0.00188401	0.762737979
TUBA1B cg21300561	0.001730855	0.713841582
TUBA1C cg21681289	5.16347E-05	0.986739652
TUBA1C cg23702657	-0.001738267	0.736037263
TUBA1C cg01418856	-0.000392645	0.97673317
TUBA1C cg01581974	0.000457713	0.971342896
TUBA1C cg02269388	0.001729706	0.920619524
TUBA1C cg04797936	0.045345514	0.002372239
TUBA1C cg15929573	0.003679786	0.717935584
TUBA1C cg16881676	0.204447898	8.68423E-11
TUBA1C cg17359076	0.000126141	0.985980643
TUBA1C cg19426128	-0.001563018	0.962778771
TUBA1C cg24296900	-0.000981302	0.964930317
TUBA1C cg25751371	0.023711132	0.106947579
TUBA1C cg27632435	-0.006814897	0.641596559
TUBB2A cg00231644	-0.001711449	0.92594018
TUBB2A cg13626842	-0.004112912	0.12990116
TUBB2A cg14462067	-0.006899902	0.223846596
TUBB2A cg15173079	-0.047227837	0.095268292
TUBB2A cg18049569	-0.013729606	0.29936445
TUBB2A cg21102926	-0.002664386	0.380876292
TUBB2A cg21526654	-0.003514565	0.318345589
TUBB2A cg22943782	0.000878386	0.869640206
TUBB2A cg24306277	-0.000348022	0.910515624
TUBB2A cg25433222	-0.031532404	0.194624748
TUBB2A cg27530015	-0.008541466	0.285154204
TUBB6 cg03507241	0.011703165	0.862968078
TUBB6 cg07307078	0.032675157	0.521993328
TUBB6 cg12879381	-0.003983138	0.740076149
TUBB6 cg16546503	-0.014768794	0.820314603
TUBB6 cg16808455	0.008704528	0.839577624
TUBB6 cg18348731	-0.004203778	0.841901267
TUBB6 cg22467216	-0.004298219	0.933962681
TUBB6 cg25836232	-0.052327969	0.100726406
UBE2C cg00242976	-0.025542855	0.004671881
UBE2C cg01661001	0.003024313	0.790363732
UBE2C cg02405476	-0.00921177	0.079698802
UBE2C cg02838589	-0.021444871	0.483910617
UBE2C cg03843031	-0.002388054	0.553379205
UBE2C cg03969725	-0.061161075	2.69928E-06
UBE2C cg10985576	-0.006377151	0.507224609
UBE2C cg12354270	-0.002081119	0.545457154
UBE2C cg13736875	0.000828364	0.854473813
UBE2C cg13853156	-0.003097949	0.647118436
UBE2C cg18038749	-0.001959578	0.688238776
UBE2C cg19197419	-0.008451887	0.226464987

UBE2C cg19222480	-0.002507863	0.489625108
UBE2C cg20034785	-0.00358848	0.55925416
UBE2C cg22082347	0.000187043	0.976522034
UBE2C cg22431438	-0.000208584	0.969844954
UBE2C cg23265604	-0.002307152	0.707904808
UBE2C cg23873448	-0.001148107	0.66528623
UBE2C cg24675094	-0.000591442	0.854354851
UBE2T cg01918706	-0.002084691	0.665148867
UBE2T cg02926368	-0.003742681	0.327884078
UBE2T cg11819639	-0.0045249	0.326405863
UBE2T cg13395333	-0.000636952	0.93045077
UBE2T cg15173196	-0.003289048	0.417333337
UBE2T cg16993579	0.0022868	0.890038798
UBE2T cg26399396	-0.004033196	0.458303963
UQCC cg04149172	-0.026995035	0.267938881
UQCC cg05298461	0.000840924	0.814671214
UQCC cg05389922	-0.007961795	0.708525986
UQCC cg08152058	-0.00185359	0.617978269
UQCC cg11281845	-0.00085207	0.809949206
UQCC cg12277998	0.002145988	0.774518977
UQCC cg12522898	-0.002019637	0.713865902
UQCC cg14752227	0.001371985	0.97508001
UQCC cg17046996	-0.000737955	0.954632857
UQCC cg20564458	0.000968684	0.852057795
UQCC cg24906992	-0.002558769	0.459924047
UQCC cg26475306	-0.001338521	0.781376833
UQCR cg01094684	0.000412856	0.928771629
UQCR cg01608030	-0.008861061	0.198468357
UQCR cg04156970	-1.74506E-05	0.997868503
UQCR cg04600406	-0.005211219	0.368473335
UQCR cg05219517	-0.005164884	0.214261539
UQCR cg10643550	0.039072667	0.139605858
UQCR cg11792664	-0.001677648	0.800624949
UQCR cg12235988	0.001344259	0.708077801
UQCR cg15696376	0.000470725	0.887753194
UQCR cg15974216	-0.004168428	0.784311598
UQCR cg22625420	-0.002191811	0.68376972
UQCR cg26571977	-0.006740276	0.361853805
UQCR cg26885578	-0.003143719	0.376013028
VIM cg00146951	0.001749894	0.59666091
VIM cg01003015	0.011687476	0.386677624
VIM cg01154046	-0.000397914	0.952343447
VIM cg02236650	0.047275817	0.031720108
VIM cg02746869	-0.000574293	0.962457038
VIM cg05151811	0.000304423	0.970996454
VIM cg06460869	0.002416117	0.717523548
VIM cg08918274	0.006742789	0.043627408
VIM cg11973177	0.00077578	0.9525822
VIM cg12155165	0.002579614	0.620079135

VIM cg12874092	-0.000101914	0.971833113
VIM cg13414916	-0.000201	0.952370325
VIM cg14260889	0.111397557	0.006415222
VIM cg14898116	0.010436576	0.623114328
VIM cg15363487	-0.000165692	0.988112765
VIM cg18514820	0.008665773	0.122465337
VIM cg19111999	0.017864288	0.005360718
VIM cg19170009	0.088135829	0.009152949
VIM cg20018469	0.00575962	0.308580289
VIM cg20198108	0.002972843	0.681250104
VIM cg20319091	-5.14925E-05	0.992246665
VIM cg23821329	0.048213006	0.00026877
VIM cg23912429	-0.000985823	0.867548989
VIM cg23991622	-0.006518866	0.491787833
VIM cg26063719	7.17121E-05	0.996403506
VIM cg26306372	-0.000523198	0.953992565
VIM cg26983469	0.001128359	0.905054189
VIM cg27313572	0.001601114	0.956683829
ZWINT cg00371482	0.0024496	0.885996111
ZWINT cg01021053	3.20074E-05	0.997916419
ZWINT cg02286335	-0.012905767	0.443632811
ZWINT cg06313381	0.000189397	0.993950712
ZWINT cg12758503	-0.005411067	0.677925067
ZWINT cg14642833	-0.000731641	0.963150992
ZWINT cg14859667	0.0022267	0.919634981
ZWINT cg14993200	-0.001964228	0.885235131
ZWINT cg18242377	-0.000549772	0.971150137
ZWINT cg18534872	-0.005538531	0.777519834
ZWINT cg21206959	-0.00204426	0.869561745
ZWINT cg26022932	-0.001551537	0.930183379

ell, 2022)

Transsynaptic signaling signature refering to Fig. 2a, modified from Südhof (Neu		
**Gene with CpG site**	**FDC in high-neural glioblastoma**	**p-value**
RTN4R cg00384653	-0.163569391	1.94975E-10
RTN4R cg07543823	-0.002704419	0.473324917
RTN4R cg07159686	0.005083913	0.418604521
RTN4R cg25037841	-0.107083518	1.38228E-09
RTN4R cg22677723	-0.088826076	4.62418E-05
RTN4R cg16474791	-0.0378271	0.044526358
RTN4R cg17654470	-0.008344876	0.153435082
RTN4R cg08557686	-0.073962704	0.000658163
RTN4R cg07306604	0.01545919	0.344251146
RTN4R cg09784932	0.00253102	0.718310769
RTN4R cg12393104	-0.037681387	0.053041456
RTN4R cg03475420	-0.017258915	0.163350774
RTN4R cg06950987	-0.010577435	0.557488406
RTN4R cg13397314	-0.018913316	0.251056554
RTN4R cg18293555	-0.108672173	4.47977E-11
RTN4L1 cg14571092	-0.002093438	0.944499614
RTN4L1 cg15524101	-0.011296758	0.143492499
RTN4L1 cg08457620	-0.015623864	0.019664367
RTN4L1 cg26589485	-0.065101821	0.001004234
RTN4L1 cg04207549	-0.001436756	0.865173265
RTN4L1 cg17142404	-0.004694868	0.6250197
RTN4L1 cg12967327	-0.047972363	5.92049E-06
RTN4L1 cg27299033	0.024126915	0.298772106
RTN4L1 cg25813630	0.01434334	0.767419742
RTN4L1 cg21802919	-0.008764141	0.589586619
RTN4L1 cg05879505	-0.017007893	0.384833114
RTN4L1 cg05550893	0.003023605	0.676283177
RTN4L1 cg24948079	-0.00898084	0.828517749
RTN4L1 cg26365813	0.100362235	1.30704E-05
RTN4L1 cg08454053	-0.240042615	1.09841E-13
RTN4L1 cg23630423	-0.007328024	0.435703131
RTN4L1 cg20899146	-0.010714353	0.129490048
RTN4L1 cg27466709	-0.001080031	0.97263183
RTN4L1 cg01019770	0.000381157	0.950671923
RTN4L1 cg23669611	-0.075411963	0.001002527
RTN4L1 cg11251135	-0.004722523	0.692376084
RTN4L1 cg08197956	-0.083421339	0.001791909
RTN4L1 cg23094620	-0.001723671	0.954903472
RTN4L1 cg03214325	-0.150356281	1.12873E-06
RTN4L1 cg22791545	-0.048514263	0.024523234
RTN4L1 cg26699755	-0.001769774	0.900233057
RTN4L1 cg19550533	0.013206738	0.540624323
RTN4L1 cg06791321	-0.010481482	0.140859906

RTN4L1 cg07471768	-0.013004228	0.219802477
RTN4L1 cg14249528	-0.009079157	0.535249962
RTN4L1 cg18059383	-0.094528639	0.000122322
RTN4L1 cg14769786	0.017982035	0.699193461
RTN4L1 cg23289581	-0.106816381	0.000224799
RTN4L1 cg02064158	0.020129041	0.136267975
RTN4L1 cg04584833	-0.041154555	0.000793731
RTN4L1 cg26772766	0.048018877	0.111762575
RTN4L1 cg20641798	-0.021054591	0.256271616
RTN4L1 cg05861697	-0.031270109	0.124762307
RTN4L1 cg08875357	-0.031020027	0.343418755
RTN4L1 cg04743758	-0.095097305	0.000141019
RTN4L1 cg10479452	-0.02997131	0.067700991
RTN4L1 cg11398400	-0.006857137	0.392167209
RTN4L1 cg19619387	-0.080097754	2.83064E-05
RTN4L1 cg07061728	-0.006752758	0.847154062
RTN4L2 cg08924696	-0.004594952	0.671421409
RTN4L2 cg12528597	-0.006040687	0.797422824
RTN4L2 cg12822074	0.080823898	0.002126876
RTN4L2 cg08081323	0.027756014	0.395520151
RTN4L2 cg19956836	-0.053083516	0.175971984
RTN4L2 cg01932827	-0.031446302	0.017362312
RTN4L2 cg03280245	0.044349335	0.041109821
RTN4L2 cg25625670	-0.002563108	0.762382678
RTN4L2 cg01557260	0.007324478	0.773672231
RTN4L2 cg18048562	0.028800883	0.523313926
RTN4L2 cg21121024	-0.006934499	0.47380617
RTN4L2 cg17349753	-2.84478E-05	0.996811061
RTN4L2 cg24983959	-0.012058136	0.568722296
ODZ2 cg06873590	-0.022927018	0.681994829
ODZ2 cg26874611	0.002056249	0.972226343
ODZ2 cg09025649	-0.004839654	0.668681271
ODZ2 cg19775582	-0.093295667	0.031530343
ODZ2 cg19836145	-0.121834238	6.0882E-06
ODZ2 cg08463280	0.019283128	0.479267114
ODZ2 cg11097249	-0.051365481	0.324890101
ODZ2 cg19537558	-0.033834744	0.229780867
ODZ2 cg10647925	-0.011216846	0.449790807
ODZ2 cg08815365	-0.01620774	0.773970919
ODZ2 cg18013921	-0.015790813	0.148701424
ODZ2 cg01227558	-0.043684991	0.437050766
ODZ2 cg15380508	0.034596047	0.146069548
ODZ2 cg12659981	-0.06996702	0.162262678
ODZ2 cg01015395	-0.076952413	0.006894701
ODZ2 cg07705913	-0.04109201	0.025188218
ODZ2 cg06651505	-0.136946749	3.15466E-05
ODZ2 cg15090161	-0.068920102	0.002847193
ODZ2 cg06507590	-0.025017963	0.536019184
ODZ2 cg06793849	0.067128839	0.037684939

ODZ2 cg17567195	-0.110622403	6.19214E-07
ODZ2 cg13365822	-0.077882473	0.110539179
ODZ2 cg00045762	-0.011730005	0.462527886
ODZ2 cg19913582	-0.001041844	0.874322035
ODZ2 cg11107657	-0.060814399	0.001587983
ODZ2 cg18994744	-0.010618774	0.377190364
ODZ2 cg05600609	-0.102156609	0.002690667
ODZ2 cg11061434	-0.010008157	0.870990332
ODZ2 cg05030450	-0.037024634	0.162508638
ODZ2 cg09171112	-0.004019352	0.156362092
ODZ2 cg16531386	-0.062987076	0.012453339
ODZ2 cg18679069	-0.027889077	0.472496724
ODZ2 cg01881971	-0.051997843	0.006734446
ODZ2 cg14653988	-0.009926227	0.799137763
ODZ2 cg22816865	-0.173133378	7.61005E-07
ODZ2 cg18026062	-0.003136793	0.947274315
ODZ2 cg10882756	-0.025457917	0.620094265
ODZ2 cg24180759	-0.171529612	1.53196E-06
ODZ2 cg18287241	-0.098536683	7.13862E-06
ODZ2 cg09555364	0.014200431	0.17810381
ODZ2 cg22656750	-0.061691925	0.197257065
ODZ2 cg19984170	-0.044159103	0.102311761
ODZ2 cg05207048	-0.122909801	1.21089E-06
ODZ2 cg00615414	-0.095769283	0.053274621
ODZ2 cg26101465	0.001627117	0.76348652
ODZ2 cg11483884	0.025467358	0.55555043
ODZ2 cg23981116	-0.161184903	0.000195204
ODZ2 cg13983686	0.005408333	0.89805572
ODZ2 cg15651267	0.002898656	0.744528503
ODZ2 cg01897191	0.022729186	0.497792345
ODZ2 cg16658460	0.081411627	8.5633E-06
ODZ2 cg16656232	0.00766472	0.633818466
ODZ2 cg23072711	-0.04547731	0.28660498
ODZ2 cg17405646	-0.01186466	0.811086764
ODZ2 cg08985853	-0.011028165	0.827263583
ODZ2 cg10602135	-0.002031086	0.932179864
ODZ2 cg16434098	-0.077091191	0.011714837
ODZ2 cg11091196	-0.03915116	0.071065386
ODZ2 cg27022334	-0.027910606	0.228494043
ODZ2 cg01540953	-0.047819676	0.007694419
ODZ2 cg07141875	-0.05496698	0.001045566
ODZ2 cg22554488	-0.067388422	0.155896903
ODZ2 cg21188154	-0.03558129	0.065526967
ODZ2 cg02667656	0.088494341	8.11927E-10
ODZ2 cg18243017	0.006651407	0.565612026
ODZ2 cg04221388	0.017905731	0.00226857
ODZ2 cg07474269	0.002438465	0.734491614
ODZ2 cg16190746	-0.056485736	0.010939924
ODZ2 cg08577341	-0.006069406	0.71013585

ODZ2 cg17543425	-0.018011809	0.392372531
ODZ2 cg04001520	0.067086849	1.44614E-06
ODZ2 cg08769507	-0.011535711	0.124713376
ODZ2 cg00540540	-0.153254387	2.1143E-06
ODZ3 cg21746339	-0.002481606	0.710468893
ODZ3 cg22654504	-0.025820283	0.468837301
ODZ3 cg26850677	-0.029198169	0.50961043
ODZ3 cg25311162	-0.100082342	0.003189984
ODZ3 cg06366833	-0.138781399	5.76902E-10
ODZ3 cg19251291	-0.053413355	0.129765284
ODZ3 cg00767135	-0.009644639	0.28624443
ODZ3 cg01289782	-0.001175681	0.907457621
ODZ3 cg21618036	-0.10269999	0.008152765
ODZ3 cg21659014	-0.012866844	0.165261181
ODZ3 cg06589421	-0.047257186	0.124621609
ODZ3 cg16508529	0.015293899	0.610017809
ODZ3 cg09054951	-0.047542956	0.359789482
ODZ3 cg00517849	-0.020780759	0.204381575
ODZ3 cg11760342	-0.105530238	7.14454E-09
ODZ3 cg08182463	-0.023929854	0.092137719
ODZ3 cg17594004	-0.019651041	0.349786382
ODZ3 cg08174779	-0.045392104	0.006533217
ODZ3 cg03242448	0.001863214	0.870944435
ODZ3 cg10226257	-0.038681439	0.088343291
ODZ3 cg21165501	-0.095689341	0.003850862
ODZ3 cg01182076	-0.072091925	0.074905952
ODZ3 cg23625823	-0.033913139	0.532202373
ODZ3 cg26677675	-0.026113919	0.235562321
ODZ3 cg05342469	-0.036088472	0.260123484
ODZ3 cg16793274	-0.004997472	0.199522839
ODZ3 cg09378743	-0.010591545	0.662209395
ODZ3 cg00330059	0.008236539	0.423983583
ODZ3 cg19667211	-0.005456963	0.381047648
ODZ3 cg11778362	-0.035035004	0.168847776
ODZ3 cg01950474	-0.006191225	0.898152489
ODZ3 cg22878084	-0.04042589	0.150299464
ODZ3 cg13131878	-0.018241152	0.421072522
ODZ3 cg23344136	-0.055387222	0.074126785
ODZ3 cg05673204	-6.43949E-05	0.996912409
ODZ3 cg14981034	-0.075316413	0.072848907
ODZ3 cg14231974	-0.00077899	0.845035902
ODZ3 cg25979373	-0.074982371	0.015115758
ODZ3 cg05690069	-0.028245543	0.393792906
ODZ3 cg08810664	-0.00563498	0.620556813
ODZ3 cg10992201	-0.032736675	0.070952048
ODZ3 cg04973839	-0.033377563	0.313280733
ODZ3 cg08802511	-0.000812957	0.935649851
ODZ3 cg01546472	-0.002749565	0.837661228
ODZ3 cg23824376	-0.017805322	0.399600077

ODZ3 cg04774696	-0.083383449	0.000202705
ODZ3 cg16548818	-0.014747977	0.704259008
ODZ3 cg13322601	-0.00801397	0.692940842
ODZ3 cg06527816	-0.003978843	0.849478092
ODZ3 cg19111338	-0.118846006	1.98411E-05
ODZ3 cg22051345	-0.011519325	0.104507927
ODZ3 cg04090412	-0.17227541	5.34006E-06
ODZ3 cg10136736	0.001160036	0.932016066
ODZ3 cg09298466	-0.141718583	0.000308349
ODZ3 cg21122439	-0.022094688	0.237171589
ODZ3 cg02422426	-0.019412847	0.348922423
ODZ3 cg19082230	-0.068335215	0.001398762
ODZ3 cg17790852	-0.022505946	0.403347726
ODZ3 cg16690577	-0.128674073	2.36557E-05
ODZ3 cg01802453	-0.020737144	0.620907756
ODZ3 cg09767602	-0.051301227	0.115941926
ODZ4 cg17996830	-0.174467115	8.87293E-07
ODZ4 cg08734703	0.027381435	0.208865077
ODZ4 cg12245084	0.042855183	0.311944685
ODZ4 cg14565764	-0.003314432	0.317821611
ODZ4 cg15006118	-0.007984886	0.233733
ODZ4 cg03599147	-0.010042163	0.261122808
ODZ4 cg26707845	0.000136643	0.970400324
ODZ4 cg19234412	0.014613801	0.586871304
ODZ4 cg25198661	0.003572233	0.544899783
ODZ4 cg13787610	-0.013573539	0.429819259
ODZ4 cg23361764	-0.109268494	6.10121E-06
ODZ4 cg08683012	-0.000722669	0.927288976
ODZ4 cg13493005	-0.022612705	0.279429862
ODZ4 cg09419192	0.026839773	0.171553586
ODZ4 cg22284860	-0.018089644	0.489092839
ODZ4 cg08580087	-0.063269492	0.040247153
ODZ4 cg04649598	0.001793771	0.75164744
ODZ4 cg18777792	-0.003585407	0.203758352
ODZ4 cg24810917	-0.000478284	0.910540938
ODZ4 cg17395738	0.004821915	0.61341036
ODZ4 cg03825175	0.070909795	0.02834512
ODZ4 cg15678386	-0.008474528	0.256026153
ODZ4 cg15137643	-0.004460355	0.78877299
ODZ4 cg06959283	-0.008601173	0.006873275
ODZ4 cg12830752	-0.003755428	0.895552313
ODZ4 cg11097902	0.001099819	0.878248142
ODZ4 cg04212127	-0.00148377	0.959965768
ODZ4 cg10365900	-0.014741401	0.08917661
ODZ4 cg21094613	-0.007669688	0.201536383
ODZ4 cg10775595	-0.044933381	0.061351147
ODZ4 cg03177972	0.000508372	0.985243082
ODZ4 cg18222192	0.056480947	0.00029437
ODZ4 cg07233109	-0.049714341	0.009092353

ODZ4 cg01665408	-0.022091507	0.023818567
ODZ4 cg25487430	-0.049801212	0.025869304
ODZ4 cg20831648	-0.018012234	0.360942269
ODZ4 cg11251728	-0.109742468	0.001423842
ODZ4 cg01943983	-0.012981991	0.044783509
ODZ4 cg12851789	-0.056835433	0.007720495
ODZ4 cg09143850	-0.003565321	0.396299116
ODZ4 cg12643458	0.162343827	4.75678E-12
ODZ4 cg15110243	-0.00565151	0.73913435
ODZ4 cg07441953	7.35109E-05	0.99840531
ODZ4 cg13242944	-0.018534006	0.257632676
ODZ4 cg07340447	-0.128338064	0.000702066
ODZ4 cg06562283	-0.002863464	0.908575246
ODZ4 cg26703880	-0.012613947	0.725072507
ODZ4 cg09286493	-0.059668768	0.037636124
ODZ4 cg23982226	-0.036767693	0.001802118
ODZ4 cg08794954	-0.019164874	0.012041012
ODZ4 cg12986485	0.017871723	0.256343652
ODZ4 cg18224993	-0.092313843	1.08484E-09
ODZ4 cg21765235	-0.003133995	0.602861783
ODZ4 cg05797821	-0.001254244	0.876558327
ODZ4 cg10933002	-0.01896796	0.389077716
ODZ4 cg27214378	-0.006459874	0.294509295
ODZ4 cg14417468	-0.010869054	0.262004215
ODZ4 cg22987614	-0.095698327	1.83237E-05
ODZ4 cg12642289	-0.005678078	0.053564859
ODZ4 cg11172483	-0.024915923	0.001703175
ODZ4 cg26879899	-0.006958631	0.171189135
ODZ4 cg15310583	0.001578133	0.974711777
ODZ4 cg24860429	-0.002416099	0.676037468
ODZ4 cg24070541	-0.017422161	0.061262966
ODZ4 cg12051260	-0.00318661	0.779430496
ODZ4 cg17146918	-0.008228593	0.456468454
ODZ4 cg06251348	0.02919436	0.000987703
ODZ4 cg15714223	3.34092E-05	0.99881666
ODZ4 cg10030504	-0.172918694	4.14833E-06
ODZ4 cg06330691	-0.029255161	0.107817602
ODZ4 cg13062618	0.026035781	0.009156543
ODZ4 cg12025243	-0.010912948	0.123589368
ODZ4 cg14762722	-0.006258004	0.04664325
ODZ4 cg24277007	-0.002054952	0.939520049
ODZ4 cg00366359	-0.045879917	0.003594975
ODZ4 cg19884965	-0.003347852	0.863563678
ODZ4 cg19407682	-0.038478048	0.016213238
ODZ4 cg10640961	-0.021015459	0.252597997
ODZ4 cg25965355	-0.013675882	0.044470732
ODZ4 cg02760300	0.046801958	2.24329E-06
ODZ4 cg15765817	-0.001537398	0.776306521
ODZ4 cg09966895	-0.002103486	0.828415537

ODZ4 cg02594183	-0.035043577	0.050640878
ODZ4 cg12162138	-0.03711496	0.159234319
ODZ4 cg13228701	0.034979954	0.028119591
ODZ4 cg16235582	-0.019709886	0.022062241
ODZ4 cg26430023	-0.04715905	0.001367813
ODZ4 cg23279192	-0.071990148	0.004965821
ODZ4 cg02409108	-0.015532745	0.230850452
ODZ4 cg26977644	-0.003110833	0.557167295
ODZ4 cg15355859	-0.111376311	0.003598436
ODZ4 cg05172425	-0.046347346	0.009710933
ODZ4 cg05340269	-0.019464253	0.766765379
ODZ4 cg09673208	0.031428595	0.001671337
ODZ4 cg14309111	-0.0220523	0.199456574
ODZ4 cg03648711	-0.035156755	0.004530853
ODZ4 cg11862642	0.050563961	0.0206119
ODZ4 cg00908927	-0.003788523	0.679560867
ODZ4 cg20480334	-0.009794264	0.010131028
ODZ4 cg17542408	0.004751834	0.390240638
ODZ4 cg24208325	0.027250674	0.399048223
ODZ4 cg23956844	-0.00386574	0.839449305
ODZ4 cg08017233	-0.029036355	0.032796304
ODZ4 cg05218311	-0.069473328	4.26685E-08
ODZ4 cg12841273	-0.041102399	0.004149478
ODZ4 cg26452771	0.010924459	0.260060312
ODZ4 cg14098737	0.002327263	0.914465083
ODZ4 cg05099909	-0.006188143	0.631100141
ODZ4 cg03970849	0.035922129	0.249611712
ODZ4 cg11179180	-0.071995109	0.001116826
ODZ4 cg17579825	-0.002506921	0.42037745
ODZ4 cg11076902	-0.005304505	0.299891457
ODZ4 cg11002763	0.017401963	0.408643574
ODZ4 cg20482760	0.003400113	0.549347579
ODZ4 cg04983516	0.01585668	0.690500144
ODZ4 cg14294793	-0.024203723	0.514275412
ODZ4 cg12246510	-0.011830882	0.678240037
ODZ4 cg01149449	-0.06014721	0.167804332
ODZ4 cg08115371	-0.011023995	0.015379693
ODZ4 cg22782986	0.028784678	0.106235121
ODZ4 cg11968091	-0.008762329	0.208199581
ODZ4 cg22318139	0.012292806	0.136147631
ODZ4 cg15354625	0.119791732	2.2073E-06
ODZ4 cg25783326	-0.046768497	0.103861632
ODZ4 cg18431910	-0.053289724	0.203682184
ODZ4 cg19842216	-0.003476036	0.561043617
ODZ4 cg06892009	0.000371332	0.981950747
LPHN1 cg27499361	-0.000538833	0.985262115
LPHN1 cg19681357	-0.010947202	0.013636055
LPHN1 cg25726085	-0.027911937	0.249510377
LPHN1 cg24530000	-0.009333296	0.400844681

LPHN1 cg08173263	-0.016332269	0.540950535
LPHN1 cg15417798	0.002097411	0.971875999
LPHN1 cg04416414	0.001965502	0.603052507
LPHN1 cg18544450	-0.000354835	0.988164075
LPHN1 cg10722938	-0.01497432	0.094731337
LPHN1 cg00037314	-0.01373189	0.615374384
LPHN1 cg21048262	-0.015849442	0.197445694
LPHN1 cg22791904	-0.004912127	0.545928358
LPHN1 cg14565903	-0.001069317	0.889957006
LPHN2 cg04789392	-0.043858983	0.045056741
LPHN2 cg19627200	-0.009673066	0.580160502
LPHN2 cg22534621	-0.017513651	0.414800763
LPHN2 cg17083209	-0.011730057	0.143426923
LPHN2 cg19744908	0.00234989	0.841732377
LPHN2 cg07104652	-0.008086374	0.257703935
LPHN2 cg02996869	-0.006134529	0.34732595
LPHN2 cg02243785	-0.001380926	0.847146326
LPHN2 cg25974355	-0.080179351	0.011654619
LPHN2 cg22518696	0.005462166	0.704690738
LPHN2 cg17870792	0.003911936	0.704720382
LPHN2 cg00557709	-0.004056602	0.65582222
LPHN2 cg05125795	-0.003951647	0.440935715
LPHN2 cg07212035	-0.001486728	0.858706292
LPHN2 cg12130328	-0.001190048	0.851993411
LPHN2 cg13559233	-0.0096435	0.379383496
LPHN2 cg14562832	-0.008316738	0.401842921
LPHN2 cg04226724	-0.003079773	0.762685264
LPHN2 cg13980528	-0.002682814	0.512473464
LPHN2 cg20863963	0.000867855	0.964930317
LPHN2 cg16678153	-0.058975604	0.182702369
LPHN2 cg21783729	8.77294E-05	0.992878157
LPHN2 cg06940482	-0.103581215	0.00064545
LPHN2 cg14529474	-0.014491961	0.039508648
LPHN2 cg10992267	-0.001699144	0.835333082
LPHN3 cg06011964	-0.000616432	0.946671225
LPHN3 cg14382835	0.00996013	0.702532737
LPHN3 cg19339146	-0.073781669	0.004494027
LPHN3 cg16406385	-0.007109742	0.85235054
LPHN3 cg26281476	-0.005243061	0.707914715
LPHN3 cg18242400	-0.007129289	0.928653555
LPHN3 cg14974938	0.077016089	0.010972429
LPHN3 cg08572278	-0.113869596	0.000660513
LPHN3 cg12646794	-0.11545607	9.07708E-06
LPHN3 cg18416585	0.010181676	0.679327569
LPHN3 cg00681365	0.000997815	0.94544881
LPHN3 cg22671986	0.003374358	0.494086578
LPHN3 cg09163478	0.003705789	0.624779314
LPHN3 cg22479532	0.075512049	1.93096E-05
LPHN3 cg21814870	-0.128034686	7.15069E-05

LPHN3 cg06452184	-0.117906107	2.71694E-13
LPHN3 cg24221639	-0.052933378	0.0022359
LPHN3 cg09027155	0.009652454	0.813278317
LPHN3 cg04331368	0.004296608	0.856986501
LPHN3 cg06562406	-0.051400888	0.049807603
LPHN3 cg04118610	0.056792823	1.99965E-08
LPHN3 cg24087039	0.005137676	0.65234177
FLRT1 cg21111471	-0.092470542	2.01942E-07
FLRT1 cg18093064	0.005312318	0.455678372
FLRT1 cg09375205	-0.002308643	0.835284736
FLRT1 cg06268327	-0.018348736	0.29266931
FLRT1 cg17676132	-0.025376428	0.025221318
FLRT1 cg01256440	0.002461313	0.694355151
FLRT1 cg05016508	-0.011043728	0.037592288
FLRT1 cg08788438	-0.00612117	0.660133809
FLRT1 cg04740931	-0.006241513	0.773072401
FLRT1 cg13263274	-0.040437845	0.122575027
FLRT1 cg24431193	-0.000511954	0.970093584
FLRT1 cg15823423	0.001692869	0.79742368
FLRT1 cg13555362	-0.049417503	0.028611142
FLRT2 cg01832662	-0.105733015	8.47112E-05
FLRT2 cg18386876	-0.132733938	1.80208E-08
FLRT2 cg21227253	-0.003967727	0.869929312
FLRT2 cg15922990	-0.00168474	0.51494951
FLRT2 cg13232249	-0.005277889	0.158273411
FLRT2 cg23934295	-0.006215198	0.310917081
FLRT2 cg22859727	-0.011537603	0.830448096
FLRT2 cg22518208	-0.003980161	0.36334349
FLRT2 cg01537995	-0.001319866	0.921809089
FLRT2 cg24775496	-0.004402577	0.241957494
FLRT2 cg07166601	-0.001404018	0.872911568
FLRT2 cg16492417	-0.008342294	0.016133622
FLRT2 cg02805920	-0.004007814	0.143390871
FLRT2 cg05295297	0.002630018	0.841144433
FLRT2 cg13624964	0.001270889	0.82415814
FLRT2 cg18070676	-0.009876142	0.519050688
FLRT2 cg19622874	-0.002229057	0.524667495
FLRT2 cg13594869	-0.010933693	0.006460556
FLRT2 cg18098534	0.011116916	0.190453847
FLRT2 cg19687728	-0.000475901	0.942500559
FLRT2 cg02305242	-0.004035886	0.797164064
FLRT2 cg05855039	-0.003871232	0.386002356
FLRT2 cg04836612	-0.001572407	0.820015664
FLRT2 cg12155356	-0.000450792	0.926380963
FLRT2 cg16800165	-0.022628742	0.326632309
FLRT2 cg04435483	-0.097708334	9.32725E-05
FLRT2 cg17410236	-0.009960255	0.71630448
FLRT2 cg04862347	-0.015365906	0.007003075
FLRT2 cg05381290	0.003151545	0.697172436

FLRT2 cg18919660	-0.006743234	0.350799969
FLRT2 cg26235748	-0.024839591	0.046385579
FLRT2 cg04975505	-0.003638517	0.742443154
FLRT2 cg19953646	-0.01877001	0.367543859
FLRT2 cg01711160	-0.004789274	0.26984704
FLRT2 cg23000309	-0.001681436	0.496285136
FLRT2 cg02165099	-0.002226296	0.78441662
FLRT2 cg01485627	-0.006627379	0.403707702
FLRT2 cg12080751	-0.050310683	0.000813751
FLRT2 cg14125604	-0.001138929	0.859592371
FLRT2 cg10406690	-0.020206243	0.565865898
FLRT2 cg03506640	-0.001019619	0.838824048
FLRT2 cg14400498	-0.031020885	0.44577676
FLRT2 cg18845692	-0.00026234	0.994005762
FLRT2 cg02032642	-0.069194665	0.001425379
FLRT2 cg01919632	-0.006196186	0.303087168
FLRT2 cg25120290	-0.004319752	0.362850181
FLRT2 cg26651233	0.097372697	3.4711E-10
FLRT2 cg21571339	0.000193833	0.986924338
FLRT2 cg16324934	-0.033345555	3.25511E-06
FLRT3 cg17696268	-0.001063625	0.861766681
FLRT3 cg23127998	-0.003842822	0.393222883
FLRT3 cg10038446	0.064203047	0.013803621
FLRT3 cg26082838	-0.002519426	0.633167131
FLRT3 cg17752684	0.009496258	0.370961798
FLRT3 cg01642503	-0.006949229	0.052961359
FLRT3 cg06722503	-0.000332135	0.921663401
FLRT3 cg20886259	0.005044796	0.582777892
FLRT3 cg00471768	-0.030857318	0.344497811
CA10 cg05229989	-0.001729315	0.706451147
CA10 cg06557985	-0.000858145	0.781100075
CA10 cg03145062	-0.010253482	0.001654246
CA10 cg21385821	-0.001495079	0.665356204
CA10 cg07723251	-0.131685952	0.000125611
CA10 cg25592977	-0.027572476	0.112463793
CA10 cg14054928	-0.112970786	9.33887E-05
CA10 cg24341611	-0.107384612	0.00024312
CA10 cg20552747	0.0427345	0.243849494
CA10 cg13125157	0.005664535	0.834125575
CA10 cg06598197	-0.018905464	0.465055636
CA10 cg08605326	-0.020400601	0.552915295
CA10 cg20405017	-0.105790511	0.001611434
CA10 cg07398767	-0.00775376	0.825457631
CA10 cg22855255	-0.093691463	0.003488213
CA10 cg08034077	-0.005940466	0.89596393
CA10 cg14073722	0.015420073	0.772873172
CA10 cg26629942	0.045938656	0.176949828
CA10 cg04881814	-0.112805703	0.002654804
CA10 cg17208530	-0.050273452	0.254004298

CA10 cg14056110	-0.022396919	0.361503032
CA10 cg22702328	-0.027868175	0.284680704
CA10 cg25039722	0.000173719	0.996743519
CA10 cg07111868	-0.006344468	0.405834559
CA10 cg25694755	-0.008690856	0.641891908
CA10 cg17326372	0.00043519	0.995447628
CA10 cg25063515	-0.062617591	0.139179189
CA10 cg19805005	-0.067062249	0.089099788
CA10 cg09486367	0.013793464	0.711998223
CA10 cg07101732	-0.05599696	0.134834213
CA10 cg27504861	-0.064702293	0.034246539
CA10 cg11144436	-0.009125434	0.748090463
CA10 cg12691739	0.078741371	0.004439174
CA10 cg25499121	0.01101937	0.802034949
FAM19A1 cg03858703	-0.06189949	0.044805453
FAM19A1 cg22706186	-0.08916105	0.00106626
FAM19A1 cg02997497	-0.040062414	0.340363205
FAM19A1 cg01709518	-0.117669605	0.000167256
FAM19A1 cg03530364	-0.038533809	0.410909882
FAM19A1 cg12656851	-0.069501343	0.106162494
FAM19A1 cg09533390	-0.009376732	0.876370573
FAM19A1 cg13066983	-0.037774928	0.320186821
FAM19A1 cg09398550	-0.021512496	0.711081858
FAM19A1 cg20838162	0.004541424	0.935363199
FAM19A1 cg21186449	0.0404089	0.337855164
FAM19A1 cg13715590	-0.017795022	0.764249258
FAM19A1 cg10131312	-0.084429031	1.10653E-05
FAM19A2 cg10081664	0.024792822	0.667985799
FAM19A2 cg12024564	-0.007183657	0.418410393
FAM19A2 cg16818505	-0.064832803	0.143658825
FAM19A2 cg26133399	0.003593263	0.924447116
FAM19A2 cg02042710	0.000742254	0.873827344
FAM19A2 cg23257289	-0.003619508	0.608037634
FAM19A2 cg25313447	-0.038853579	0.27642167
FAM19A2 cg11885965	-0.02347425	0.620416898
FAM19A2 cg01409597	-0.057821929	0.107574812
FAM19A2 cg09741713	-0.004542129	0.910708105
FAM19A2 cg20010506	-0.085635474	0.000528904
FAM19A2 cg15319470	-0.01899178	0.656819806
FAM19A2 cg12243597	-0.02708309	0.373772978
FAM19A2 cg07000713	-0.001410095	0.883612122
FAM19A2 cg23992583	-0.038419648	0.421833799
FAM19A2 cg24480012	0.006618674	0.905205224
FAM19A2 cg20041366	0.020566265	0.751121119
FAM19A2 cg03001945	-0.0239173	0.234768765
FAM19A2 cg00089798	-0.003597664	0.430412332
FAM19A2 cg15736338	-0.001893449	0.572333962
FAM19A2 cg20007569	-0.058937896	0.165396466
FAM19A2 cg23163783	-0.02255724	0.713222187

FAM19A2 cg23767318	-0.06177816	0.114654256
FAM19A2 cg06438300	0.028444115	0.566617231
FAM19A2 cg22163130	-0.015331175	0.817513817
FAM19A2 cg05145776	-0.002476782	0.972012445
FAM19A3 cg10806711	0.018850861	0.54995709
FAM19A3 cg09126636	0.008513095	0.18242398
FAM19A3 cg16255156	-0.007704655	0.578695104
FAM19A3 cg19675955	-0.010832493	0.833028129
FAM19A3 cg00342891	-0.014109744	0.819020138
FAM19A3 cg07023459	0.007378497	0.351354958
FAM19A3 cg26190476	0.004966679	0.851254816
FAM19A3 cg15070208	-0.003210341	0.954940594
FAM19A3 cg16716189	-0.068471625	8.33387E-05
FAM19A3 cg23746359	0.010867945	0.450566075
FAM19A3 cg02922094	-0.037750754	0.01672228
FAM19A3 cg10834677	-0.059055724	0.003350291
FAM19A4 cg22996308	-0.011557739	0.73107613
FAM19A4 cg15734409	0.021121208	0.601695362
FAM19A4 cg19508285	0.061242733	0.173269322
FAM19A4 cg14581287	0.084904144	0.023462972
FAM19A4 cg22871175	0.01782849	0.755435126
FAM19A4 cg16725723	0.034398035	0.543578222
FAM19A4 cg08855630	0.058172474	0.056339209
FAM19A4 cg03186486	0.079568062	0.021562509
FAM19A4 cg05893614	0.071495376	0.037865272
FAM19A4 cg12417685	0.060737357	0.092068291
FAM19A4 cg03427298	0.018859312	0.733347596
FAM19A4 cg15356923	-0.012471753	0.770205251
FAM19A4 cg13921352	-0.004198216	0.936326076
FAM19A4 cg09868336	-0.055097394	0.04926024
FAM19A4 cg12219082	-0.012104559	0.141800704
FAM19A4 cg20375342	-0.011128608	0.804968353
FAM19A4 cg08038353	0.026203715	0.568118019
FAM19A4 cg12412079	-0.077721452	0.115102117
FAM19A4 cg23967169	-0.061796245	0.046680655
FAM19A4 cg08495813	0.031110742	0.486732374
FAM19A5 cg04377908	-0.03672815	0.101118882
FAM19A5 cg00743803	0.007760672	0.817884774
FAM19A5 cg22504180	0.01014879	0.256383618
FAM19A5 cg21564495	0.014482816	0.227485506
FAM19A5 cg23400715	-0.015566438	0.00705877
FAM19A5 cg23332586	-0.01976863	0.027363473
FAM19A5 cg19122206	-0.035099844	0.058659335
FAM19A5 cg07900958	0.031606579	0.049038923
FAM19A5 cg16609342	0.037975493	0.000568941
FAM19A5 cg14026113	0.075160295	0.015703399
FAM19A5 cg23902471	0.007142039	0.429517309
FAM19A5 cg03440540	0.007728629	0.781285986
FAM19A5 cg20014942	-0.019891968	0.546875347

FAM19A5 cg02582669	-0.009376012	0.822572982
FAM19A5 cg02905830	0.004101041	0.403989442
FAM19A5 cg14894848	0.029608102	0.125254046
FAM19A5 cg11562401	-0.055298952	0.016996579
FAM19A5 cg04304705	-0.023390139	0.229829083
FAM19A5 cg00444884	-0.010536219	0.162497882
FAM19A5 cg02895639	0.008632327	0.828323837
FAM19A5 cg06340899	-0.015201085	0.712459005
FAM19A5 cg25338000	-0.084669727	3.15268E-06
FAM19A5 cg04970014	0.045347535	0.002763036
FAM19A5 cg15665276	0.004369224	0.622102399
FAM19A5 cg10902101	0.016370168	0.19458977
FAM19A5 cg00223715	0.029872415	0.026737718
FAM19A5 cg15187790	0.01387723	0.630933338
FAM19A5 cg19030607	0.040583713	0.114697006
FAM19A5 cg11168293	0.035888417	0.1216771
FAM19A5 cg00286513	0.011851569	0.438831839
FAM19A5 cg22682460	0.016589805	0.119688291
FAM19A5 cg25975712	0.015390889	0.127726696
FAM19A5 cg07416315	-0.020070324	0.058002082
FAM19A5 cg23936463	0.002543175	0.94469913
FAM19A5 cg19734830	-0.001505562	0.949174418
FAM19A5 cg14263779	0.009202495	0.595070106
FAM19A5 cg22643811	8.90624E-05	0.995419705
FAM19A5 cg18013185	-0.017519972	0.654981999
FAM19A5 cg04012844	-0.001514042	0.956295915
FAM19A5 cg06937832	0.020484684	0.466367482
FAM19A5 cg15977904	-0.0032563	0.844508518
FAM19A5 cg15271942	0.071421252	5.56685E-05
FAM19A5 cg12539917	-0.006396275	0.374384473
FAM19A5 cg04382374	-0.000217826	0.977259551
FAM19A5 cg05072413	0.004652969	0.77514505
FAM19A5 cg26700320	0.005849537	0.416231839
FAM19A5 cg24343361	-0.022342323	0.336685597
FAM19A5 cg22989103	0.083086792	0.0026404
FAM19A5 cg12986453	-0.002452063	0.437100012
FAM19A5 cg27652464	0.072015478	0.002147594
FAM19A5 cg04302775	-0.071478425	4.93953E-06
FAM19A5 cg13868165	0.027212074	0.016281929
FAM19A5 cg14984160	0.026429249	0.036212233
FAM19A5 cg00481629	0.065580125	0.007501935
FAM19A5 cg13872278	-0.005747837	0.898556942
FAM19A5 cg05532594	0.004412277	0.816217242
FAM19A5 cg00147509	-0.058527805	0.008337943
FAM19A5 cg16909646	-0.005051874	0.721412748
FAM19A5 cg11352418	0.006269859	0.890701952
FAM19A5 cg08719296	0.008289927	0.578281703
FAM19A5 cg24222175	0.008807696	0.44430434
FAM19A5 cg20640347	-0.091702025	4.5308E-05

FAM19A5 cg20503649	0.000634086	0.96450253
FAM19A5 cg01816146	0.011688819	0.633275693
FAM19A5 cg07305859	-0.023755671	0.001425301
FAM19A5 cg01730859	-0.104334788	0.000507047
FAM19A5 cg05155289	0.014040637	0.20515973
FAM19A5 cg14596108	0.018629397	0.351633262
FAM19A5 cg06273376	-0.070352502	0.046917303
FAM19A5 cg12454595	0.011398338	0.736012824
FAM19A5 cg01616797	0.021187222	0.3802784
FAM19A5 cg19203800	-0.068538008	0.013375907
FAM19A5 cg26120340	-0.052457901	0.000449678
FAM19A5 cg11403706	-0.01869913	0.042111709
FAM19A5 cg03312805	0.000931697	0.974522172
FAM19A5 cg05992904	-0.025488632	0.047273538
FAM19A5 cg08760753	-0.037028436	0.00304175
FAM19A5 cg04198308	-0.005659041	0.897435986
FAM19A5 cg16243558	-0.005762567	0.352400949
FAM19A5 cg27308329	-0.037440112	0.029870686
FAM19A5 cg14517004	-0.014588643	0.016164099
FAM19A5 cg04478606	-0.011808816	0.426708631
FAM19A5 cg04930211	0.006865498	0.565301419
FAM19A5 cg03196739	0.045716659	0.210108683
FAM19A5 cg04493000	0.006774463	0.809089957
FAM19A5 cg27309229	-0.014737795	0.502714415
FAM19A5 cg05006211	0.023036673	0.025935051
FAM19A5 cg23678800	0.038606081	0.010971185
FAM19A5 cg24094706	0.000891297	0.916661555
FAM19A5 cg01216108	0.001047254	0.916118886
FAM19A5 cg00373397	-0.009219385	0.070145625
GRIK1 cg01622416	-0.069971392	4.34234E-07
GRIK1 cg07028661	-0.034300068	0.026839287
GRIK1 cg07950000	-0.012509117	6.52709E-05
GRIK1 cg08685096	-0.00429429	0.381047648
GRIK1 cg09199698	-0.007273153	0.029852865
GRIK1 cg09542111	-0.004932496	0.044463315
GRIK1 cg12079381	-0.09193351	0.001208047
GRIK1 cg21816539	-0.004396307	0.113207434
GRIK1 cg23475371	-0.010276781	0.079981021
GRIK1 cg24620508	-0.072669287	5.12133E-08
GRIK1 cg24868359	-0.003155958	0.745987057
**GRIK2 cg00135293**	-0.020128334	0.600792147
**GRIK2 cg01737026**	-0.001760317	0.960435049
**GRIK2 cg02011392**	-0.0387199	0.146367155
**GRIK2 cg02063520**	-0.002745582	0.971634631
**GRIK2 cg02837591**	-0.000621842	0.959434034
**GRIK2 cg04498418**	-0.022704374	0.061279581
**GRIK2 cg04708601**	0.055951405	0.087930215
**GRIK2 cg05666036**	-0.068849617	0.021866531

**GRIK2 cg05942459**	-0.011276201	0.750989327
**GRIK2 cg06247406**	-0.019087852	0.508928305
**GRIK2 cg09447435**	-0.005892194	0.594360451
**GRIK2 cg10583180**	0.026806958	0.452580288
**GRIK2 cg10591607**	-0.019016433	0.34628064
**GRIK2 cg10815152**	-0.053058349	0.026368963
**GRIK2 cg11976671**	0.008661838	0.756955147
**GRIK2 cg12245867**	0.003699321	0.591113455
**GRIK2 cg13080565**	-0.020472195	0.517376623
**GRIK2 cg13396607**	-0.092594269	0.014210684
**GRIK2 cg14082127**	-0.01539308	0.5369419
**GRIK2 cg15692593**	-0.047184001	0.027714605
**GRIK2 cg16009558**	-0.012949622	0.543244336
**GRIK2 cg18193094**	-0.017231501	0.456156082
**GRIK2 cg19088553**	0.014959205	0.373609502
**GRIK2 cg19155518**	-0.009099117	0.614478977
**GRIK2 cg20640499**	-0.011081707	0.52253217
**GRIK2 cg21635870**	-0.015150244	0.626410557
**GRIK2 cg22541254**	-0.01659057	0.517583686
**GRIK2 cg22851944**	-0.006674575	0.787421753
**GRIK2 cg23666299**	0.043797901	0.093329036
**GRIK2 cg24301620**	-0.020664797	0.436557075
**GRIK2 cg24432193**	0.034705148	0.077880954
**GRIK2 cg24539500**	0.013544392	0.090413022
**GRIK2 cg24574819**	-0.19115214	4.99826E-12
**GRIK2 cg24680758**	-0.010030595	0.64924192
**GRIK2 cg24753760**	-0.020792356	0.419247348
**GRIK2 cg26316946**	-0.015643415	0.504979631
**GRIK2 cg27074174**	-0.010915639	0.593981056
**GRIK2 cg27451362**	-0.036223756	0.067858421
**GRIK3 cg01577933**	-0.028295056	0.376041199
**GRIK3 cg03654504**	0.045035689	0.047935253
**GRIK3 cg04509024**	-0.003485126	0.868557191
**GRIK3 cg04510788**	-0.021009669	0.220959661
**GRIK3 cg06165395**	-0.030466594	0.457689421
**GRIK3 cg06722633**	-0.014561763	0.279735626
**GRIK3 cg08004425**	-0.004959162	0.740466721
**GRIK3 cg08106961**	0.065157296	0.000156865
**GRIK3 cg09134640**	-0.002115289	0.830768205
**GRIK3 cg09991710**	0.004663927	0.773064542
**GRIK3 cg10126715**	-0.006690835	0.772192402
**GRIK3 cg10206594**	0.006606909	0.779657822
**GRIK3 cg11836212**	0.023854991	0.403812718
**GRIK3 cg13052954**	0.037471325	0.07511663
**GRIK3 cg13272644**	-0.003370402	0.892577068
**GRIK3 cg13934830**	-0.00058238	0.820686982

**GRIK3 cg14616584**	0.01118559	0.620001197
**GRIK3 cg14960282**	0.063135311	1.28279E-05
**GRIK3 cg14992273**	-0.033834865	0.028470113
**GRIK3 cg16379885**	-0.012835402	0.224638829
**GRIK3 cg16599703**	-0.012003416	0.113151105
**GRIK3 cg16911275**	0.019277197	0.444293443
**GRIK3 cg17952046**	0.001486409	0.965816843
**GRIK3 cg19206040**	-0.008774212	0.419493394
**GRIK3 cg19640303**	0.009680366	0.010761839
**GRIK3 cg19727439**	-0.00548066	0.505252739
**GRIK3 cg20168230**	0.001946216	0.946487193
**GRIK3 cg20779373**	-0.027027843	0.004394992
**GRIK3 cg21409965**	0.030606008	0.163486999
**GRIK3 cg23715749**	0.051041237	0.235559942
**GRIK3 cg24673742**	-0.058914665	0.126584351
**GRIK3 cg26337841**	0.027115786	0.04498661
**GRIK3 cg27433516**	-0.105541083	0.003017791
**GRIK4 cg01868106**	0.006294418	0.645264313
**GRIK4 cg03231471**	0.043562058	0.081682232
**GRIK4 cg03303857**	-0.055284057	0.048351378
**GRIK4 cg04925085**	0.001594117	0.608253338
**GRIK4 cg05431964**	-0.000538014	0.967604645
**GRIK4 cg06465194**	0.126405576	6.54441E-10
**GRIK4 cg10961484**	0.133377074	5.09454E-10
**GRIK4 cg11281320**	-0.057869816	0.101346698
**GRIK4 cg14462402**	0.17017561	2.5435E-09
**GRIK4 cg14731698**	0.003652442	0.591767993
**GRIK4 cg15174564**	0.049854548	0.125236214
**GRIK4 cg16361867**	0.023849255	0.618569873
**GRIK4 cg16730369**	-0.001066087	0.919208237
**GRIK4 cg18247436**	0.043775599	0.129132836
**GRIK4 cg23315601**	-0.026643694	0.274647047
**GRIK4 cg23868250**	0.004013482	0.910475514
**GRIK4 cg24226238**	0.043780714	3.37307E-06
**GRIK4 cg24255125**	0.051722682	0.112751961
**GRIK4 cg25113360**	-0.035249231	0.360107472
**GRIK4 cg25316569**	0.105307257	3.03557E-06
**GRIK4 cg26914334**	-0.09017916	1.00579E-05
**GRIK4 cg27179693**	0.17285459	2.70944E-13
**GRIK5 cg00553487**	-0.072494217	2.99598E-10
**GRIK5 cg03100024**	-0.059098359	0.189074239
**GRIK5 cg04695635**	-0.012900578	0.371628066
**GRIK5 cg04763994**	-0.027547444	0.177951069
**GRIK5 cg07746960**	-0.035429657	0.028318884
**GRIK5 cg08955995**	0.072629107	0.017722413
**GRIK5 cg09434500**	0.060131589	0.058025746

**GRIK5 cg09555879**	-0.102587169	0.002428923
**GRIK5 cg09864457**	0.095033994	0.017630048
**GRIK5 cg11640185**	-0.013229889	0.349784787
**GRIK5 cg11744436**	-0.09761625	7.3614E-06
**GRIK5 cg11844965**	0.009910468	0.601701792
**GRIK5 cg11971789**	-0.00660983	0.657449804
**GRIK5 cg12544392**	-0.036816712	0.004817467
**GRIK5 cg12981270**	-0.000758221	0.928082019
**GRIK5 cg13298384**	-0.020855949	0.122115873
**GRIK5 cg13510813**	-0.025072967	0.146821375
**GRIK5 cg14036830**	0.055858367	0.158584428
**GRIK5 cg17540575**	-0.015602268	0.545846583
**GRIK5 cg17726655**	-0.018621789	0.266363699
**GRIK5 cg17742947**	-0.029574445	0.149398432
**GRIK5 cg26314755**	0.00363858	0.899074318
**GRIK5 cg26746936**	0.075461164	0.008887935
**GRIK5 cg27167613**	-0.047701828	0.000979059
**UNC5A cg00041599**	-0.007536122	0.525643417
**UNC5A cg00513735**	-0.015252972	0.350779594
**UNC5A cg01099590**	0.001183798	0.9371469
**UNC5A cg01184387**	0.002895067	0.803126331
**UNC5A cg01476678**	-0.05860822	0.039728066
**UNC5A cg01485938**	0.057688471	0.021416652
**UNC5A cg02114954**	0.010369415	0.798909921
**UNC5A cg02616808**	0.003250855	0.791844849
**UNC5A cg02633413**	-0.034052638	0.006167078
**UNC5A cg03320873**	-0.053426217	0.001762796
**UNC5A cg03679715**	-0.001776915	0.929153254
**UNC5A cg05475205**	-0.004290346	0.915787742
**UNC5A cg05593510**	0.023442194	0.38144765
**UNC5A cg06527989**	-0.007200223	0.418213664
**UNC5A cg06849077**	-0.003798127	0.556980556
**UNC5A cg08029909**	-0.008764444	0.06664846
**UNC5A cg08322194**	0.016015429	0.538222542
**UNC5A cg08494545**	-0.003673939	0.291967392
**UNC5A cg09582314**	0.00751391	0.684918522
**UNC5A cg09901106**	-0.001297936	0.883612122
**UNC5A cg11130778**	0.001601113	0.725560558
**UNC5A cg11139090**	0.017385161	0.395735221
**UNC5A cg11491504**	-0.003025803	0.708024455
**UNC5A cg11918743**	0.038175624	0.151838617
**UNC5A cg12888080**	0.006620476	0.777311483
**UNC5A cg12893143**	-0.005169271	0.255665017
**UNC5A cg13665261**	-0.007499754	0.89378305
**UNC5A cg13928709**	-0.006575117	0.227201654
**UNC5A cg14871923**	-0.000843298	0.769706841

**UNC5A cg14968378**	0.000274428	0.980279233
**UNC5A cg15366301**	0.118745819	0.000180941
**UNC5A cg16268165**	0.035876471	0.396356107
**UNC5A cg16413715**	0.019931258	0.439712744
**UNC5A cg16457351**	-0.031138426	0.387796215
**UNC5A cg16635016**	-0.004594155	0.878710855
**UNC5A cg17449197**	0.118299398	1.1167E-06
**UNC5A cg18422123**	-0.001806088	0.698420767
**UNC5A cg18585314**	0.066164052	0.001485237
**UNC5A cg19091621**	-0.002710309	0.903540117
**UNC5A cg19742888**	-0.01719562	0.402746175
**UNC5A cg22338352**	-0.005455965	0.344869905
**UNC5A cg22957659**	-0.046818269	0.048985094
**UNC5A cg23292965**	-0.062582336	0.056490421
**UNC5A cg23929295**	-0.000249792	0.963379078
**UNC5A cg24575624**	0.048237497	0.059241835
**UNC5A cg24600724**	-0.070617223	0.002190741
**UNC5A cg24886356**	-0.051831504	0.182061774
**UNC5A cg25389791**	0.012330235	0.204107259
**UNC5A cg25429640**	0.108889214	0.000158696
**UNC5A cg25439951**	0.002514633	0.885188887
**UNC5A cg25667000**	-0.041090054	0.002362208
**UNC5B cg02378006**	-0.109907632	1.49115E-07
**UNC5B cg02440719**	0.020547069	0.015148925
**UNC5B cg04177244**	-0.049610299	1.35799E-09
**UNC5B cg04269057**	-0.050789002	0.095304388
**UNC5B cg05871136**	-0.004896491	0.483893959
**UNC5B cg08342721**	-0.001896888	0.800492486
**UNC5B cg08905239**	-0.043097199	3.67194E-05
**UNC5B cg09470230**	9.85344E-05	0.98962919
**UNC5B cg12873350**	-0.005732747	0.834309265
**UNC5B cg14042687**	-0.004326534	0.179807661
**UNC5B cg14957186**	0.027661099	0.25753325
**UNC5B cg15929693**	0.000794879	0.946583268
**UNC5B cg16759416**	-0.072636818	0.002077492
**UNC5B cg17477835**	-0.005041783	0.826960813
**UNC5B cg17668756**	-0.133287954	4.3632E-12
**UNC5B cg18317005**	-0.099897895	1.05684E-12
**UNC5B cg18389931**	0.009062819	0.635990948
**UNC5B cg18858343**	-0.001217485	0.791214734
**UNC5B cg19673155**	0.033938859	0.098661842
**UNC5B cg20254483**	-0.004316144	0.198427277
**UNC5B cg23622162**	0.08671878	7.62201E-05
**UNC5B cg23967873**	-0.002644854	0.786330725
**UNC5B cg24591279**	-0.033685518	0.00634024
**UNC5B cg26152017**	0.02891285	0.443944126

**UNC5B cg26450586**	-0.001105355	0.878394705
**UNC5C cg00329039**	-0.001125642	0.771942639
**UNC5C cg01194057**	0.000404899	0.948104346
**UNC5C cg01462668**	-0.081218319	1.12971E-08
**UNC5C cg01788994**	1.15654E-05	0.999539164
**UNC5C cg02931225**	-0.078360859	0.018850169
**UNC5C cg03008707**	-0.027465931	0.02266337
**UNC5C cg03024478**	-0.020060519	0.383952375
**UNC5C cg04281738**	-0.068099759	0.005425496
**UNC5C cg05121379**	-0.041678441	0.004622536
**UNC5C cg07234102**	-0.001526201	0.715325507
**UNC5C cg07824265**	-0.012242899	0.099359565
**UNC5C cg09100013**	-0.000566775	0.93889565
**UNC5C cg10520887**	0.001326674	0.862274314
**UNC5C cg10528218**	-0.083740192	2.36848E-07
**UNC5C cg11723848**	-0.000252461	0.977130515
**UNC5C cg12412390**	-0.005593698	0.144289544
**UNC5C cg12553181**	-0.01020426	0.619569029
**UNC5C cg12584684**	-0.001490055	0.712797901
**UNC5C cg12802900**	-0.000797007	0.92118704
**UNC5C cg13265789**	-0.000981681	0.830021307
**UNC5C cg14101976**	-0.027547697	0.524195491
**UNC5C cg15701178**	0.002492509	0.42633505
**UNC5C cg15984718**	-0.004533261	0.53794356
**UNC5C cg17031751**	-0.007608073	0.158829332
**UNC5C cg17334018**	-0.001577248	0.771691846
**UNC5C cg17337917**	0.015883229	0.783776494
**UNC5C cg17504765**	-0.010636746	0.54081106
**UNC5C cg21574186**	-0.026784886	0.423342141
**UNC5C cg21782409**	-0.002308527	0.48125643
**UNC5C cg22490991**	-0.013148121	0.842508099
**UNC5C cg22634891**	0.00284054	0.368008721
**UNC5C cg23542495**	-0.004304496	0.54300481
**UNC5C cg24870497**	-0.001008719	0.925113747
**UNC5CL cg00592058**	0.003643109	0.811208391
**UNC5CL cg03068376**	0.010820337	0.287726956
**UNC5CL cg04650094**	0.025321696	0.329774972
**UNC5CL cg05673137**	-0.049667103	0.065108749
**UNC5CL cg09489757**	-0.021543866	0.303778792
**UNC5CL cg09712135**	0.019520537	0.273720252
**UNC5CL cg14418226**	0.01325785	0.665093321
**UNC5CL cg14769373**	-0.035728483	0.184846895
**UNC5CL cg15849648**	-0.013439662	0.400543312
**UNC5CL cg17826424**	-0.006780925	0.641011446
**UNC5CL cg19175193**	-0.010099019	0.679811644
**UNC5CL cg21128951**	0.038008454	0.136118974

**UNC5CL cg21961970**	-0.082513765	0.02153247
**UNC5CL cg22346765**	0.023240291	0.45676344
**UNC5CL cg24419528**	-0.000811351	0.966984271
**UNC5D cg00297600**	-0.03300368	0.498870162
**UNC5D cg00948124**	-0.067766969	0.07199363
**UNC5D cg01311313**	-0.06803204	0.09452835
**UNC5D cg02355183**	-0.027532914	0.40327991
**UNC5D cg03752965**	-0.039212309	0.330552702
**UNC5D cg03995061**	-0.020908547	0.051864433
**UNC5D cg04402007**	-0.019587539	0.143817081
**UNC5D cg07396194**	-0.067745809	0.034731609
**UNC5D cg07574158**	-0.069516783	0.01899353
**UNC5D cg08000065**	-0.014607759	0.42139937
**UNC5D cg08300924**	-0.008641555	0.821839049
**UNC5D cg08701686**	-0.143124622	9.57268E-08
**UNC5D cg08754088**	-0.022278703	0.716276551
**UNC5D cg10393548**	-0.018285712	0.693723323
**UNC5D cg10861722**	-0.040031648	0.095223035
**UNC5D cg11093149**	-0.011790371	0.587383299
**UNC5D cg13561879**	-0.055458197	0.188073354
**UNC5D cg15981092**	-0.011181274	0.14411237
**UNC5D cg16137995**	-0.002502902	0.710022195
**UNC5D cg17486097**	-0.00797117	0.528467805
**UNC5D cg18583697**	-0.047631749	0.063739484
**UNC5D cg18760194**	-0.040588579	0.059516728
**UNC5D cg19003975**	-0.045754689	0.224765601
**UNC5D cg19403244**	-0.028013455	0.135718799
**UNC5D cg20776920**	-0.000874834	0.877421421
**UNC5D cg21320573**	-0.076326652	0.034206934
**UNC5D cg22386073**	-0.060308427	0.022212243
**UNC5D cg22507558**	-0.059527961	0.133839647
**UNC5D cg22872195**	-0.116449613	0.009019717
**UNC5D cg23222401**	0.043924024	0.099087615
**UNC5D cg23279503**	-0.015410634	0.792450838
**UNC5D cg26679047**	-0.021692853	0.690054891
**UNC5D cg26764980**	0.045489672	0.201139294
**UNC5D cg26872137**	-0.006536264	0.789242461
**UNC5D cg27244980**	-0.159618899	5.02356E-05
**DCC cg00027400**	-0.023112383	0.004957508
**DCC cg00399483**	-0.049214229	0.022506377
**DCC cg01839464**	-0.014686498	0.531123094
**DCC cg02835371**	-0.095933164	0.000334221
**DCC cg02837027**	-0.045527573	0.21054508
**DCC cg03458463**	-0.16038942	2.95766E-06
**DCC cg04272086**	-0.037289137	0.014261297
**DCC cg05420896**	-0.035272823	0.004859807

**DCC cg06261937**	-0.026723904	0.055989861
**DCC cg06784848**	-0.082724601	0.005633997
**DCC cg07897248**	-0.020109275	0.286278269
**DCC cg07935012**	-0.017185342	0.077786532
**DCC cg14222939**	-0.050828134	0.000801518
**DCC cg14268020**	-0.110281923	0.000351
**DCC cg14743291**	-0.008897415	0.425332329
**DCC cg16080643**	-0.148388568	3.94233E-08
**DCC cg16964748**	-0.086824028	0.0024234
**DCC cg18801691**	-0.013260412	0.438162751
**DCC cg18841634**	-0.023872109	0.111293966
**DCC cg18866015**	-0.014046481	0.575186323
**DCC cg18874136**	-0.008904603	0.492541362
**DCC cg19042459**	-0.017835553	0.26956655
**DCC cg19613722**	-0.016991497	0.300232864
**DCC cg19995964**	0.008557213	0.332125651
**DCC cg20917891**	-0.028097399	0.053274621
**DCC cg21263710**	-0.048149354	0.21498732
**DCC cg21381065**	-0.040409808	0.026618793
**DCC cg21623566**	-0.015800086	0.448620972
**DCC cg21669679**	-0.013319599	0.421483451
**DCC cg23242898**	-0.007489266	0.586584806
**DCC cg24084891**	-0.006857663	0.815671857
**DCC cg24270629**	-0.135326822	2.71242E-08
**DCC cg25122395**	-0.038967422	0.045588904
**DCC cg25204852**	-0.054082877	0.221567329
**DCC cg25266232**	-0.019485661	0.027045667
**DCC cg25602457**	-0.022506637	0.179908701
**DCC cg25818763**	0.02481603	0.560395166
**NEUROG1 cg00513205**	0.097645824	1.37678E-11
**NEUROG1 cg02604503**	0.068383477	0.003694839
**NEUROG1 cg04063589**	-0.002412863	0.960643182
**NEUROG1 cg04469059**	-0.01550726	0.816428997
**NEUROG1 cg04620091**	-0.004328456	0.880317158
**NEUROG1 cg04897683**	0.03321446	0.456995626
**NEUROG1 cg07035503**	-0.011263121	0.857567531
**NEUROG1 cg08285915**	-7.29521E-05	0.999172791
**NEUROG1 cg08451582**	0.078158912	0.003221698
**NEUROG1 cg08587845**	-0.045539999	0.204974297
**NEUROG1 cg11144641**	0.016607387	0.615375184
**NEUROG1 cg11248413**	-0.013787307	0.628582872
**NEUROG1 cg11260904**	0.026952911	0.543497425
**NEUROG1 cg11946503**	-0.045255487	0.275496724
**NEUROG1 cg12065579**	-0.014338696	0.756311278
**NEUROG1 cg13336665**	0.022661764	0.385566583
**NEUROG1 cg14747563**	-0.010438719	0.862373419

**NEUROG1 cg14958635**	0.081695138	0.009653663
**NEUROG1 cg17772342**	0.003478003	0.891165147
**NEUROG1 cg17830308**	-0.031539316	0.606582137
**NEUROG1 cg18362330**	0.067134056	2.47814E-05
**NEUROG1 cg19951663**	-0.010072803	0.657516221
**NEUROG1 cg22630755**	-0.002130736	0.958486231
**NEUROG1 cg22734397**	-0.010083689	0.845355546
**NEUROG1 cg24899138**	0.063481951	3.47062E-07
**NEUROG1 cg25640822**	-0.001332108	0.965538853
**NEUROG2 cg02182354**	0.000198004	0.991730784
**NEUROG2 cg06367702**	-0.016954551	0.717315882
**NEUROG2 cg07131451**	-0.000159515	0.994387906
**NEUROG2 cg07964553**	0.047329264	0.239905446
**NEUROG2 cg07997294**	-0.005316058	0.484558808
**NEUROG2 cg08929548**	-0.000673976	0.989973016
**NEUROG2 cg09707262**	0.002489214	0.937592044
**NEUROG2 cg10511618**	0.026677602	0.498584205
**NEUROG2 cg10871779**	-0.005891235	0.374760964
**NEUROG2 cg15463803**	-0.01163713	0.801092305
**NEUROG2 cg17031795**	-0.001275158	0.982957274
**NEUROG2 cg17267256**	-0.005472446	0.628289573
**NEUROG2 cg26708817**	0.000586618	0.931708061
**NEUROG3 cg02262662**	-0.011031281	0.589709787
**NEUROG3 cg03425110**	-0.037949466	0.197630672
**NEUROG3 cg05886671**	0.004568658	0.901081195
**NEUROG3 cg06043042**	-0.010562585	0.617475263
**NEUROG3 cg06973667**	-0.025473089	0.543671744
**NEUROG3 cg07647353**	-0.004776538	0.876569433
**NEUROG3 cg09154558**	0.004067249	0.856972142
**NEUROG3 cg10001282**	-0.00749964	0.78518205
**NEUROG3 cg12164232**	-0.003660572	0.913071989
**NEUROG3 cg12938159**	-0.026920295	0.535567377
**NEUROG3 cg17583413**	0.006508174	0.892053155
**NEUROG3 cg18834338**	-0.008798746	0.803201128
**NEUROG3 cg26333837**	0.031531598	0.276892842
**NLGN1 cg00493429**	0.028199767	0.000648457
**NLGN1 cg01946530**	-0.002317371	0.76262634
**NLGN1 cg02521771**	-0.047956239	0.062819411
**NLGN1 cg03729941**	-0.017330491	0.418789876
**NLGN1 cg04812049**	0.000749608	0.88756375
**NLGN1 cg05058204**	-0.012657931	0.478410836
**NLGN1 cg05543049**	-0.004524598	0.291825395
**NLGN1 cg05722552**	-0.003373462	0.400931434
**NLGN1 cg06097077**	-0.109670854	7.85213E-12
**NLGN1 cg06304401**	-0.002215037	0.6226084
**NLGN1 cg07452098**	0.035906666	0.010811903

**NLGN1 cg07489890**	-0.002774081	0.289027178
**NLGN1 cg09229620**	-0.033134904	1.34937E-06
**NLGN1 cg10623221**	-0.040669824	0.214309068
**NLGN1 cg11733675**	-0.000103911	0.995214913
**NLGN1 cg13238990**	-0.003548308	0.458243068
**NLGN1 cg13582793**	-0.005875035	0.117222546
**NLGN1 cg14559455**	-0.014206697	0.711881413
**NLGN1 cg14890220**	-0.000454954	0.995072544
**NLGN1 cg15225201**	-0.004823628	0.052097789
**NLGN1 cg15558616**	-0.090793979	0.002003397
**NLGN1 cg17482360**	0.016617082	0.343276524
**NLGN1 cg19245371**	-0.005532647	0.076338805
**NLGN1 cg19510409**	0.006175172	0.597344833
**NLGN1 cg21120436**	-0.0288929	9.8689E-06
**NLGN1 cg21459053**	-0.005200433	0.09995229
**NLGN1 cg21548131**	-0.136087384	0.000198604
**NLGN1 cg21904441**	0.010367183	0.099474302
**NLGN1 cg21946824**	-0.038312723	0.001795852
**NLGN1 cg22960621**	0.01214614	0.827544035
**NLGN1 cg23642200**	-0.0012599	0.659628561
**NLGN1 cg24600895**	0.000758806	0.874293562
**NLGN1 cg27315556**	-0.005551015	0.115907553
**NLGN2 cg00061695**	0.065819737	4.62884E-07
**NLGN2 cg03169180**	-0.15445738	5.7851E-08
**NLGN2 cg03403688**	-0.004669563	0.627782831
**NLGN2 cg03461559**	-0.007437015	0.623400062
**NLGN2 cg04235625**	-0.077758765	0.003594283
**NLGN2 cg07379741**	-0.063420449	2.41268E-05
**NLGN2 cg08870143**	-0.03108294	0.069007255
**NLGN2 cg09568217**	0.055555329	0.000110235
**NLGN2 cg09711113**	0.02019706	0.023295041
**NLGN2 cg10092265**	0.084670984	3.42954E-05
**NLGN2 cg10369169**	-0.005341003	0.668911387
**NLGN2 cg13177052**	-0.037397563	0.259720532
**NLGN2 cg15660077**	-0.008455357	0.872993048
**NLGN2 cg16950726**	-0.037510932	0.159712753
**NLGN2 cg18089810**	-0.019986742	0.257699133
**NLGN2 cg20718319**	0.046004441	0.015990396
**NLGN2 cg22659681**	-0.009378625	0.481462638
**NLGN2 cg25617307**	-0.019834324	0.000317645
**NRXN1 cg00345522**	-0.076998922	0.013244587
**NRXN1 cg01014190**	-0.231116393	1.3528E-08
**NRXN1 cg02495518**	-0.018700617	0.743425141
**NRXN1 cg03168108**	-0.005285834	0.400187033
**NRXN1 cg03295083**	0.052136224	0.13377821
**NRXN1 cg03531247**	0.114461242	1.08749E-08

**NRXN1 cg03714495**	-0.010665866	0.306863714
**NRXN1 cg04595505**	0.088429969	0.000252312
**NRXN1 cg04707706**	-0.005751747	0.916514781
**NRXN1 cg05015185**	0.051776917	0.082150112
**NRXN1 cg05495351**	-0.013224797	0.032645542
**NRXN1 cg06466689**	0.0034697	0.445063799
**NRXN1 cg06609051**	0.007443191	0.83127653
**NRXN1 cg06707406**	-0.186775888	5.30485E-08
**NRXN1 cg06749295**	-0.120802444	2.2904E-05
**NRXN1 cg07083023**	0.006810453	0.346776443
**NRXN1 cg07529754**	0.009803051	0.849194149
**NRXN1 cg07781364**	-0.032452367	0.2442507
**NRXN1 cg08008931**	-0.001694793	0.747781104
**NRXN1 cg08189280**	0.01903249	0.019304181
**NRXN1 cg08425543**	0.045352292	8.56418E-07
**NRXN1 cg09044294**	0.02644071	0.090279962
**NRXN1 cg09271709**	0.014700264	0.78877299
**NRXN1 cg09351848**	0.021318291	0.280760317
**NRXN1 cg10099415**	0.052381899	0.126038486
**NRXN1 cg10756457**	-0.189956354	6.60031E-12
**NRXN1 cg10917619**	-0.06807527	0.026704023
**NRXN1 cg10919889**	0.026688258	0.237915904
**NRXN1 cg11430766**	0.000691061	0.958767674
**NRXN1 cg11775579**	0.002740271	0.913763396
**NRXN1 cg12001457**	0.018292903	0.550835363
**NRXN1 cg12167526**	-0.035307373	0.335376767
**NRXN1 cg12446209**	-0.192843709	7.17106E-09
**NRXN1 cg12884814**	-0.029067593	0.127973937
**NRXN1 cg13204432**	0.001564141	0.958516768
**NRXN1 cg13594075**	-0.05090141	0.005298569
**NRXN1 cg13906811**	-0.013201999	0.796323445
**NRXN1 cg13999106**	-0.025678798	0.002017023
**NRXN1 cg14329860**	-0.086373314	0.000395276
**NRXN1 cg14875171**	0.085439558	0.061509109
**NRXN1 cg15558448**	0.000359183	0.98444765
**NRXN1 cg15573998**	-0.092834174	6.07251E-07
**NRXN1 cg15912010**	-0.043640118	0.037016553
**NRXN1 cg16852792**	-0.007349014	0.617524076
**NRXN1 cg17526573**	0.067353125	0.138419085
**NRXN1 cg18201351**	0.034782193	0.304765082
**NRXN1 cg18396357**	-0.058516547	0.000139474
**NRXN1 cg18773591**	-0.054289066	0.013616628
**NRXN1 cg19824059**	0.005767088	0.51373754
**NRXN1 cg20062057**	-0.050985704	2.19827E-05
**NRXN1 cg20536469**	-0.049696391	6.28673E-05
**NRXN1 cg21203781**	-0.248964524	9.40741E-11

**NRXN1 cg22001533**	0.045520909	0.195294683
**NRXN1 cg22324022**	-0.003246461	0.664276316
**NRXN1 cg22573528**	-0.201568395	9.95704E-10
**NRXN1 cg23273041**	0.008558647	0.161077683
**NRXN1 cg23601521**	0.029153095	0.07041941
**NRXN1 cg23851515**	-0.129642029	3.54583E-10
**NRXN1 cg24402935**	0.001798038	0.740871271
**NRXN1 cg25036261**	0.01063642	0.007021381
**NRXN1 cg25278175**	-0.080070868	2.37835E-05
**NRXN1 cg25565504**	-0.01330631	0.748727148
**NRXN1 cg25717438**	-0.013935843	0.006152317
**NRXN1 cg26881899**	-0.01019502	0.381629854
**NRXN1 cg27112565**	-0.004810706	0.055405415
**NRXN1 cg27456111**	-0.082678555	3.91435E-06
**NRXN2 cg00599252**	-0.003780936	0.24149516
**NRXN2 cg00668337**	-0.012613607	0.738775621
**NRXN2 cg01579950**	0.078487243	3.12933E-05
**NRXN2 cg02961807**	-0.021825965	0.00077307
**NRXN2 cg03035661**	-0.002296883	0.7495305
**NRXN2 cg03263716**	0.014713105	0.149872119
**NRXN2 cg03586390**	-0.080651353	0.012590667
**NRXN2 cg03982537**	-0.034832121	0.099246271
**NRXN2 cg04686778**	-0.000667387	0.967526293
**NRXN2 cg05267623**	-0.000306114	0.933103364
**NRXN2 cg06055392**	-0.098513258	4.51E-12
**NRXN2 cg07256668**	0.00667845	0.450206286
**NRXN2 cg07712329**	-0.002369182	0.62011162
**NRXN2 cg07733098**	-0.056342656	0.022313403
**NRXN2 cg08011645**	-0.116728736	5.6009E-07
**NRXN2 cg08623810**	0.030119495	0.377230608
**NRXN2 cg08800856**	-0.152188629	2.61118E-06
**NRXN2 cg09072148**	-0.167900333	2.94274E-06
**NRXN2 cg09129050**	-0.049439741	0.162225834
**NRXN2 cg09526022**	-0.000528881	0.943970278
**NRXN2 cg09948336**	-0.005312713	0.813553098
**NRXN2 cg10571796**	0.022465402	0.283662607
**NRXN2 cg10885956**	-0.012106678	0.35011087
**NRXN2 cg10905012**	-0.024237276	0.01541842
**NRXN2 cg10940462**	-0.086607071	0.078861217
**NRXN2 cg12129012**	-0.1211074	5.24343E-08
**NRXN2 cg12403546**	0.000359525	0.965899004
**NRXN2 cg12840818**	-0.065163418	5.35056E-09
**NRXN2 cg12938128**	-0.133503096	5.87947E-05
**NRXN2 cg13015284**	-0.042291503	0.000175189
**NRXN2 cg14053234**	0.011289205	0.297099895
**NRXN2 cg15939287**	-0.168426272	6.34474E-06

**NRXN2 cg16527223**	-0.000725041	0.891093339
**NRXN2 cg16718678**	-0.000497366	0.922979234
**NRXN2 cg16958106**	-0.007686339	0.404570963
**NRXN2 cg17059853**	-0.010775813	0.000795746
**NRXN2 cg17219981**	0.000388721	0.946617206
**NRXN2 cg17221226**	-0.134593713	1.52836E-06
**NRXN2 cg17236169**	0.094825808	3.20557E-07
**NRXN2 cg17491228**	-0.067397454	0.012641361
**NRXN2 cg18431183**	-0.008281302	0.77561974
**NRXN2 cg19131476**	-0.089508557	1.15649E-08
**NRXN2 cg19395706**	-0.022035086	0.323466551
**NRXN2 cg19691659**	-0.070757415	0.002342268
**NRXN2 cg20234205**	-0.088334026	0.000345919
**NRXN2 cg20718855**	-0.05118383	0.10349919
**NRXN2 cg21533897**	0.004759923	0.889079549
**NRXN2 cg21660452**	-0.003309268	0.653154036
**NRXN2 cg21751231**	-0.000930962	0.817098067
**NRXN2 cg21996761**	-0.111232923	0.000750119
**NRXN2 cg22159835**	-0.053944126	0.086551167
**NRXN2 cg22187354**	0.1548803	9.05119E-09
**NRXN2 cg22797270**	0.064027832	0.0361492
**NRXN2 cg23182880**	-0.101825739	5.38117E-07
**NRXN2 cg23201340**	-0.00944919	0.65987091
**NRXN2 cg23389215**	-0.067953455	4.97365E-07
**NRXN2 cg23907108**	-0.106053451	8.38198E-06
**NRXN2 cg24007388**	0.013401087	0.684704189
**NRXN2 cg24331824**	0.013209123	0.376704946
**NRXN2 cg24407327**	-0.011072321	0.045752458
**NRXN2 cg24524285**	-0.07378132	0.002083501
**NRXN2 cg25073093**	0.000918814	0.968067387
**NRXN2 cg25104637**	-0.121539894	7.16467E-09
**NRXN2 cg25321556**	-0.047911889	0.024931773
**NRXN2 cg26112489**	-0.028735524	0.193240474
**NRXN2 cg26388730**	-0.111355864	8.31997E-05
**NRXN2 cg26661640**	-0.055145067	0.021698564
**NRXN2 cg26732720**	0.005880075	0.894867617
**NRXN2 cg26805405**	-0.003832411	0.442550119
**NRXN2 cg27017482**	0.076878602	0.003575007
**NRXN2 cg27122888**	0.109978875	3.96198E-11
**NRXN2 cg27209729**	-0.084601708	1.61015E-07
**NRXN2 cg27243166**	0.010469906	0.655461773
**NRXN2 cg27319263**	-0.062116274	0.001988697
**NRXN2 cg27466845**	-0.014732173	0.611117423
**NRXN3 cg00685573**	-0.029842043	0.400040983
**NRXN3 cg02030212**	-0.048186476	0.017016969
**NRXN3 cg02111786**	0.000164515	0.988558802

**NRXN3 cg02326285**	-0.007503481	0.662909883
**NRXN3 cg02971654**	-0.005928766	0.652235219
**NRXN3 cg04272011**	0.032801793	0.194411866
**NRXN3 cg04324727**	-0.003181602	0.665193736
**NRXN3 cg04549090**	-0.002082381	0.655854521
**NRXN3 cg04897790**	-0.017779393	0.645194559
**NRXN3 cg05175318**	0.081314501	0.001164367
**NRXN3 cg05280527**	0.011834144	0.784078938
**NRXN3 cg05850716**	0.013319074	0.362652957
**NRXN3 cg07001909**	0.00034379	0.963063998
**NRXN3 cg07147443**	0.000112119	0.995885653
**NRXN3 cg07177395**	-0.00551162	0.912972478
**NRXN3 cg07182163**	0.094221533	0.006999947
**NRXN3 cg07231787**	-0.001419845	0.904676641
**NRXN3 cg08186935**	-0.063232331	0.006088399
**NRXN3 cg09209257**	0.049398482	0.10286734
**NRXN3 cg09340276**	-0.004493541	0.949908998
**NRXN3 cg09950309**	-0.005687979	0.806011028
**NRXN3 cg09971881**	-0.019682707	0.766562908
**NRXN3 cg10015904**	-0.057622408	0.16599625
**NRXN3 cg10186254**	-0.10747234	0.000943635
**NRXN3 cg10737847**	-0.000362923	0.990285835
**NRXN3 cg10991579**	0.048524668	0.17413331
**NRXN3 cg12101264**	-0.051380972	0.091039522
**NRXN3 cg12779438**	0.000551039	0.97761696
**NRXN3 cg13101330**	0.034822903	0.309337613
**NRXN3 cg13564020**	-0.093644295	0.009745058
**NRXN3 cg13680118**	-0.018683523	0.287928743
**NRXN3 cg13833923**	0.060501715	0.049289724
**NRXN3 cg14317273**	-0.007013796	0.814056293
**NRXN3 cg14335579**	0.003075522	0.668812061
**NRXN3 cg14527639**	-0.021677299	0.659639871
**NRXN3 cg15320277**	0.004095732	0.953474678
**NRXN3 cg15434778**	-0.070546016	0.029157432
**NRXN3 cg15572745**	0.049739023	0.303496397
**NRXN3 cg16372520**	0.080758822	0.001957202
**NRXN3 cg16420308**	-0.066731249	0.013596777
**NRXN3 cg16766249**	-0.001195241	0.954539854
**NRXN3 cg18199664**	0.027293874	0.327325237
**NRXN3 cg18311665**	-0.041405863	0.374571339
**NRXN3 cg18422058**	0.026097407	0.575175343
**NRXN3 cg18459339**	-0.008147111	0.893488819
**NRXN3 cg18597220**	0.04182567	0.014392394
**NRXN3 cg18818949**	-0.016816823	0.78781748
**NRXN3 cg19662611**	-0.015813812	0.676224066
**NRXN3 cg19753609**	-0.098768067	0.004017397

**NRXN3 cg20076659**	-0.054283587	3.02016E-07
**NRXN3 cg20312971**	0.087981039	0.007767085
**NRXN3 cg21010031**	0.002493962	0.929030583
**NRXN3 cg21061859**	-0.059744016	0.056590564
**NRXN3 cg21113554**	0.0531426	0.007229184
**NRXN3 cg21918126**	0.001093819	0.883853257
**NRXN3 cg22828707**	-0.008770295	0.896016468
**NRXN3 cg22878414**	0.028871583	0.133542628
**NRXN3 cg22892860**	0.009380972	0.525966059
**NRXN3 cg22908679**	0.015533593	0.35839617
**NRXN3 cg23949782**	0.052686111	0.124242879
**NRXN3 cg24020215**	0.079809449	0.04488768
**NRXN3 cg24197470**	-0.003549642	0.693975129
**NRXN3 cg25564511**	-0.024672647	0.067314097
**EFN2B cg00241273**	-0.084633733	0.011724131
**EFN2B cg02441149**	-0.001682171	0.928691925
**EFN2B cg03929741**	-0.000242116	0.981081656
**EFN2B cg04321866**	0.00797203	0.282306342
**EFN2B cg05040544**	0.000656304	0.915637252
**EFN2B cg05493945**	0.01033766	0.471400783
**EFN2B cg05876564**	-0.078543536	0.064472108
**EFN2B cg06193439**	-0.001841815	0.74800332
**EFN2B cg06784108**	-0.004998344	0.787900845
**EFN2B cg06826155**	0.056394152	0.070665119
**EFN2B cg11173146**	-0.002081738	0.825659813
**EFN2B cg11196529**	-0.028121827	0.620687015
**EFN2B cg11220502**	0.040529637	0.108591132
**EFN2B cg12053155**	0.00545394	0.650983134
**EFN2B cg12674145**	-0.001842116	0.9138715
**EFN2B cg13179469**	0.000881702	0.947367493
**EFN2B cg14363981**	0.009851043	0.397797952
**EFN2B cg17011964**	-0.11731264	0.002506895
**EFN2B cg17906168**	-0.093648467	5.07315E-07
**EFN2B cg18158859**	-0.051306079	0.308344274
**EFN2B cg18771357**	-0.000730228	0.961269374
**EFN2B cg19938857**	0.002066543	0.7769531
**EFN2B cg20256153**	0.033549064	0.002093283
**EFN2B cg22114393**	-0.00086411	0.942357692
**EFN2B cg22179913**	0.023524928	0.548464598
**EFN2B cg24929737**	-0.003133427	0.84260429
**EFN2B cg25634175**	-0.063312142	2.13094E-08
**EFN2B cg25814649**	-0.031777306	0.463465974
**EFN2B cg26138978**	-0.002862178	0.967604645
**EFN2B cg26273417**	0.091390186	2.8027E-06
**EFN2B cg27017630**	-0.021898491	0.112680531
**EFN2B cg27226949**	6.45625E-06	0.999707344

ıron, 2018)

Neuronal signaling signature refering to Fig. 2a, modified from Venkatarama		
**Gene with CpG site**	**FDC in high-neural glioblastoma**	**p-value**
GAP43 cg00216361	0.002160208	0.417707642
GAP43 cg01710607	-0.259086427	1.76834E-18
GAP43 cg03430502	-0.009590992	0.007127563
GAP43 cg05773207	-0.01098219	0.614582823
GAP43 cg05963821	0.005584014	0.23062102
GAP43 cg07863831	0.029287413	0.06143482
GAP43 cg10960118	-0.019828047	0.386205879
GAP43 cg11077657	-0.001389792	0.825182332
GAP43 cg11153239	-0.011915498	0.076421082
GAP43 cg12146703	-0.006456773	0.525109428
GAP43 cg13592373	-0.049917774	0.018175919
GAP43 cg14203525	0.001706367	0.558073643
GAP43 cg15143702	-0.088740817	1.91198E-06
GAP43 cg18849015	0.002829013	0.39649807
GAP43 cg19676835	0.000641705	0.878091622
GAP43 cg19836595	-0.027471916	0.003589394
GAP43 cg20914508	-0.007898534	0.001834675
GAP43 cg26783404	-0.043914503	0.244925411
GAP43 cg27608911	-0.011095859	0.024189657
GJA1 cg01493740	-0.002781633	0.788341899
GJA1 cg03181829	0.003895811	0.472272492
GJA1 cg10446179	0.000185843	0.966074645
GJA1 cg11486295	-0.136116895	3.54198E-15
GJA1 cg11590170	-0.009367987	0.292757126
GJA1 cg17160382	0.010539754	0.226109728
GJA1 cg17498101	3.23386E-06	0.999590775
GJA1 cg23699648	0.104176837	4.81048E-05
GJA1 cg24375465	-0.120116432	1.3509E-13
GRIA1 cg07633435	-0.151758428	4.01658E-09
GRIA1 cg08578734	-0.012793132	0.014838173
GRIA1 cg13583454	-0.17212038	4.95064E-09
GRIA1 cg16281600	-0.094676934	0.001431868
GRIA1 cg17020834	0.004054481	0.461276502
GRIA1 cg17987968	-0.064820218	0.006686229
GRIA1 cg20668321	-0.21041883	3.03028E-10
GRIA1 cg21146273	-0.095785641	0.000423115
GRIA1 cg22798121	-0.053159219	0.011738232
GRIA1 cg23165500	-0.13170471	2.3583E-10
GRIA1 cg26343183	0.022290145	0.269770752
GRIA2 cg00463631	0.001405683	0.828101379
GRIA2 cg00699993	-0.001384049	0.57469655
GRIA2 cg01041222	-0.008841612	0.006360613
GRIA2 cg01942962	-0.008917619	0.053016709

GRIA2 cg02757172	-0.071557833	4.70302E-07
GRIA2 cg08475096	-0.005409615	0.085695886
GRIA2 cg15425280	-0.009837536	0.006492941
GRIA2 cg16777106	-0.013403004	0.261409014
GRIA2 cg17605476	-0.014862348	6.90655E-05
GRIA2 cg22597733	-0.006590266	0.011513929
GRIA2 cg23734973	-0.012372876	0.001697911
GRIA2 cg24452260	-0.002297749	0.521065782
GRIA2 cg25148589	-0.01830372	0.00010535
GRIA2 cg26739975	-0.020256668	0.012204558
GRIA4 cg00343633	-0.002352325	0.684459277
GRIA4 cg00629688	-0.0937245	2.45131E-05
GRIA4 cg01111718	-0.119509724	1.58697E-14
GRIA4 cg03225817	0.004126215	0.488704456
GRIA4 cg03243226	-0.004974943	0.473359946
GRIA4 cg04747226	-0.001599855	0.899659663
GRIA4 cg05082609	-0.021759707	0.064248237
GRIA4 cg05087008	-0.211463343	1.51082E-07
GRIA4 cg06119894	-0.018249474	0.200818972
GRIA4 cg07592190	0.033934743	0.29570422
GRIA4 cg07972135	0.013899631	0.361949347
GRIA4 cg09147777	-0.007133644	0.249093769
GRIA4 cg09742895	-0.19149728	2.45253E-14
GRIA4 cg09974063	-0.011929026	0.10687749
GRIA4 cg09980522	-0.004992785	0.384030068
GRIA4 cg11067712	-0.002815147	0.382570623
GRIA4 cg11308643	-0.00767191	0.445576857
GRIA4 cg12754421	-0.005015184	0.509728282
GRIA4 cg15603568	-0.004181706	0.285962218
GRIA4 cg19343464	-0.003377691	0.769035573
GRIA4 cg20073686	-0.0060069	0.251650195
GRIA4 cg20306837	0.003256994	0.481766719
GRIA4 cg21217024	0.001057915	0.771112503
GRIA4 cg21719418	-0.129692321	1.00223E-13
GRIA4 cg23559689	-0.003973512	0.508051668
GRIA4 cg23676682	-0.000229298	0.976190919
GRIA4 cg27368776	-0.037478356	0.000659595
GRM1 cg03478199	-0.121679084	0.000887107
GRM1 cg06015218	-0.057134276	0.019000821
GRM1 cg06121352	-0.184765793	2.07636E-08
GRM1 cg06254938	-0.217607604	1.37771E-11
GRM1 cg07218663	-0.027242124	0.061077624
GRM1 cg07224114	-0.142635326	3.43176E-08
GRM1 cg08076125	-0.062918683	0.024447264
GRM1 cg08875948	0.008868627	0.85528093
GRM1 cg08958294	-0.053514313	0.02384755
GRM1 cg09179079	-0.031444941	0.014106782
GRM1 cg09423283	0.037860475	0.215595718
GRM1 cg09558195	-0.008417009	0.76906891

GRM1 cg10904109	-0.022821841	0.467103532
GRM1 cg13371951	-0.014161888	0.574100923
GRM1 cg13722123	-0.05130698	0.096482728
GRM1 cg13830799	-0.093750158	0.001944983
GRM1 cg14526297	-0.008219781	0.06816601
GRM1 cg16671069	-0.027376948	0.438544892
GRM1 cg17199007	-0.007485839	0.480124381
GRM1 cg19512268	-0.054850035	0.21656771
GRM1 cg19645248	-0.106893656	3.48577E-07
GRM1 cg21714266	0.073943351	0.046560464
GRM1 cg21725954	-0.019729909	0.428221839
GRM1 cg23696752	-0.023340768	0.452933871
GRM1 cg23753795	-0.168583236	7.03444E-14
GRM1 cg24266105	-0.058586578	0.003651697
GRM1 cg26256158	-0.004118379	0.759532618
GRM1 cg26875958	-0.117295166	4.55709E-10
GRM1 cg27642554	-0.033790759	0.287321565
GRM4 cg00070899	-0.118098163	3.12903E-07
GRM4 cg00143321	-0.00633863	0.208879882
GRM4 cg00369202	-0.16217027	5.7929E-10
GRM4 cg00898486	-0.118200949	2.25395E-07
GRM4 cg01081636	-0.132087187	1.80159E-08
GRM4 cg01090161	-0.210520059	1.92792E-15
GRM4 cg01140008	-0.161726202	1.34775E-07
GRM4 cg01242196	-0.173967626	1.15895E-12
GRM4 cg03412431	-0.011425121	0.012750455
GRM4 cg03723730	-0.075831598	0.002229662
GRM4 cg05745748	-0.008634077	0.03918362
GRM4 cg05800983	-0.039413413	0.111397352
GRM4 cg06068039	-0.151636963	7.05257E-09
GRM4 cg06112910	-0.070932595	5.01954E-07
GRM4 cg06456864	-0.052981669	0.004276194
GRM4 cg06898306	-0.211703673	3.93473E-25
GRM4 cg07699277	-0.007706223	0.482721422
GRM4 cg07735790	-0.009643776	0.682756735
GRM4 cg08289346	-0.011814591	0.754502321
GRM4 cg08969344	-0.227157136	1.01331E-14
GRM4 cg09106984	-0.162103538	7.89916E-11
GRM4 cg09657185	-0.044656299	0.028468246
GRM4 cg10083824	-0.029477764	0.492006313
GRM4 cg10270430	-0.049455938	0.066257004
GRM4 cg12008034	-0.092009319	8.91575E-05
GRM4 cg12013817	-0.025836377	0.060882077
GRM4 cg13344206	-0.11935417	3.11754E-07
GRM4 cg15884992	-0.060371029	0.005282731
GRM4 cg16739976	-0.084994484	9.05653E-06
GRM4 cg16916688	-0.099716587	0.000731875
GRM4 cg17107112	-0.062546309	8.19045E-07
GRM4 cg17450585	-0.091376308	9.94215E-06

GRM4 cg17762073	-0.058288459	0.00715518
GRM4 cg18403569	-0.043991037	0.01229403
GRM4 cg19471040	-0.069545066	0.031796964
GRM4 cg19999705	-0.009057552	0.754502321
GRM4 cg20151098	-0.025514198	0.00019797
GRM4 cg20490197	-0.087226066	9.44947E-06
GRM4 cg20772037	-0.170046947	1.16218E-08
GRM4 cg21599324	-0.100452044	1.59934E-08
GRM4 cg21797718	-0.050486447	0.038787205
GRM4 cg22188571	-0.151749201	1.90229E-11
GRM4 cg22484822	-0.071253561	2.9759E-07
GRM4 cg22827707	-0.01758895	0.005094544
GRM4 cg22971402	-0.207748277	4.67487E-13
GRM4 cg23321702	-0.180907785	2.47283E-12
GRM4 cg24082276	-0.011329932	0.729246104
GRM4 cg24469001	-0.198038965	2.96051E-15
GRM4 cg25988118	-0.015631512	0.627741446
GRM4 cg26424956	0.01407155	0.643236817
GRM4 cg27371466	-0.141039569	1.17477E-07
KCNN4 cg01424145	-0.031757952	0.015804254
KCNN4 cg01447572	-0.072850167	2.11212E-05
KCNN4 cg03731131	-0.054455535	0.049867038
KCNN4 cg04833845	-0.109605522	6.98316E-17
KCNN4 cg07756483	-0.008627508	0.048077996
KCNN4 cg10767665	-0.112232543	1.12951E-06
KCNN4 cg11624345	-0.10165581	0.000341148
KCNN4 cg14066757	-0.016462869	0.313611476
KCNN4 cg15977816	-0.027447837	0.041976459
KCNN4 cg18506018	-0.051023331	9.18501E-10
KCNN4 cg22904711	-0.08217768	0.000454803
KCNN4 cg26504422	-0.062946856	0.008961287
KCNN4 cg26890181	-0.028732752	0.020270245
KCNN4 cg26960896	-0.044758018	0.005541364
CTNND1 cg03813899	-0.010923239	0.523485939
CTNND1 cg06291428	0.012275871	0.322770902
CTNND1 cg09878888	-0.004167905	0.326563053
CTNND1 cg10831212	-0.002134478	0.688275465
CTNND1 cg11643991	-0.001485791	0.816420098
CTNND1 cg15068709	-0.099493969	2.49249E-06
CTNND1 cg16127573	-0.025102992	0.063253955
CTNND1 cg17345994	-0.204587885	1.15348E-15
CTNND1 cg18213545	-0.003998748	0.258781416
CTNND1 cg19210276	-0.005319634	0.332730467
CTNND1 cg19752551	-0.241684588	1.02152E-24
CTNND1 cg21272996	-0.002802617	0.584318545
THBS1 cg00027995	-0.213633043	1.23684E-11
THBS1 cg00836101	-0.082997055	0.00060386
THBS1 cg01637789	-0.047419731	0.003568321
THBS1 cg02291532	-0.175312769	5.6754E-11

THBS1 cg03196766	-0.132790048	4.52718E-06
THBS1 cg04051458	-0.005073449	0.554463337
THBS1 cg04827020	-0.205978433	7.8537E-21
THBS1 cg04888085	-0.015581265	0.148183749
THBS1 cg05886626	-0.00959143	0.739924766
THBS1 cg07276078	0.006147256	0.304048184
THBS1 cg08433504	-0.070448863	1.30291E-05
THBS1 cg09291834	0.00335148	0.730416233
THBS1 cg10078511	-0.067185095	9.20509E-05
THBS1 cg10548034	0.00387376	0.238037029
THBS1 cg10576133	-0.024452004	2.62162E-07
THBS1 cg12154418	-0.009461984	0.685870799
THBS1 cg12484072	0.001791023	0.656492
THBS1 cg13360562	-0.015728004	0.013045136
THBS1 cg13375187	-0.003454561	0.501233706
THBS1 cg13458407	0.00263783	0.883836151
THBS1 cg13463208	0.00281893	0.425642611
THBS1 cg14182667	-0.005987735	0.562747134
THBS1 cg14519850	0.007871194	0.517833352
THBS1 cg15161296	-0.013611524	0.042812336
THBS1 cg18234606	-0.129141163	2.11616E-06
THBS1 cg19570574	-0.02100468	0.073911634
THBS1 cg20043291	-0.005914958	0.596228526
THBS1 cg22580173	0.001134327	0.92283739
THBS1 cg23116540	-0.07817895	4.74352E-05
THBS1 cg23849826	-0.060244052	3.92179E-05
THBS1 cg25499543	-0.06310661	0.000316254
THBS1 cg25503381	-0.064681873	0.000148956
TTYH1 cg00558215	0.007562264	0.400484219
TTYH1 cg00813890	-0.008846858	0.183821647
TTYH1 cg02623991	-0.008447467	0.011277057
TTYH1 cg09474331	-0.00220203	0.727980426
TTYH1 cg10187559	-0.093855503	3.75816E-07
TTYH1 cg14814062	-0.043593604	0.001347461
TTYH1 cg15241920	0.005157608	0.414888588
TTYH1 cg15723536	0.016533162	0.024801481
TTYH1 cg15972301	-0.053519476	2.191E-05
TTYH1 cg20230372	0.004694511	0.612912157
TTYH1 cg20439295	-0.025157403	0.005169251
TTYH1 cg21287054	-0.027860128	0.100732522
TTYH1 cg21883754	0.000953189	0.78972954
TTYH1 cg22206370	-0.178235848	2.61253E-11
TTYH1 cg23695687	0.006548428	0.204270571
TTYH1 cg24687102	-0.033955528	0.162678037
HSPA5 cg01733783	-0.009615123	0.002511237
HSPA5 cg10997785	-0.058735772	0.000177855
HSPA5 cg13769004	-0.002944927	0.387728485
HSPA5 cg14294444	0.001083647	0.762010418
BDNF cg00298481	-0.0007783	0.802263288

BDNF cg01225698	-0.023347552	0.448425841
BDNF cg01583131	-0.079801494	0.008541296
BDNF cg01636003	-0.003393157	0.221916389
BDNF cg01642653	-0.025851388	0.399412129
BDNF cg02527472	-0.047100336	0.084941884
BDNF cg02613510	-0.097499624	0.001345417
BDNF cg03167496	-0.014494131	0.611164738
BDNF cg03747251	-0.00753236	0.551905897
BDNF cg03984780	-0.006151117	0.03051808
BDNF cg04106006	-0.007001098	0.889372737
BDNF cg04481212	-0.000298809	0.940072086
BDNF cg04672351	-0.001709511	0.43782095
BDNF cg05189570	-0.080699697	5.77462E-11
BDNF cg05218375	-0.008521356	0.082481117
BDNF cg05733135	-0.019445033	0.198732768
BDNF cg05818894	-0.007180117	0.459315357
BDNF cg06025631	-0.000704742	0.916576785
BDNF cg06046431	-0.04642876	0.03986164
BDNF cg06260077	0.007391204	0.430701617
BDNF cg06684850	-0.033224297	0.39039252
BDNF cg06816235	0.013211781	0.715547632
BDNF cg06979684	-0.040136789	0.037093702
BDNF cg06991510	0.001064034	0.789988073
BDNF cg07159484	0.002003335	0.755096665
BDNF cg07238832	-0.031708665	0.008002832
BDNF cg07704699	-0.047229239	0.010274325
BDNF cg08362738	-0.002255605	0.301859987
BDNF cg08388004	-0.013726092	0.002801484
BDNF cg09492354	-0.001637935	0.557909819
BDNF cg09606766	-0.001801528	0.652840898
BDNF cg10022526	0.012125721	0.690414601
BDNF cg10558494	-0.00138992	0.869721934
BDNF cg10635145	-0.092842502	0.003139264
BDNF cg11241206	-0.002083372	0.70576212
BDNF cg11718030	-0.047474508	0.194747456
BDNF cg11806762	-0.095742595	0.000178751
BDNF cg12448003	-0.003826584	0.883161786
BDNF cg13974632	-0.014283198	0.417133464
BDNF cg14291693	-0.124649778	8.55091E-11
BDNF cg14589148	-0.007091829	0.73582704
BDNF cg15014679	-0.226345125	6.04431E-17
BDNF cg15313332	0.008342035	0.573090265
BDNF cg15462887	-0.01401125	0.69601459
BDNF cg15688670	-0.0013428	0.682980226
BDNF cg15710245	-0.008415424	0.09905654
BDNF cg15914769	-0.001028228	0.690688888
BDNF cg16257091	-0.028366608	0.335709814
BDNF cg17413943	-0.010572907	0.055150561
BDNF cg18117895	0.001227892	0.818499274

BDNF cg18354203	-0.337982457	2.08378E-37
BDNF cg18595174	-0.024211822	0.330418904
BDNF cg20108357	-0.134330426	1.42622E-09
BDNF cg20340655	0.00156736	0.791941417
BDNF cg20954537	0.001005304	0.839469537
BDNF cg21010859	0.002277795	0.690442567
BDNF cg22043168	-0.033808445	0.059226322
BDNF cg23426002	-0.041353623	4.88695E-05
BDNF cg23497217	-0.000477465	0.910411408
BDNF cg23619332	-0.004768524	0.073343066
BDNF cg23947039	-0.001943328	0.763526094
BDNF cg24065044	-0.001722651	0.863695235
BDNF cg24249411	-0.046882007	0.130008164
BDNF cg24377657	-0.003725759	0.792044842
BDNF cg24650785	-0.016358801	0.564339899
BDNF cg25328597	-0.002149288	0.45371341
BDNF cg25381667	0.011413583	0.541908531
BDNF cg25412831	-0.014942703	0.746147456
BDNF cg25457956	0.01839504	0.361120777
BDNF cg25962210	-0.011269341	0.002128921
BDNF cg26057780	0.005913223	0.674635567
BDNF cg26840770	-0.003402862	0.314825821
BDNF cg26949694	0.017042802	0.62237618
BDNF cg27193031	-0.004134262	0.400462748
BDNF cg27351358	-0.088072122	0.000961796

ni et al. (Nat. Rev. Cancer, 2022)		

Cell culture data refering to Fig. 3a-d			
**Cell line**	**Idat-file tissue**	**Idat-file cell line**	**Year of surgery**
GS-10	204894630081_R04C01	204894630047_R06C01	2005
GS-11	204894630081_R06C01	204894630047_R07C01	2005
GS-12	204894630081_R08C01	204894630047_R08C01	2005
GS-13	206238130095_R02C01	204894630060_R01C01	2006
GS-57	203282450014_R05C01	202277800135_R04C01	2014
GS-73	203282450110_R03C01	203282450110_R02C01	2016
GS-74	205735130138_R05C01	203282450110_R05C01	2016
GS-75	205689130089_R04C01	206466470159_R03C01	2016
GS-80	204361730148_R07C01	203282450110_R08C01	2017
GS-83	205689130089_R03C01	206466470159_R04C01	2017
GS-84	204294120040_R01C01	206466470159_R06C01	2017
GS-85	203282450014_R08C01	203282450014_R07C01	2017
GS-90	202277800135_R08C01	202277800135_R07C01	2018
GS-101	203939360028_R01C01	203939360028_R02C01	2019
GS-106	205689130179_R03C01	206466470159_R05C01	2019
GS-110	204081740052_R06C01	206466470159_R07C01	2020
NCH 551b	204798720012_R05C01	204316900118_R05C01	2020

**Patients age at surgery**	**Sex**
63	male
50	female
69	female
45	female
59	male
66	male
73	male
69	female
60	male
66	male
51	male
51	female
63	female
77	female
52	male
72	male
68	male

Patient-derived xenograft data refering to Fig. 3e			
**PDX line**	**Idat-file tissue**	**Neural signature score**	**Neural subgroup**
GS-8	204894630041_R08C01	0.388	Low
GS-10	204894630081_R04C01	0.385	Low
GS-73	203282450110_R03C01	0.335	Low
GS-57	203282450014_R05C01	0.569	High
GS-74	205735130138_R05C01	0.528	High
GS-75	206466470159_R03C01	0.415	High
GS-101	203939360028_R02C01	0.474	High

**Year of surgery**	**Patients age at surgery**	**Sex**
2006	67	female
2005	63	female
2016	66	male
2014	59	male
2016	73	male
2016	73	male
2019	77	female

Cell culture data refering to Supplementary Fig. 6			
**Cell line**	**Idat-file**	**Neural signature score**	**Neural subgroup**
GS-11	204894630081_R06C01	0.343	Low
GS-73	203282450110_R03C01	0.335	Low
GS-84	204294120040_R01C01	0.4	Low
GS-110	204081740052_R06C01	0.396	Low
GS-13	206238130095_R02C01	0.627	High
GS-74	205735130138_R05C01	0.42	High
GS-80	204361730148_R07C01	0.532	High
GS-90	202277800135_R08C01	0.467	High
GS-101	203939360028_R01C01	0.474	High

Year of surgery	Patients age at surgery	Sex
2005	50	female
2016	66	male
2017	51	male
2020	72	male
2006	45	female
2016	73	male
2017	60	male
2018	63	female
2019	77	female
