# Supplementary material for: A prognostic neural epigenetic signature in high-grade glioma
Source: Nat Med. 2024 May 17;30(6):1622–35. doi: 10.1038/s41591-024-02969-w (PMC11186787; doi:10.1038/s41591-024-02969-w)
Supplement: Supplementary file 2 — Reporting Summary [file 41591_2024_2969_MOESM2_ESM.pdf]

nature portfolio

Corresponding author(s): Franz L. Ricklefs

Last updated by author(s): 03/19/2024

Reporting Summary

Nature Portfolio wishes to improve the reproducibility of the work that we publish. This form provides structure for consistency and transparency in reporting. For further information on Nature Portfolio policies, see our Editorial Policies and the Editorial Policy Checklist.

Statistics

For all statistical analyses, confirm that the following items are present in the figure legend, table legend, main text, or Methods section.

n/a	Confirmed	
☐	☒	The exact sample size (*n*) for each experimental group/condition, given as a discrete number and unit of measurement
☐	☒	A statement on whether measurements were taken from distinct samples or whether the same sample was measured repeatedly
☐	☒	The statistical test(s) used AND whether they are one- or two-sided<br>*Only common tests should be described solely by name; describe more complex techniques in the Methods section.*
☐	☒	A description of all covariates tested
☐	☒	A description of any assumptions or corrections, such as tests of normality and adjustment for multiple comparisons
☐	☒	A full description of the statistical parameters including central tendency (e.g. means) or other basic estimates (e.g. regression coefficient) AND variation (e.g. standard deviation) or associated estimates of uncertainty (e.g. confidence intervals)
☐	☒	For null hypothesis testing, the test statistic (e.g. *F*, *t*, *r*) with confidence intervals, effect sizes, degrees of freedom and *P* value noted<br>*Give P values as exact values whenever suitable.*
☒	☐	For Bayesian analysis, information on the choice of priors and Markov chain Monte Carlo settings
☒	☐	For hierarchical and complex designs, identification of the appropriate level for tests and full reporting of outcomes
☐	☒	Estimates of effect sizes (e.g. Cohen's *d*, Pearson's *r*), indicating how they were calculated

Our web collection on statistics for biologists contains articles on many of the points above.

Software and code

Policy information about availability of computer code

Data collection	3D volumetric segmentation was processed using BRAINLAB Elements software (v3.0). Illumina EPIC (850k) array was used for analyzing genome-wide DNA methylation patters of tumor tissues. For proteomic data processing, Proteome Discoverer 3.0. was used and searched against a reviewed FASTA database (UniProtKB: Swiss-Prot, Homo sapiens, February 2022, 20300 entries). Analysis of serum flow cytometry data was performed using the BioLegend LEGENDplex software. Cell counting of confocal images was conducted with ImageJ. Next-generation sequencing were done by a 201 gene panel (Agilent, SureSelect Custom Tier2, 1.235Mbp).Sequenced reads were mapped to GRCh38 using the nf-core/sarek (v3.3.2) pipeline. SNV and Structural variant calling was done using Strelka (v4.4.0.0) and Manta (v1.6.0).Annotation of the detected variants was performed using SNPeff (v5.1d).
Data analysis	Methylation arrays were processed with R (version 1.40.0). Integrative analysis of methylation and gene expression was performed with hdWGCNA's ConstructNetwork function. For single-cell data analysis, the AddModuleScore function of the Seurat package was used and projected to the cell-level UMAP (Uniform Manifold Approximation and Projection) provided by GBMap's integration algorithm. Computational analysis of spatially resolved transcriptomic data was employed by the SPATA2 package (v2.01) and spatial correlation analysis was performed by the MERINGUE package. Visualization of the analysis was done using the ggraph package. Cell state composition analysis of DNA methylation arrays was performed via the engine provided in EpiDISH package. Absolute tumor-purity of DNA methylation arrays was calculated using the RF_purify Package in R.Statistical analyses were conducted using GraphPad Prism v10. Confocal image analyses was done using ImageJ v.2.9.0. Data analysis of drug sensitivity was performed using staining using CellProfiler 2.2.0 and MATLAB R2021b. Seurat was used for single-cell data analysis. Magnetencephalography recordings were analyzed using NUTMEG software suite version 4. Functional data from resting state MRI was analyzed were preprocessed using SPM12 as implemented in MATLAB (Version: 9.13.0 (R2022b) Update 2). Single cell transcriptomc data were analyzed through the GBMap reference dataset.

For manuscripts utilizing custom algorithms or software that are central to the research but not yet described in published literature, software must be made available to editors and reviewers. We strongly encourage code deposition in a community repository (e.g. GitHub). See the Nature Portfolio guidelines for submitting code & software for further information.

Data

Policy information about availability of data

All manuscripts must include a data availability statement. This statement should provide the following information, where applicable:

- Accession codes, unique identifiers, or web links for publicly available datasets
- A description of any restrictions on data availability
- For clinical datasets or third party data, please ensure that the statement adheres to our policy

IIdat files of the clinical cohort (363 glioblastoma patients) are available on Gene Expression Omnibus (GEO) under GSE240704. The methylation data provided by Capper et al. as illustrated in Extended Data 1 are accessible under GSE109381. TCGA-GBM cohort analyzed for external validation and as shown in Figure 1d is accessible under https://portal.gdc.cancer.gov/projects/TCGA-GBM. Data files used in the spatial transcriptomic analyses are accessible under https://doi.org/10.5281/zenodo.10863736. Single-cell RNAseq dataset GBMap is available from the original publication and can be accessed through cellXgene (https://cellxgene.cziscience.com/collections/999f2a15-3d7e-440b-96ae-2c806799c08c) and human motor cortex single cell RNAseq dataset is available from Allen Brain Institute at https://portal.brain-map.org/atlases-and-data/rnaseq/human-m1-10x.

Research involving human participants, their data, or biological material

Policy information about studies with human participants or human data. See also policy information about sex, gender (identity/presentation), and sexual orientation and race, ethnicity and racism.

Reporting on sex and gender	The human datasets used in this study include patients of both sexes (male and female) which applied reported findings to both sexes. Furthermore, patients sex was taken into consideration as a prognostic variable for survival analyses and details are reported in Supplementary Table 1.
Reporting on race, ethnicity, or other socially relevant groupings	Does not apply to our human datasets.
Population characteristics	All patients described in this study are individuals diagnosed with glioblastoma or diffuse midline glioma. Clinical characteristics are listed in Supplementary Table 1 and contain known variables that might influence survival (as the primary outcome of this study), such as age, Karnofsky Performance Status, sex, MGMT status, and location.
Recruitment	The inclusion criteria for this research were patients diagnosed with either IDH-wildtype glioblastoma or H3 K27-altered diffuse glioma, who qualified for methylation analysis and provided informed consent. Exclusion criteria included cases with invalid DNA methylation data, those lost to follow-up, and additional specifications outlined in the methods section. The selection process was unbiased, focusing exclusively on the patients' diagnoses without regard to other variables.
Ethics oversight	This study complied with all relevant ethical regulations and experiments were approved by the medical ethics committee of the Hamburg chamber of physicians (PV4904, Hamburg, Germany).

Note that full information on the approval of the study protocol must also be provided in the manuscript.

Field-specific reporting

Please select the one below that is the best fit for your research. If you are not sure, read the appropriate sections before making your selection.

☒ Life sciences ☐ Behavioural & social sciences ☐ Ecological, evolutionary & environmental sciences

For a reference copy of the document with all sections, see nature.com/documents/nr-reporting-summary-flat.pdf

Life sciences study design

All studies must disclose on these points even when the disclosure is negative.

Sample size	The research focused on patients who had surgery for either glioblastoma or diffuse midline glioma. No specific sample size calculation was applied for clinically oriented patient groups, with inclusion extending to all patients listed in the institutional databases. Selection criteria for participation are comprehensively outlined in the Methods section.<br>In regards to in vitro or in vivo data: all experiments were performed in triplicates or quintuplicates in accordance to international standards.
Data exclusions	No data were excluded from the analyses.
Replication	All presented experiments were performed in triplicates if not otherwise stated. Findings were reproducible with biological replicates performed on separate animals/cells and external cohorts for patients.
Randomization	Patients, animals, and cell cultures were separated into a low-neural and high-neural group based on the neural signature score calculated from each individual DNA methylation array.

Blinding

Data collection of clinical outcomes and methylation profiles were acquired by different research teams. This data was combined for analysis only after the samples had been processed, enabling a coherent examination of the findings. Moreover, the linkage between clinical information and methylation signatures was concealed from all investigators to ensure objectivity.

Reporting for specific materials, systems and methods

We require information from authors about some types of materials, experimental systems and methods used in many studies. Here, indicate whether each material, system or method listed is relevant to your study. If you are not sure if a list item applies to your research, read the appropriate section before selecting a response.

Materials & experimental systems

n/a	Involved in the study	
☐	☒	Antibodies
☐	☒	Eukaryotic cell lines
☒	☐	Palaeontology and archaeology
☐	☒	Animals and other organisms
☐	☒	Clinical data
☒	☐	Dual use research of concern
☒	☐	Plants

Methods

n/a	Involved in the study	
☒	☐	ChIP-seq
☒	☐	Flow cytometry
☐	☒	MRI-based neuroimaging

Antibodies

Antibodies used

Primary antibodies used:
- Mouse anti-human nuclei clone 235-1 (1:100; Millipore)
- rabbit anti-Ki67 (1:500; Abcam ab15580)
- rat anti-MBP (1:200, Abcam ab7349)
- mouse anti-nestin (1:500; Abcam ab6320)
- guinea pig anti-synapsin1/2 (1:500; Synaptic Systems)
- chicken anti-neurofilament (M+H; 1:1000; Aves Labs)
- chicken anti-PSD95 (1:500, Abcam ab18258)
- mouse anti-vimenting AF488 conjugated (1:1000, Biolegend clone O91D5)
- rabbit anti-cleaved caspase 3 (1:1000; Cell signaling #9579)
- mouse anti-TUBB3 (1:1000, Biolgend clone AA10)
- abbit anti-BDNF (1:1000, Cell signaling #47808)
- mouse anti-beta-actin (1:1000, Sigma Aldrich A2228)
- NeuN (#MAB377, Chemico, 1:200)
- Sox2 (#AB79351, Abcam, 1:200)
- OLIG2 (#AF2418, R&D Systems, 1:50)
- GFAP (#M0761, DAKO, 1:200)

Secondary antibodies used:
- Alexa 488 donkey anti-rabbit IgG (Jackson ImmunoResearch, 711-545-152)
- Alexa 594 donkey anti-mouse IgG (Jackson ImmunoResearch, 715-585-150)
- Alexa 647 donkey anti-chicken IgG (Jackson ImmunoResearch, 703-605-155)
- Alexa 405 donkey anti-guinea pig IgG (Jackson ImmunoResearch, 706-475-148)
- Alexa 647 donkey anti-rabbit IgG (Jackson ImmunoResearch, 711-605-152)
- Alexa 594 donkey anti-mouse IgG (Jackson ImmunoResearch, 715-585-150)

Primary antibody used in immuno-electron microscopy:
- goat anti-RFP (1:300; #ABIN6254205 Antibodies-online Inc.; Lot#0040180316) and secondary antibody (1:10; #15796 TED Pella; Lot #008330)

Validation

All above mentioned antibodies were purchased from commercial vendors and were validated by the manufacturers and used in accordance to previous studies:
1. anti-human nuclei clone (Milipore): PMID 31534222
2. anti-Ki67 (Abcam): PMID 31534222
3. rat anti-MBP (1:200, Abcam ab7349): PMID 32433967
4. anti-nestin (Abcam): PMID 31534222
5. anti-synapsin 1/2 (Synaptic Systems): validated in IHC and IHC-P by provider.
6. anti-neurofilament (M+H) (Aves Labs): PMID 31534222
6. anti-PSD95 (Abcam): validated in IHC-P by provider
7. anti-vimentin (Biolegend): PMID: 35022622
8. anti-cleaved Caspase 3 (Cell signaling): PMID: 7596430
9. anti- TUBB3 (Biolegend): PMID: 35022622
10. anti-BDNF (Cell signaling): PMID: 35595779

Eukaryotic cell lines

Policy information about cell lines and Sex and Gender in Research

Cell line source(s)	All presented cell lines obtained from primary patient-derived glioblastomas were generated in the Department of Neurosurgery, University Medical Center Hamburg-Eppendorf (Germany). These cell lines include: GS-8; GS-10; GS-11; GS-12; GS-13; GS-57; GS-74; GS-74; GS-75; GS-80; GS-83; GS-84; GS-85; GS-90; GS-101; GS-106; GS-110; NCH 551b. Additionally, the low-neural cell line ("SF-HH-1") and high-neural cell line ("SF-HH-2") derived from patient-derived glioblastomas of the Department of Neurosurgery, University of California, San Francisco (UCSF) and were processed in the Monje Lab, Stanford University.
Authentication	Short Tandem Repeat fingerprinting or whole genome methylation analysis was performed every 8 weeks on all cell cultures,
Mycoplasma contamination	All cell cultures were tested negative for possible contamination every 2 weeks.
Commonly misidentified lines (See ICLAC register)	No commonly misidentified lines were used.

Animals and other research organisms

Policy information about studies involving animals; ARRIVE guidelines recommended for reporting animal research, and Sex and Gender in Research

Laboratory animals	Following mice were used for various experiments:<br>- NOD-SCID-IL2R gamma chain-deficient Jackson Laboratory) for assessing tumor burden and electron microscopy; 4-6 wekks<br>- NMRI-Foxn1nu immunodeficient mice (Janvier-Labs) for survival studies, 6-8 weeks<br>- CD1 (Jackson Laboratories) for isolation of neurons, 4-6 weeks<br><br>Animals were housed according to standard guidelines under pathogen-free conditions, in temperature (28-30°C)- and humidity (45-50%)-controlled housing with free access to food and water in a 12 h light:12 h dark cycle.
Wild animals	No wild animals were used.
Reporting on sex	Both male and female mice were used for all in vivo experiments.
Field-collected samples	No field-collected samples were used.
Ethics oversight	In Vivo studies were approved by the authorities for health and consumer protection in Hamburg, Germany (#17.8.17), as well as by the Stanford University Institutional Animal Care and Use Committee (IACUC) #30342.

Note that full information on the approval of the study protocol must also be provided in the manuscript.

Clinical data

Policy information about clinical studies

All manuscripts should comply with the ICMJE guidelines for publication of clinical research and a completed CONSORT checklist must be included with all submissions.

Clinical trial registration	n/a
Study protocol	n/a
Data collection	Clinical data of glioblastoma and diffuse midline glioma patients were retrieved from institutional retrospective databases. Included patients underwent surgery between 2009 and 2023.
Outcomes	Primary outcomes of clinical data were overall survival and progression-free survival. Overall survival (OS) was calculated from diagnosis until death or last follow-up, and progression-free survival (PFS) from diagnosis until progression according to Response Assessment in Neuro-Oncology (RANO) criteria based on local assessment (DOI: 10.1200/JCO.2009.26.3541)

Magnetic resonance imaging

Experimental design

Design type	Resting-state
Design specifications	44 treatment-naîve glioblastoma patients (mean age: 65±9 years) underwent resting-state functional magnet resonance imaging before surgery.Functional data were preprocessed using SPM12 as implemented in MATLAB (Version: 9.13.0 (R2022b) Update 2) according to an imaging protocol. Functional images were realigned to the mean functional volume, unwarped and coregistrated to the structural image.

Behavioral performance measures | No behaviour perfomance measures were used.

Acquisition

Imaging type(s): Structural MRI, functional MRI

Field strength: 3T

Sequence & imaging parameters: -Sagittal 3D-T1 magnetization-prepared rapid acquisition gradient echo (MPRAGE), TR = 2300ms, TE = 2.3200ms, number of slices = 192 with 0.9mm slice thickness, flip angle = 8°, FoV = 245mm, voxel size = 0.9375x0.9375x0.9mm.
-Echo planar imaging (EPI), including 300 whole-brain functional volumes, TR = 2200ms, TE = 30 ms, number of slices = 36 with 3.1mm slice thickness, flip angle = 90°, FoV = 1200mm, voxel size = 3.125x3.125x3.565mm.

Area of acquisition: Whole brain

Diffusion MRI ☐ Used ☒ Not used

Preprocessing

Preprocessing software: SPM12 as implemented in MATLAB (Version: 9.13.0 (R2022b) Update 2)

Normalization: Images were segmented, bias corrected and spatially normalized (multi-spectral classification), and functional images were smoothed with a 5 mm FWHM Gaussian kernel.

Normalization template: MNI 1.5mm isotropic, as implemented in SPM 12's Normalization procedure

Noise and artifact removal: Functional images were slice-time corrected, movement-related time series were regressed out with ICA-AROMA, and data were high-pass filtered (> 0.01 Hz).

Volume censoring: ITK-SNAP software; the segmentated volumes were reviewed independantly from two experts in the field.

Statistical modeling & inference

Model type and settings: Contrast-enhancing tumor lesions were segmented semi-automatically using the ITK-SNAP software version 3.4.077 and used as region of interest (ROI) to perform a seed-based correlation analysis and compute the voxel-based tumor to peritumoral connectivity (Fisher z transformation). A 10mm peritumoral distance mask was created by dilating the tumor mask by 10mm and subtracting the tumor area. The mean functional connectivity between tumor and its 10mm peritumoral surrounding was computed after ROI-to-voxel analyses.

Effect(s) tested: n/a

Specify type of analysis: ☐ Whole brain ☐ ROI-based ☒ Both

Anatomical location(s): Contrast-enhancing tumor lesions were segmented semi-automatically using the ITK-SNAP software version 3.4.0; further anatomical locations were not used

Statistic type for inference: Voxel-wise

(See Eklund et al. 2016)

Correction: FDR

Models & analysis

n/a	Involved in the study
☐	☒ Functional and/or effective connectivity
☒	☐ Graph analysis
☒	☐ Multivariate modeling or predictive analysis

Functional and/or effective connectivity: Pearson correlations were computed between the tumor (ROI as seed) and all other voxels in the brain (seed-based functional connectivity). Correlations were then Fisher z-transformed and thresholded at z > .2.
