# Supplementary figures and images for: A prognostic neural epigenetic signature in high-grade glioma
Source: Nat Med. 2024 May 17;30(6):1622–35. doi: 10.1038/s41591-024-02969-w (PMC11186787; doi:10.1038/s41591-024-02969-w)

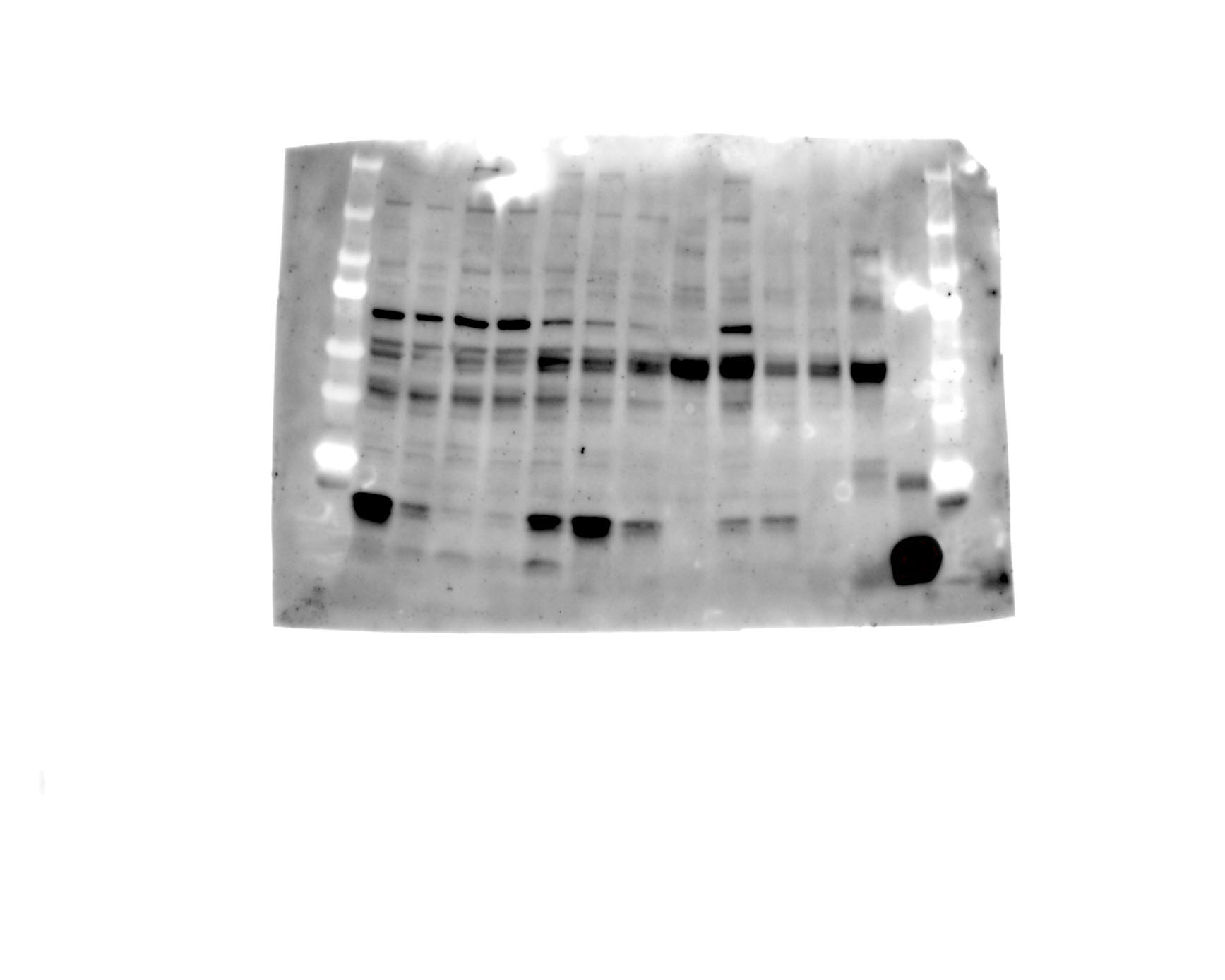

Supplement: Supplementary file 3 — BDNF western blot. [file 41591_2024_2969_MOESM3_ESM.jpg]

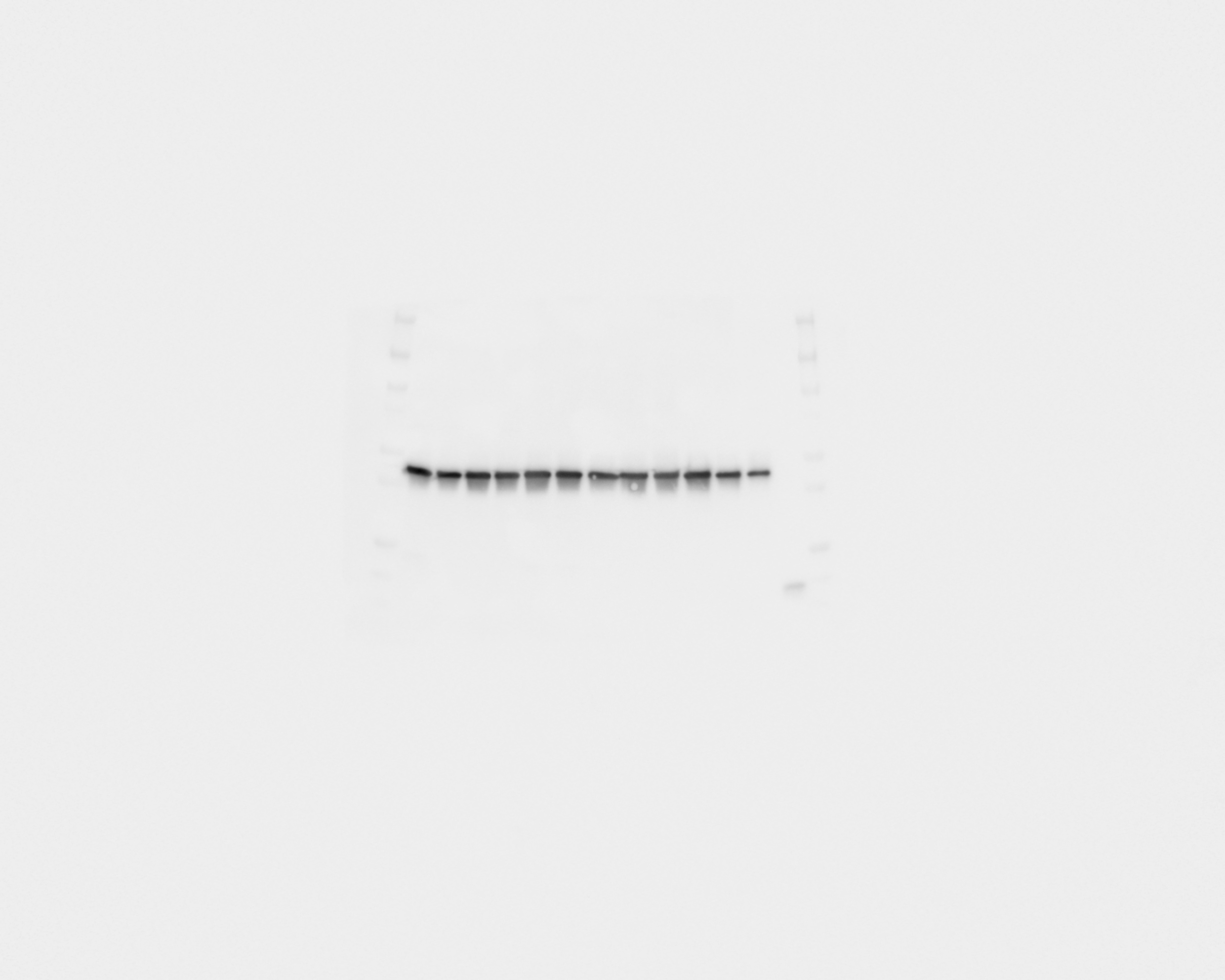

Supplement: Supplementary file 4 — GAPDH western blot. [file 41591_2024_2969_MOESM4_ESM.jpg]
